# Supplementary material for: Short-term and long-term cost-effectiveness of a pedometer-based exercise intervention in primary care: a within-trial analysis and beyond-trial modelling
Source: BMJ Open. 2018 Oct 17;8(10):e021978. doi: 10.1136/bmjopen-2018-021978 (PMC6196874; doi:10.1136/bmjopen-2018-021978)
Supplement: Supplementary data [file bmjopen-2018-021978supp001.pdf]

Supplementary file for

“The short-term and long-term cost-effectiveness of a pedometer-based intervention in primary care: A within-trial analysis and beyond-trial modelling”

Nana Anokye PhD, Julia Fox-Rushby PhD, Sabina Sanghera PhD, Derek G. Cook PhD, Elizabeth Limb MSc, Cheryl Furness MSc, Sally Kerry PhD, Christina Victor PhD, Steve Iliffe FRCGP, Michael Ussher PhD, Peter H.Whincup PhD, Ulf Ekelund PhD, Steve DeWilde PhD, Tess Harris MD

Table S1: Resource use and cost components of ‘Set-up Cost’*

**Activity (trial arm applicable to)**	**Resource**	**Total quantity**	**Cost per particip ant £ (nurse group)**	**Cost per particip ant £ (post group)**
**Design^**				
Designing of intervention (Both intervention arms)	Professor x1	0·5 days	4·43	4·43
	Readers x3	1 day		
	Senior lecturers x3	3·5 days		
	Consultants x2	1 days		
Designing of participants’ handbooks and diaries (both intervention groups)	Professor x3	1·5 days	3·56	3·56
	Readers x2	1 day		
	Senior lecturers x3	2 days		
	Consultants x2	0·5 days		
Designing of nurse trainers handbooks (Nurse group)	Senior lecturers x1	1 day	2·74	0
	Consultants x1	0·5 days		
	Handbooks	9 handbooks	0·19	0
**Setting up GP practices**				
Planning for recruitment of practices (All trial arms)	Professor x1	1 hour	0·99	0·99
	Senior lecturer x1	5 hours		
	Consultants x2	5 hours		
Visits to recruit 6 practices (All trial arms)	Senior lecturers x2	13 hours	1·47	1·47
	Trial Manager x1	7 hours		
	Consultant x1	5 hours		
	Round trips to practices (by all)	25 hours	0·10	0·10
Searching practice computers to identify participants (All trial arms)	Senior lecturer x1	6 hours	0·71	0·71
	Trial Manager x1	6 hours		
	Practice Manager x6	6 hours		
Identify households from anonymised address list (All trial arms)	Senior lecturer x1	32 hours	2·28	2·28
	Trial Manager x1	32 hours		
Practice staff reviews lists for exclusion (All trial arms)	GP x5 (for sorting out 2 practices)	20 hours	4·50	4·0

Activity (trial arm applicable to)	Resource	Total quantity	Cost per participant £ (nurse group)	Cost per participant £ (post group)
	Nurse x10 (for sorting out other 5 practices)	50 hours	1·96	1·96
Printing letters at practice (All trial arms)	Trial Manager x1	64 hours	1·57	1·57
	Practice administrative staff x2	4 hours		
	Number of printed letters	24000	0·94	0·94
Packing envelopes with leaflets and letters (All trial arms)	Trial Manager x1	240 hours	7·04	7·04
	Research Assistants x2	56 hours		
	Practice admin. Staff x11	27·5 hours		
	Cost of Envelopes	£497·30	0·49	0·49
	Cost of Postal stamps	£5,530·50	5·41	5·41
	Cost of Information leaflets	£5,973·00	5·84	5·84
Preparing rooms at practices for trial (All trial arms)	Round trip to practices by RA	14 trips	0·04	0·04
	Research Assistants x2	-*	0·11	0·11
**Training**				
Training of Trial manager (All trial arms)	Trial Manager x1	4 days	1·51	1·51
	Senior lecturer x1	2 days		
Preparation of nurse training course (Nurse support group)	Trial Manager x1	1 day	9·63	0
	Senior lecturer x1	2 days		
	Reader x1	0·5 days		
	Consultants x2	2 days		
Mini-training day of nurses (Nurse group)	Nurses x11	33 hours	7·46	0
	Trial Manager x1	17·33 hours		
	Senior lecturer x1	17·33 hours		
	Round trips to training centre (by tutors)	16 hours	0·19	0
	Pedometers given to nurses	12 hours	0·04	0
Full training day of nurses (Nurse group)	Nurses x10	107·5 hours	22·99	0
	Reader x1	1 hour		
	Senior lecturer x1	10 hours		
	Consultants x2	22·5 hours		
	Round trips for training by nurses x10	10 trips	0·12	0
	Round trips for training by consultants x2	2 trips	0·13	0
	Refreshments	1 set	0·26	0
Training for an absentee nurse (Nurse group)	Nurse x 1	10 hours	2·47	0
	Trial Manager x1	11·33 hours		
	Research assistant x1	11·33 hours		
	Round trips to training centre	2 trips	0·02	0
Discussion of nurses recorded sessions(Nurse group)	Senior lecturer x1	0·5 days	3·78	0
	Consultants x2	1 day		
	Nurses x9	4·5	0·99	0
	Senior lecturer x1	0·5		

**Activity (trial arm applicable to)**	**Resource**	**Total quantity**	**Cost per particip ant £ (nurse group)**	**Cost per particip ant £ (post group)**
	Consultants x2	1		
	Duration of phone calls	270 mins	0·09	0
Follow-up half day training(Nurse group)	Nurses x 9	4.5 days	7·70	0
	Trial Manager x1	0·5 days		
	Senior lecturer x1	0·5 days		
	Consultants x2	1 day		
	Nurse time travelling x 9	6·75 hours	0·78	0
	Round trips to training centre (nurses)	9 trips	0·10	0
	Refreshment	1 set	0·15	0
Training of Research assistants (All trial arms)	Research assistant x3	6·6 days	1·91	1·91
	Senior lecturer x1	0·5 days		
	Reader x1	0·5 days		
	Trial Manager x1	4 days		
Total cost per participant			104·64	44·83

^ Design was included as materials couldn't be used wholesale from a previous study and we judged that this may occur in the future following further learning from this trial*Value removed at present to maintain confidentiality
*Data source: Interviews with trial PI and trial manager, review of trial records, diaries, and routine administrative records

Table S2: Components of delivery cost of intervention (Post group)

Components	Resource (from administrative records)	Quantity of resource	Unit cost (data source)	3 months analysis		12 months analysis	
				Total cost	Cost per participant	Total cost	Cost per participant
Envelopes for posting pedometers (including replacement)	Number of envelopes	426	£0·03 (invoice)	£12·78	£0·04	£12·78	£0·04
Stamps for posting pedometer	Number of stamps	426	£2·50 (invoice)	£1,065	£3·14	£1,065	£3·14
Pedometers (including replacements) given to participants	Number of pedometers	426	£1 / £4*(invoice)	£426	£1·26	£1,704	£5·03
Replacement batteries for pedometer	Number of replacement batteries	11	£0·67 (invoice)	£7·37	£0·02	£7·37	£0·02
Patient handbooks	Number of handbooks	339	£0·80 (administrative records)	£271	£0·80	£271	£0·80
Step count diary	Number of diaries	339	£1·30 (administrative records)	£440·70	£1·30	£440·70	£1v30
Total cost per participant					£6·56		£10·33

*£1 was pro rata unit cost for 3 months and £4 is for 12 months. As pedometers were required only for the period of analysis but could be used beyond, their costs were spread over their expected lifetime, following Sharples et al (2014)[1]. As pedometers had an expected lifetime of 2 years, the average cost of pedometer was multiplied by 13[1]/104[2](weeks), in the case of 3 month analysis and 52/104 for the 12 month analysis.

[1] Intervention period in weeks

[2] Life expectancy of pedometer (in weeks)- based experience from PACE lift trial

Table S3: Components of delivery cost of intervention (Nurse group)

Components	Resource (data source)	Quantity of resource	Unit cost (data source)	3 months analysis		12 months analysis	
				Total cost	Cost per participant	Total cost	Cost per participant
Pedometers given to participants	Number of pedometers (administrative records)	346	£1 / £4* (Invoice)	£346	£1	£1384	£4
Patient handbooks	Number of handbooks (administrative records)	346	£0·80 (administrative records)	£277	£1	£277	£1
Step count diary	Number of diaries (administrative records)	346	£1·30 (administrative records)	£449·80	£1·30	£449·80	£1·30
RAs time to arrange consultation	Time spent by RAs (diary)	50·46 hours	£16·51 (administrative records)	£833·07	£2·41	£833·07	£2·41
Phone calls by RA to arrange consultation	Duration of phone calls (administrative records)	3,027·5 mins	£0·11 (BT tariff)	£333·03	£0·96	£333·03	£0·96
Cost of nurse visit per participant (project database for nurse group)					£43		£42
Total cost per participant					£49·67		£51·67

*£1 was pro rata unit cost for 3 months and £4 is for 12 months.

Table S4: Costs to participants of participating in interventions and physical activity

Participant costs	Control (n=323)	Post (n=312)	Nurse (n=321)
	£ Mean (SD)		
Intervention related			
Time working out how to use pedometer	0(0)	2 (6)	1 (3)
Time planning how to increase walking/step count	0(0)	5 (15)	3 4)
Time filling in PACE-UP diary	0(0)	51 (80)	58 (122)
Parking fees to visit nurse	0(0)	0(0)	0·11 (0.73)
Time spent in consultation with nurse	0(0)	0(0)	10 (5)
Time travelling (irrespective of mode of transport) to visit nurse	0(0)	0(0)	11 (10)
Transportation cost (for those who took public transport) of attending the nurse visit	0(0)	0(0)	0·13 (1.33)
Time waiting time prior to consultation with nurse	0(0)	0(0)	3 (4)
Child care during nurse visits	0(0)	0(0)	0·3 (3.21)
Personal costs of participation in physical activity	411 (817)	492 (1,293)	333 (684)
Personal costs from falls/ fractures/ sprains/ injuries	17 (103)	22 (184)	6 (40)

Table S5: Health service use by trial arm with unit costs

Health service use	Trial arm (Qty)			Unit cost (£) Weighted average (Q1 – Q3)	Source of unit cost
	Control n=323	Post n=312	Nurse n=321		
**Outpatient referrals (total)[2]**	**164**	**158**	**186**		
Opthalmology	10	18	15	86 (70-99)	DH (2015) National Reference Costs
Urology	4	3	6	99 (76-116)	
General medicine	4	0	2	157 (120-187)	
ENT	9	6	12	92 (70-109)	
Podiatry	9	7	7	44 (27-45)	
Trauma & orthopaedics	14	13	10	113 (88-133)	
Physiotherapy	26	33	37	46 (35-50)	
Nephrology	0	1	0	145 (94-178)	
Oral surgery	0	2	0	115 (85-142)	
Gynaecology	6	7	14	134 (104-164)	
Audiology	4	6	7	104 (55-174)	
Colorectal surgery	1	5	1	117 (83-135)	
Neurology	8	8	5	174 (136-204)	
Cardiology	12	5	4	131 (92-154)	
Gastroenterology	6	2	6	130 (99-153)	
Rheumatology	4	6	7	135 (99-150)	
Dermatology	1	8	7	98 (74-109)	
General surgery	4	1	3	125 (98-165)	
Endocrinology	2	1	2	144 (100-167)	
Neurosurgery	2	0	0	181 (138-228)	
Oncology	8	5	11	133 (97-165)	
Psychotherapy	1	0	0	100 (47-217)	
Respiratory medicine	4	6	3	150 (107-181)	
Clinical neurophysiology	2	0	1	165 (107-197)	
Programmed pulmonary rehab	0	0	1	20 (12-31)	
Pain management	2	0	4	135 (82-164)	
Allergy service	0	1	0	149 (126-175)	
Dietetics	2	2	3	62 (38-76)	
Vascular surgery	2	1	4	149 (100-176)	
Mental illness	1	1	1	234 (181-256)	
Clinical Genetics	1	0	1	429 (248-601)	
Clinical Haematology	2	1	0	160 (93-189)	
Spinal surgery services	0	1	0	142 (112-164)	
Maxillo-facial surgery	0	0	1	111 (70-133)	
Plastic surgery	1	1	1	93 (68-109)	
Clinical immunology	0	1	0	215 (140-243)	
Interventional radiology	1	0	0	192 (88-260)	
Breast surgery	9	4	5	139 (103-166)	
Tropical medicine	0	1	0	202 (203-203)	
Clinical psychology	1	0	3	177 (116-245)	
Old age psychiatry	0	1	2	108 (108-108)	
Referral to Accident & Emergency	1	0	0	135 (54-166)	
**Community based referrals (total)[3]**	**27**	**19**	**21**		
District nurse	1	3	2	39 (31-43)	PSSRU
Community Podiatrist	4	3	8	42 (35-58)	PSSRU
Community Dietitian	0	2	0	80 (53-96)	DH (2015) National Reference Costs
Smoking cessation (Nurse)	5	3	4	14	15.5 mins nurse time (Curtis 2014)
Healthy lifestyle (Nurse)	0	2	0	14	15.5 mins nurse time (Curtis 2014)
Community Gynaecologist	5	1	0	134 (104-164)	DH (2014) National Reference Costs
Community Physiotherapist	7	4	1	52 (44-58)	(Curtis 2014)
Community Diabetic	1	0	0	69 (38-93)	DH (2015) National Reference Costs
DESMOND diabetes programme	4	0	6	230	Gillett et al (2010) (inflated to 2014)
Expert Patient Programme	0	1	0	302	Richardson et al (2008) (inflated to 2014)
**Primary Care – excludes practice**	**2074**	**1748**	**2094**		

Health service use	Trial arm (Qty)			Unit cost (£) Weighted average (Q1 – Q3)	Source of unit cost
	Control n=323	Post n=312	Nurse n=321		
**visits related to the delivery and participation in intervention (total)[1]**					
GP (11.7mins)	1743	1436	1729	42	(Curtis 2014)
GP nurse (15.5mins)	331	312	365	14	(Curtis 2014)
**A&E visit[4]**	49	36	46	124	DH (2015) National Reference Costs
**Non- Elective hospital admissions (total)[5,6]**	**12**	**4**	**20**		
Biliary acute pancreatitis	0	0	3	2037 (1247-2492)	DH (2015) National Reference Costs
Cardiac catheterisation for coronary artery disease	1	0	1	2643 (1980-3028)	
Chest pain	0	1	0	490 (370-563)	
Abdominal pain	0	0	1	718 (922 -1298)	
Acute ST segment elevation myocardial infarction	2	0	0	1497 (1102-1740)	
Transient ischaemic attack	0	0	1	878 (643-994)	
Guillain-Barre syndrome	0	0	1	1571 (1069-1792)	
Pneumonia	1	0	0	1894 (1406-2238)	
Epilepsy	1	0	0	1125 (788-1266)	
Stroke and cerebrovascular accident	1	0	0	2817 (2018-3396)	
UTI	0	0	1	1530 (1187-1755)	
Detached Retina	0	0	1	908 (303-1935)	
Anxiety states	0	0	1	1393 (984-1628)	
Infective endocarditis in diseases EC, NOS	1	0	0	4480 (2351-5906)	
Acute appendicitis	0	0	1	3017 (2459-3365)	
IUD removed	0	0	1	1780 (1142-2135)	
Ankle fracture	1	0	0	3762 (3109-4271)	
no procedure (NES)	4	3	8	611 (408-726)	
**Elective hospital admissions (total)[5,7]**	**10**	**2**	**3**		
Cardiac catheterisation	2	0	0	2086 (1185-2709)	DH (2015) National Reference Costs
Percut tranlum balloon angioplasty mult coronary	1	0	0	1813 (880-2233)	
Inguinal hernia	0	1	0	2121 (1682-2392)	
Coronary artery bypass graft operations	0	1	0	9310 (7369-9929)	
Laparoscopic cholecystectomy	3	0	0	2567 (2082-2924)	
Endarterectomy of femoral artery NEC	0	0	2	6028 (4593-7209)	
Malignant neoplasm of female breast for chemotherapy	1	0	0	1780 (856-2139)	
Endarterectomy of carotid artery NEC	1	0	0	3911 (2986-4497)	
Neurophysiological operation NOS	2	0	0	1497 (1111-2118)	
Ovarian Cancer	0	0	1	1469 (741-1966)	
Total resource use (All HSU)	2336	1967	2370		

Unit costs are rounded to the nearest whole number and presented in the 2013/14 price year. The health service use presented in this table refers to the base case sample. All the data are based on participant-specific GP records for the trial period with different assumptions and approaches for costing by type of service use:

[1]*Primary care*: GP visits 11.7 minutes; Nurse visits 15·5 minutes;

[2]*Outpatient referrals*: where appropriate, linked to outpatient service descriptions in the reference costs (and reviewed by principal investigator) and a weighted (by throughout) average for consultant/non-consultant led attendances taken; referrals to private sector excluded (n=1);

[3]community referral services costed as referenced; if service use was unclear, an NHS hospital out-patient department was assigned by the principal investigator;

[4]*A&E visit*: as reason for A&E visits was not recorded, an average A&E visit cost for 2013-14 was assigned.

[5]*Hospital admissions*: The principal investigator (blind to study group) reviewed all hospital admissions, and provided either a 'best guess diagnosis/procedure' or listed 'unknown' (n=2). As details on the type of procedure or severity of the symptoms were not available, a weighted (by activity) average of all of the possible scores/procedures was used to derive average cost for elective.

[6]The unit cost for the emergency admissions are a weighted average of the non-elective short stay and non-elective long stay admissions, as the length of stay was unclear.

[7]Hospital admissions without a procedure were treated as non-elective short stay admissions (one day or less). Where hospital admission code was unclear the diagnosis was reviewed by the PI for advice on the nearest appropriate code.

Figure S1: Illustration of pathways within the long-term cost-effectiveness model (Anokye et al 2014a)

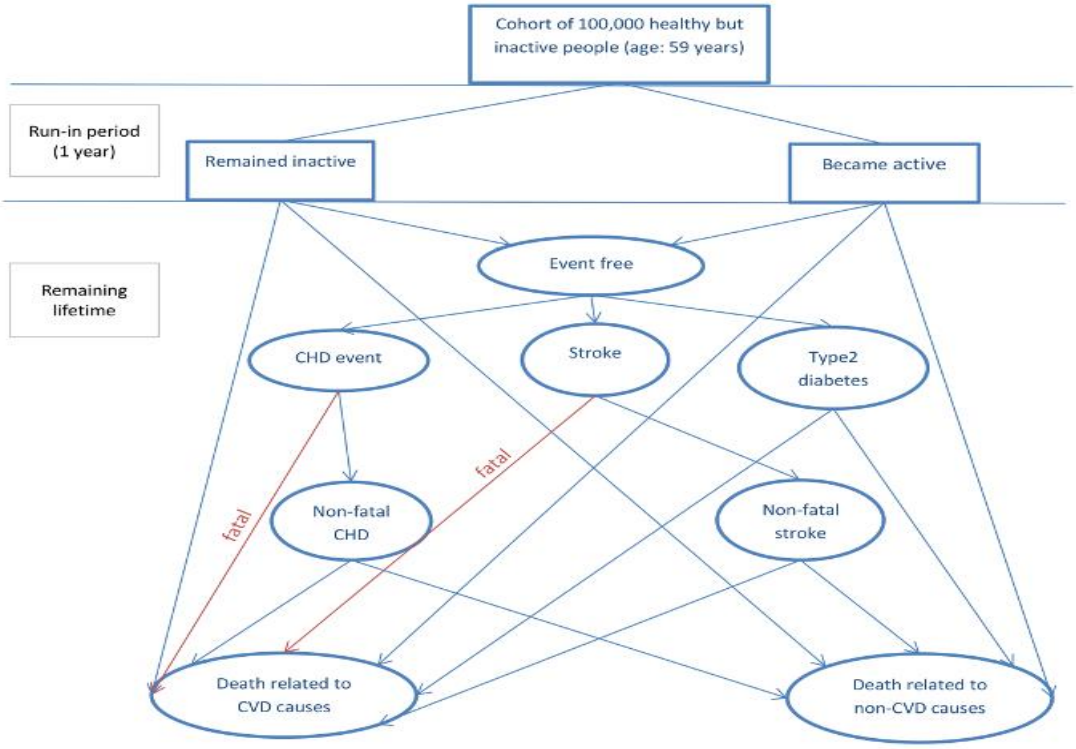

Table S6: Parameter values for long-term cost-effectiveness model

Parameter		Value	Source of data
Relative risks of:			
Becoming active (at year 1)*:	Postal vs control	1·8 (95% CI: 1·4, 2.3)	PACE-UP trial data
	Nurse vs control	1·7 (95% CI: 1·3, 2.2)	
	Nurse vs postal	0·9 (95% CI: 0·7, 1.3)	
Disease (active vs inactive)	CHD	0·90	Hu et al (2007)
	Stroke	0·86	Hu et al (2005)
	Diabetes	0·67	Hu et al (2003)
Non-CVD mortality after:	Non-fatal CHD	1·71	Bronum-Hansen et al (2001)
	Non-fatal Stroke	1·71	
	Diabetes	1·49	Preis et al (2009)
CVD mortality after:	Non-fatal CHD	3·89	Bronum-Hansen et al (2001)
	Non-fatal Stroke	3·89	
	Diabetes	2·61	Preis et al (2009)
CHD fatalities	59-64	11·55%	Ward et al (2005)
	65-74	21·07%	
	75+	14·76%	
Stroke fatalities	55-64	23·28%	Ward et al (2005)
	65-74	23·47%	
	75+	23·42%	
CHD incidence	59-64	0·63%	Ward et al (2005); NCGC (2011)
	65-74	0·97%	
	75+	0·97%	
Stroke incidence	59-64	0·29%	
	65-74	0·69%	
	75+	1·43%	
Diabetes incidence	59	0·06%	Gonzalez et al (2009)
	60-69	0·10%	
	70-79	0·11%	
	80+	0·11%	
Age-specific quality of life	59-64	0·82	Health Survey for England (2011)
	65-74	0·78	
	75+	0·72	
Health state utility weight	Healthy	1·00	Ward et al (2005); NCGC (2011)
	CHD 1st event	0·80	
	post CHD 1st event	0·92	
	Stroke 1st event	0·63	
	post stroke 1st event	0·65	
	Diabetes	0·90	
	Short term psychological benefit of achieving 150 mins of MVPA per week	0·01	PACE UP trial data
Annual costs	Control	£467 (95% CI 365 to569)	PACE UP trial data
	Postal	£376 (95% CI 307 to445)	

Parameter		Value	Source of data
	Nurse	£593 (95% CI 473 to714)	
	CHD 1st event	£4,248	NCGC (2011)
	post CHD 1st event	£485	
	Stroke 1st event	£10,968	
	post stroke 1st event	£2,409	
	Diabetes	£979	

*Relative risks (RR) for achieving at least 150 minutes of MVPA in ≥10 minute bouts at 12 months were estimated from odds ratios (OR) using the formula OR / {(1-P_{ref}) + (P_{ref} *OR)} where P_{ref} is the proportion of all subjects achieving 150 minutes of MVPA in ≥10 minute bouts at baseline i.e. 218/1023 = 0.21. The odds ratios had been derived from a logistic regression model in which the dependent variable, achieving 150 minutes of MVPA in bouts of ≥10 minutes at 12 months, was regressed on baseline minutes of MVPA in bouts of ≥10 minutes, month of baseline accelerometry, day order of wear, day of week, age, gender, general practice and treatment group, with household as a cluster.

Figure S2: Cost-effectiveness plane for postal vs control at 12 months

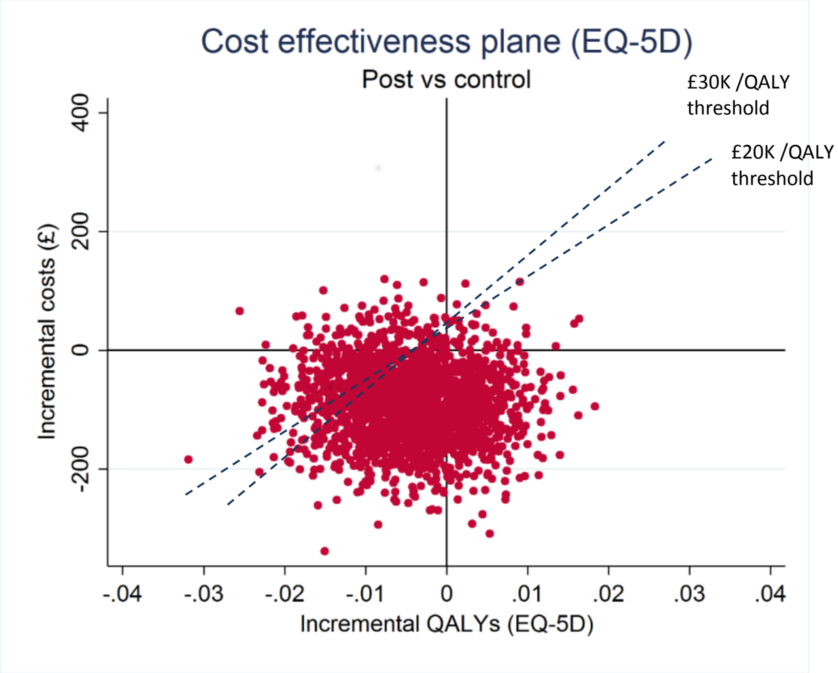

Figure S3: Cost-effectiveness plane for nurse vs control at 12 months

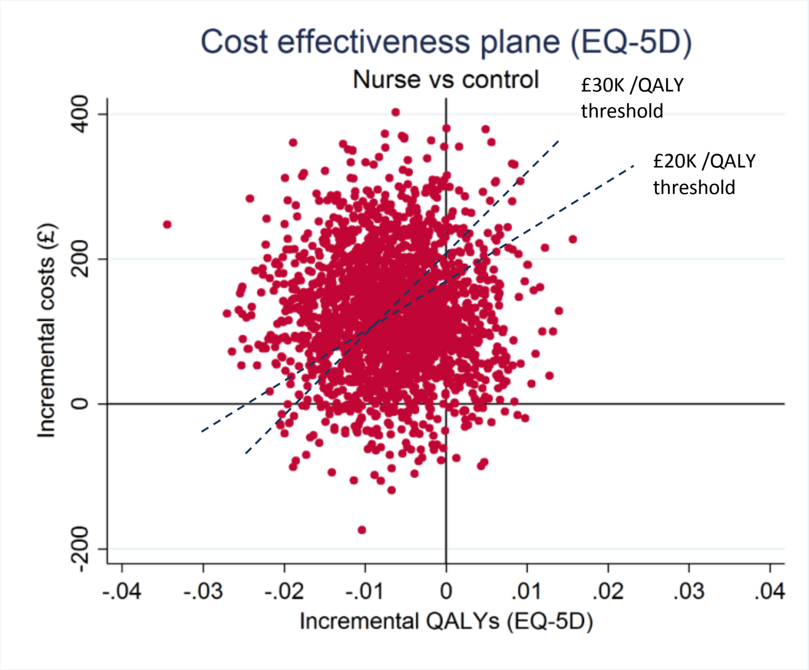

Figure S4: Cost-effectiveness plane for nurse vs post at 12 months

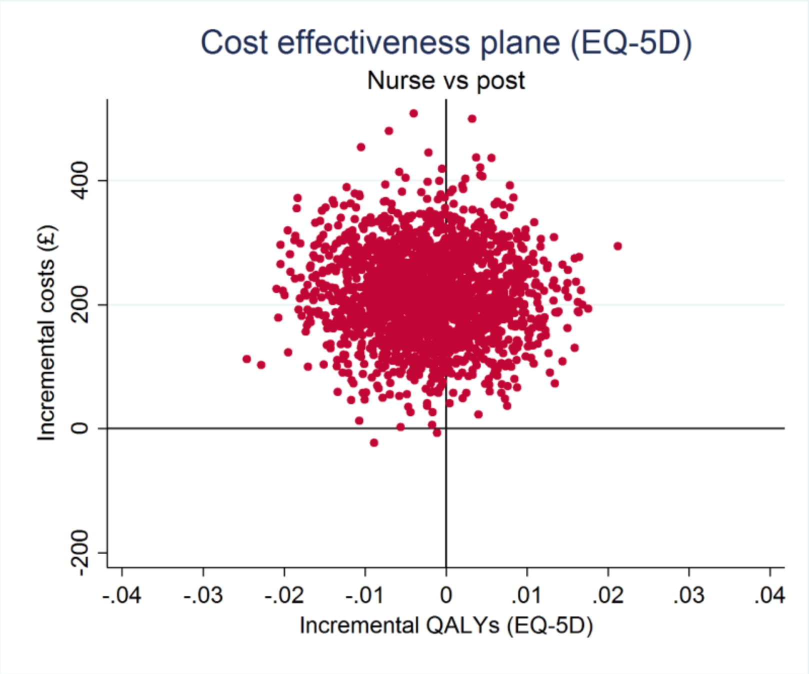

Table S7: Within trial sensitivity analyses (at 12 months)

Parameter	Post vs Control			Nurse vs Control			Nurse vs Post		
	Incremental cost (£)	Incremental QALY	ICER	Incremental cost(£)	Incremental QALY	ICER	Incremental cost (£)	Incremental QALY	ICER
	Mean (95% CI)			Mean (95% CI)			Mean (95% CI)		
Base case	-91[*] (-215, 3)	-0.0043 (-0.0172, 0.0087)	Less costly but less effective than control	126 (-37, 290)	-0.0066 (-0.0201, 0.0068)	Intervention dominated by control	217 (8, 354)	-0.0024 (-0.0156, 0.0109)	Nurse dominated by Post
Whole sample (all randomised)	-40 (-169, -89)	-0.0070 (-0.0195, 0.0054)	Less costly but less effective than control	150 (-6, 306)	-0.0093 (-0.0222, 0.0036)	Intervention dominated by control	190 (48, 332)	-0.0023 (-0.0148, 0.0102)	Nurse dominated by Post
Health service use including only GP data on referrals and admissions	-55 (-166, -56)	-0.0043 (-0.0172, 0.0087)	Less costly but less effective than control	129 (-17, 275)	-0.0066 (-0.020, 0.0068)	Intervention dominated by control	184 (61, 307)	-0.0024 (-0.0156, 0.0109)	Nurse dominated by Post
Health service use including only self-reported serious adverse effects	21 (-65, 107)	-0.0043 (-0.0172, 0.0087)	Intervention dominated by control	144 (65, 224)	-0.0066 (-0.020, 0.0068)	Intervention dominated by control	123 (47, 200)	-0.0024 (-0.0156, 0.0109)	Nurse dominated by Post
Health service use including only GP data on adverse effects	-11 (-107, 85)	-0.0043 (-0.0172, 0.0087)	Less costly but less effective than control	64 (-15, 142)	-0.0066 (-0.020, 0.0068)	Intervention dominated by control	74 (13, 135)	-0.0024 (-0.0156, 0.0109)	Nurse dominated by Post
Excluding all health service use cost	55.2 (55, 55.4)	-0.0043 (-0.0172, 0.0087)	Intervention dominated by control	156.2 (-154, 158)	-0.0066 (-0.0201, 0.0068)	Intervention dominated by control	101 (99, 103)	-0.0024 (-0.0156, 0.0109)	Nurse dominated by Post
Exclusion of missing data**	-91 (-215, 33)	-0.0088 (-0.0231, 0.0055)	Less costly but less effective than control	126 (-37, 290)	-0.0078 (-0.0233, 0.0076)	Intervention dominated by control	217 (8, 354)	0.0009 (-0.0141, 0.0160)	More costly but less effective than control (ICER:£241k)
Changing cost perspective (both participants (all participant costs) and NHS costs)	36 (-177, 250)	-0.0043 (-0.0172, 0.0087)	Intervention dominated by control	107 (-97, 311)	-0.0066 (-0.020, 0.0068)	Intervention dominated by control	71 (-150, 291)	-0.0024 (-0.0156, 0.0109)	Nurse dominated by Post
Changing cost perspective (both participants (part)[3] and NHS costs)	-22 (-235, 191)	-0.0043 (-0.0172, 0.0087)	Less costly but less effective than control	47 (-157, 250)	-0.0066 (-0.020, 0.0068)	Intervention dominated by control	69 (-152, 289)	-0.0024 (-0.0156, 0.0109)	Nurse dominated by Post
Combination of excluding all health service use cost and including all participants costs (minus health service use cost borne by participants)	179 (-1, 361)	-0.0043 (-0.0172, 0.0087)	Intervention dominated by control	153 (24, 281)	-0.0066 (-0.020, 0.0068)	Intervention dominated by control	-27 (-203, 149)	-0.0024 (-0.0156, 0.0109)	Less costly but less effective than control
Pedometer lasts for 1 year (equivalent to pedometers not being re-usable and full	-86 (-210, 38)	-0.0043 (-0.0172,	Less costly but less effective than control	130 (-33, 294)	-0.0066 (-0.0201,	Intervention dominated by	216 (80, 353)	-0.0024 (-0.0156,	Nurse dominated by

[*] This excludes time costs of working out how to use pedometer, diary, and planning to increase work

[**] The pattern of missing data for the base case analysis was multivariate (i.e. some but not all variables had data missing for some participants). The amount of missing data, where observed, was less than 5% except for EQ5D scores (baseline data: 5% (n=51); 3 months data: 7% (n=67); 12 months data: 8%(n=74)).

Parameter	Post vs Control			Nurse vs Control			Nurse vs Post		
	**Incremental cost (£)**	**Incremental QALY**	**ICER**	**Incremental cost(£)**	**Incremental QALY**	**ICER**	**Incremental cost (£)**	**Incremental QALY**	**ICER**
	**Mean (95% CI)**			**Mean (95% CI)**			**Mean (95% CI)**		
Base case	-91* (-215, 3)	-0.0043 (-0.0172, 0.0087)	Less costly but less effective than control	126 (-37, 290)	-0.0066 (-0.0201, 0.0068)	Intervention dominated by control	217 (8, 354)	-0.0024 (-0.0156, 0.0109)	Nurse dominated by Post
cost of pedometer borne in year 1)		0.0087)			0.0068)	control		0.0109)	Post
Pedometer lasts for 4 years (double length of life considered in base case)	-93 (-218, 31)	-0.0043 (-0.0172, 0.0087)	Less costly but less effective than control	124 (-39, 287)	-0.0066 (-0.0201, 0.0068)	Intervention dominated by control	218 (81, 354)	-0.0024 (-0.0156, 0.0109)	Nurse dominated by Post

References

Anokye NK., Lord J., Fox-Rushby J. Is brief advice in primary care a cost-effective way to promote physical activity?, *British Journal of Sports Medicine* 2014; **48 (3)** : 202–206

Brønnum-Hansen H, Davidsen M, Thorvaldsen P. Long-term survival and causes of death after stroke. *Stroke* 2001; **32**: 2131–6.

Curtis LA: Unit costs for health and social care Personal Social Services Research Unit, University of Kent: Canterbury; 2014.

De Cocker K, De Bourdeaudhuij I, Brown W, Cardon G. Moderators and mediators of pedometer use and step count increase in the "10,000 Steps Ghent" intervention. *International Journal of Behavioral Nutrition and Physical Activity* 2009; **6**:3 http://dx.doi.org/10.1186/1479-5868-6-3

Department of Health (2015). NHS Reference Costs 2013 to 2014. Available from:https://www.gov.uk/government/publications/nhs-reference-costs-2013-to-2014

Gillett M, Dallosso HM, Dixon S, et al. Delivering the diabetes education and self- management for ongoing and newly diagnosed (DESMOND) programme for people with newly diagnosed type 2 diabetes: cost effectiveness analysis. *BMJ 2010*: 341:c4093

Gonzalez ELM, Johansson S, Wallander MA. Trends in the prevalence and incidence of diabetes in the UK, 1996–2005. *J Epidemiol Community Health* 2009; **63**:332–6.

Hu G, Jousilahti P, Borodulin K, et al. Occupational, commuting and leisure-time physical activity in relation to coronary heart disease among middle-aged Finnish men and women. *Atherosclerosis* 2007; **194**:490–7

Hu G, Sarti C, Jousilahti P, et al. Leisure time, occupational, and commuting physical activity and the risk of stroke. *Stroke* 2005; **36**:1994–9.

Hu G, Qiao Q, Silventoinen K, et al. Occupational, commuting, and leisure-time physical activity in relation to risk for type 2 diabetes in middle-aged Finnish men and women. *Diabetologia* 2003; **46**:322–29

Health Survey for England, 2011 National Centre for Social Research, University College London-Department of Epidemiology and Public Health. [computer file]. *3rd Edition.* Colchester, Essex: UK Data Archive [distributor], July 2011. SN: 6397

National Clinical Guideline Centre (NCGC). Hypertension: the clinical management of primary hypertension in adults. Clinical guideline: methods, evidence and recommendations. London: National Institute for Health and Clinical Excellence. 2011. Ref Type: Report.

Preis SR, Hwang SJ, Coady S, et al. Trends in all-cause and cardiovascular disease mortality among women and men with and without diabetes mellitus in the Framingham Heart Study, 1950 to 2005. *Circulation* 2009; **119**:1728–35.

Richardson G, Kennedy A, Reeves D, et al. A Cost effectiveness of the Expert Participants Programme (EPP) for participants with chronic conditions. *Journal of Epidemiology and Community Health* 2008; **62**: 361–367

Sharples L, Glover M, Bennett M, Jordan J, Clutterbuck-James A, Davies M, Smith I, Cameron M, Chadwick R, Oscroft N, Morrell M, Fox-Rushby J, Pittman M, Quinnell T (2014) TOMADO: A Crossover Randomised Controlled Trial of Oral Mandibular Advancement Devices for Obstructive Sleep Apnoea-Hypopnoea *Health Technology Assessment 18, 67, http://www.journalslibrary.nihr.ac.uk/hta/volume-18/issue-67#*

Ward S, Jones ML, Pandor A, et al. Statins for the prevention of coronary events. London. National Institute for Clinical Excellence 2005; Ref Type: Report World Health Organisation (2010) Global recommendations on physical activity for health http://www.who.int/dietphysicalactivity/publications/9789241599979/en/
