# Supplementary material for: Short-term and long-term cost-effectiveness of a pedometer-based exercise intervention in primary care: a within-trial analysis and beyond-trial modelling
Source: BMJ Open. 2018 Oct 17;8(10):e021978. doi: 10.1136/bmjopen-2018-021978 (PMC6196874; doi:10.1136/bmjopen-2018-021978)
Supplement: Author's manuscript [file bmjopen-2018-021978.draft_revisions.pdf]

BMJ Open

BMJ Open is committed to open peer review. As part of this commitment we make the peer review history of every article we publish publicly available.

When an article is published we post the peer reviewers' comments and the authors' responses online. We also post the versions of the paper that were used during peer review. These are the versions that the peer review comments apply to.

The versions of the paper that follow are the versions that were submitted during the peer review process. They are not the versions of record or the final published versions. They should not be cited or distributed as the published version of this manuscript.

BMJ Open is an open access journal and the full, final, typeset and author-corrected version of record of the manuscript is available on our site with no access controls, subscription charges or pay-per-view fees (http://bmjopen.bmj.com).

If you have any questions on BMJ Open's open peer review process please email info.bmjopen@bmj.com

The short-term and long-term cost-effectiveness of a pedometer-based exercise intervention in primary care: A within-trial analysis and beyond-trial modelling

Journal:	*BMJ Open*
Manuscript ID	bmjopen-2018-021978
Article Type:	Research
Date Submitted by the Author:	09-Feb-2018

Complete List of Authors:	Anokye, Nana; Brunel University, Health Economics Research Group<br>Fox-Rushby, Julia; Kings College London, School Population Health and Environmental Sciences<br>Sanghera, Sabina ; University of Bristol, School of Social and Community Medicine<br>Cook, Derek; St George's University, Division of Population Health Sciences<br>Limb, Elizabeth; St George's University, Division of Population Health Sciences<br>Furness, Cheryl; St George's University, Division of Population Health Sciences<br>Kerry, Sally; Queen Mary University of London<br>Victor, Christina; Brunel University, Social Work & Community/Public Health<br>Iliffe, Steve; University College London, Primary Care and Population Sciences<br>Ussher, Michael; St George's University of London, Division of Population Health Sciences and Education<br>Whincup, Peter; St Georges, University of London<br>Ekelund, Ulf; Norwegian School of Sport Sciences<br>DeWilde, Stephen; St George's University of London<br>Harris, Tess; St George's University of London

Keywords:	HEALTH ECONOMICS, PUBLIC HEALTH, physical activity, cost-effectiveness, RCT, Long term modelling

SCHOLARONE™
Manuscripts

Full Title: The short-term and long-term cost-effectiveness of a pedometer-based exercise intervention in primary care: A within-trial analysis and beyond-trial modelling

Short title: Economic evaluation of pedometers delivered through primary care

Nana Anokye[1] PhD, JuliaFox-Rushby[2] PhD, SabinaSanghera[3] PhD, Derek G. Cook[4] PhD, Elizabeth Limb MSc[4], Cheryl Furness MSc[4], Sally Kerry PhD[5], Christina Victor PhD[6], Steve Iliffe FRCGP[7], Michael Ussher PhD[4], Peter H.Whincup PhD[4], Ulf Ekelund PhD[8,9], Steve DeWilde MD[4], Tess Harris MD[4]

[1]Health Economics Research Group, Brunel University London, Kingston Lane, Uxbridge, UB8 3PH, UK;

[2]Department of Population Health Sciences, Guys Campus, Kings College London, SE1 1UL

[3]School of Social & Community Medicine, University of Bristol, BS8 1TH

[4]Population Health Research Institute, St George's University of London, Cranmer Terrace, London, SW17 0RE, UK;

[5]Pragmatic Clinical Trials Unit, Queen Mary's University of London, 4 Newark Street, London, E1 2AT, UK;

[6]Department of Clinical Sciences, Brunel University London, Kingston Lane, Uxbridge, UB8 3PH, UK;

[7]Research Department of Primary Care & Population Health, University College London, St Dunstans Road, London, W6 6RP, UK;

[8]Department of Sport Medicine, Norwegian School of Sport Sciences, Postbox 5 Torshov, 0412 Oslo, Norway;

[9]MRC Epidemiology Unit, University of Cambridge, Cambridge Biomedical Campus, Cambridge, CB2 0QQ, UK;

Corresponding author

Professor Julia Fox-Rushby, Department of Population Health Sciences, Faculty of Medicine and Life Sciences, 5th Floor Addison House, Guys Campus, Kings College London, SE1 1UL

Email: Julia.Fox-Rushby@kcl.ac.uk Telephone 020 7848 8672

Orcid id: 0000-0003-0748-0871

For peer review only - http://bmjopen.bmj.com/site/about/guidelines.xhtml

Abstract

Objectives: A short- and long-term cost-effectiveness analysis (CEA) of two pedometer-based walking interventions compared with usual care

Design: a) Short-term CEA: parallel three-arm cluster randomised trial randomised by household b) Long-term CEA: Markov decision-model

Setting: Seven primary care practices in South London, United Kingdom

Participants: a) Short-term CEA: 1023 people (922 households) aged 45±75yrs without physical activity (PA) contraindications b) Long-term CEA: 100,000 cohort aged 59-88yrs

Interventions: Pedometers, 12-wk walking programmes, and PA diaries delivered by post or through three PA consultations with practice nurses

Primary and Secondary Outcome Measures: Accelerometry-measured change (baseline-12months) in average daily step-count and time in 10-min bouts of moderate-vigorous PA, and EQ5D5L quality-adjusted life-years (QALYs)

Methods: Resource use costs (£2013/4) from an NHS perspective, presented as incremental cost-effectiveness ratios for each outcome over a 1-year and life-time horizon, with cost-effectiveness acceptability curves and willingness to pay per QALY. Deterministic and probabilistic sensitivity analyses evaluate uncertainty.

Results: a) Short-term CEA: At 12months, incremental cost/step was 19p(£6) and £3.61(£109) per minute in ≥10 minute MVPA bouts for nurse-support compared with control (postal group). At £20,000/QALY, the postal group had a 50% chance of being cost-saving compared with control. b) Long-term CEA: The postal group had more QALYs (+759QALYs, 95% CI 400, 1247) and lower costs (-£11m, 95% CI -12,-10), than control and nurse groups, resulting in an incremental net monetary benefit of £26m per 100,000 population. Results were sensitive to reporting serious adverse events, excluding health service use, and including all participant costs.

Conclusions: Postal delivery of a pedometer intervention in primary care is cost-effective long-term and has a 50% chance of being cost-effective, through resource savings, within one year. Further research should ascertain maintenance of the higher levels of PA, and its impact on quality of life and health service use.

Trial Registration: ISRCTN98538934

For peer review only

For peer review only - http://bmjopen.bmj.com/site/about/guidelines.xhtml

Strengths and Limitations of this study

- This study provides the first primary data on the short-term costs associated with delivering pedometers to a large (n=1023), population-based, sample from primary care alongside a high quality randomised controlled trial that achieved a 93% follow-up rate at 12 months.
- Results from the trial are fed into a peer-reviewed, policy-relevant, Markov model to estimate long-term cost-effectiveness as trials of public health interventions are unable to reflect the balance of costs and effects when benefits occur in the long term.
- Results are tested in a number of sensitivity analyses to assess the impact of changing perspective, missing data, and of taking more conservative accounting of outcomes and cost impact.
- The main limitation of the economic analysis is the lack of information about the likelihood of maintaining PA over the long term and the exclusion of long term impacts on other conditions e.g. cancers

For peer review only

Introduction

Increasing physical activity (PA) is a widely-stated policy aim from local to international level.[1,2] Walking is a safe and, potentially cheap, activity that has the potential to reduce cardiovascular disease, diabetes, cancer and poor mental health.[3] It is therefore important to establish which approaches are effective at: encouraging inactive people to do at least some walking; increasing the number of people walking briskly for at least 150 mins a week (ie achieving moderate-to-vigorous PA (MVPA) guidelines[2]); and/or maintaining increases in walking over time. This would also provide the basis for estimating cost-effectiveness and supporting recommendations for policy and practice.

Until recently, the best evidence of pedometer-based walking programmes was from systematic reviews that relied on small, short-term, studies where the independence of pedometer effects, from other support provided was unclear.[4] These had shown that walking interventions can achieve increases of ~2000-2500 steps/day at 3 months, but often relied on volunteer samples or high risk groups and did not assess time in MVPA, as defined in PA guidelines, as an outcome. New evidence from a large, randomised, trial clustered by household (PACE-UP) compared delivery of pedometers by post or through primary care nurse-supported PA consultations, among 1,023 inactive primary care patients aged 45-75 years from seven practices in south London. The results showed that step-counts increased by around 10% and time in MVPA in 10-minute bouts by around a third, with both the nurse and postal delivery arms achieving similar 12-month outcomes.[4] This is important because primary care is a key context for PA interventions as it facilitates direct reach into the community and continuity of care with practice nurse involvement. It is shows that this type of intervention is suitable for older adults, where exercise referral schemes have been disappointing[4].

Other than a small, highly selected, study which limited outcomes to steps achieved among 79 people from one family physician practice in Glasgow,[5] there is no primary evidence of the cost-effectiveness of pedometer programmes in the UK. Elsewhere, in Australia, New Zealand, and the Netherlands, economic models from community-based adults with low PA levels compare pedometer prescriptions and pedometer-based telephone coaching with usual practice.[6–8] These indicate, pedometer-based interventions may be cost-effective in the long term, but estimates vary widely and generalisability is not considered.[9]

For peer review only - http://bmjopen.bmj.com/site/about/guidelines.xhtml

The analytic horizon of cost-effectiveness analyses should extend far enough into the future to capture all benefits and harms, although in practice this can be limited by the amount and quality of data.[10] NICE's public health guidance[11] also recommends providing results that reflect the short term (one to three years). This is reinforced in NICE's return on investment models,[12] which argue that shorter-term decision-making is of key interest to some decision-makers and which have been used by commissioners.

This paper estimates the short-term (one year) and long-term (life-time) cost-effectiveness of pedometers delivered by post or through practice nurse consultation for 1,023 inactive adults aged 45-75years. The short-term evaluation arises from a within-trial analysis of individual resource use and costs of interventions provided in the PACE-UP trial.[4] The cost and effectiveness results from the trial are used to populate a long-term model[13] for life-time cost-effectiveness.

Methods

Short-term cost-effectiveness

The short-term within-trial cost-effectiveness analysis was conducted alongside the PACE-UP trial[4,14] that evaluated wo intervention groups against control. The two intervention groups received pedometers (SW-200 Yamax Digi-Walker) (one by post), patient handbook; PA diary (including individual 12-wk walking plan), with the nurse group also offered three individually tailored practice nurse PA (10- to 20-min) consultations (nurse-support group only) at approximately weeks 1, 5, and 9. [4] The control group followed usual practice and were not provided with any feedback on their PA levels or materials promoting PA during the trial.[4] These interventions could therefore evaluate the incremental effect of adding nurse support to pedometers.

The costs for the two intervention arms include set-up costs, staff training and intervention delivery (including; pedometers & clips, batteries, handbooks, diaries, postage, nurse time, time making appointments). Measures of each resource use were taken from administrative/trial management records, computer-based diaries, and interviews with the trial manager and principal investigator. To account for potential changes in falls, change in use of health services following differential contact of health services by participants or unintended resources consequences, general health service use (eg general (family) physician visits, hospital admissions, accident and emergency attendances, referrals) was collected at participant level, through a one-time download of physician

For peer review only - http://bmjopen.bmj.com/site/about/guidelines.xhtml

records at the end of the trial, and linked to procedure codes using PI judgement (blind to treatment group) to facilitate costing across elective and non-elective admissions. Information on costs borne by patients (eg time use, out of pocket expenses associated with walking groups, plus any related travel costs) were collected by questionnaire at 3 and 12 months. Resources were valued using national tariffs where possible[15,16] to increase generalisability; where not available tariffs from St Georges Hospital, London, were used. All costs are expressed in £2013-2014 sterling, inflated to this base year where appropriate using the Hospital & Community Health Service inflation index. As the trial lasted for one year, a discount rate was not applied. (See Supplementary File Tables S1-S5)

Outcomes were; (a) changes in daily steps and weekly minutes of MVPA in bouts of ≥10 mins, based on objectively measured PA by accelerometry and (b) changes in Quality Adjusted Life Years (QALYs), based on participant completion of the EQ-5D-5L questionnaires at baseline, 3 and 12 months. Utility weights were assigned using the 'crosswalk' function[17] linked to the standard UK-based weights[18], with QALYs based on the area under the curve.

Patterns of missing data were investigated, with multiple imputation by chained equations fitted to replace item non-response. Missing EQ-5D data were replaced using an index rather than domain imputation as recommended[19]. Mean imputation was used where missing data was ≤5%[20].

Results are reported, from an NHS perspective, as incremental cost-effectiveness ratios for cost per change in daily steps and cost per QALY for a one-year time-period, adjusted for baseline differences. A generalised linear model was fitted separately for costs and QALYs with clustered standard errors. To provide more precise estimates of uncertainty, the 'margins method' was used to generate sample means by trial arm for costs and QALYs[21]. Cost models were fitted using the Poisson distribution and QALY models using the binomial 1 family, equivalent to beta regression[22]. The choice of distributional family for the models was based on the modified Park test and comparison of observed and predicted values. Covariates included baseline level (for the QALY-based models)[21], practice and variables found to be correlates of PA-related outcomes[23]- ie demography (age, gender, ethnicity, marital status, education, employment, socio economic status, cohabitation), health (number of disease conditions), and other lifestyle behaviours (smoking and alcohol intake). Reduced models

For peer review only - http://bmjopen.bmj.com/site/about/guidelines.xhtml

were generated using Wald tests to examine the joint significance of variables found not to be significant (at 5%) in the base model.

Deterministic sensitivity analyses assessed: (a) inclusion of all randomised patients (rather than only those who provided accelerometry data); (b) exclusion of costs of general health service use beyond immediate intervention; (c) methods of accounting for adverse events; (d) perspective of analysis (ie including all and parts of participant costs); (e) varying the length of life of a pedometer; (f) the combination of excluding all health service use costs, and (g) including participant costs related to participation in physical activity and the interventions (minus health service use cost borne by participants, to ensure consistency in perspective). To reflect stochastic uncertainty surrounding mean incremental cost-effectiveness, cost-effectiveness planes (CEPs) and acceptability curves (CEACs) were constructed using 2000 non-parametric bootstrap samples from the base case estimates.

Long-term cost-effectiveness

A Markov model used to support NICE public health guidance[24] and return on investment modelling[12] was adapted to examine the long-term (life-time) cost effectiveness. From an NHS perspective, costs (2013/4 prices) and health outcomes from reduced disease, expressed as QALYs were discounted at the rate of 3.5% per annum. Results are reported as incremental cost-effectiveness ratios, cost-effectiveness acceptability curves and incremental net benefit statistics.

In the original model,[13] a cohort of 100,000 33 year-old people were followed in annual cycles over their life-time. At the end of the first year of the model, the cohort is either ‘active’ (doing 150 minutes of MVPA in 10 mins bouts per week) or ‘inactive’ and they could have one of 3 events (non-fatal CHD, non-fatal stroke, type 2 diabetes), remain event free (ie without CHD, stroke, or diabetes) or die either from CVD or non-CVD causes, each of which had assigned annual treatment costs (split by initial event and follow-up). After the first year, people would revert to PA patterns observed in long-term cohort studies on the relationship between PA and disease conditions[13]. Active individuals had lower risks of developing CHD, stroke and type-2 diabetes. People who become active in the first year (irrespective of trial arm) also accrue short-term psychological benefits, a one-off utility gain associated with achieving the recommended level of physical activity[13] (see supplementary file Figure S1).

For peer review only - http://bmjopen.bmj.com/site/about/guidelines.xhtml

The model was adapted, using data from the PACE-UP trial, in the following ways:

a) a cohort of 100,000 people aged 59 years followed, in annual cycles, to 88 years, reflecting the average age of all trial participants at baseline and the average life expectancy for people aged 59 years in UK[25] and exposed, at this age, to interventions (either nurse or postal) in an unexposed population ie control group/usual care;

(b) age-specific estimates were revised to reflect the change in the cohort age,

(c) the within-trial cost of interventions was used, with a second year of annuitized values included appropriately - postal (£5·03/person) and nurse group (£4·14/ person);

(d) effectiveness reflected as the relative risk of achieving ≥150 MVPA mins per week in ≥10 minute bouts; and

(e) short-term psychological benefits of PA (one-off utility gain) estimated using beta regression fitted for EQ-5D scores at 12 months for active people controlling for EQ-5D scores at baseline, demographics, practice, disability and trial arm using.

All other parameters remained the same as the original model, based on literature reviews or evidence from national/international science-based guidance on PA and health. Parameter estimates are provided in supplementary file Table S7.

Deterministic sensitivity analysis explored two alternate, conservative, scenarios: (1) Exclusion of all health service use cost consequences during trial period (model year one) and assumed no psychological benefits in the first year of being physically active. This was considered due to the uncertainty around short term changes to health service use and because previous studies found the exclusion of short-term QALY gain associated with being physically active to affect conclusions[13]; (2) Scenario 1 plus all patient costs related to participation in physical activity and the interventions. This most conservative combination represented a 'worst case' scenario in the trial. Probabilistic sensitivity analysis was based on 10,000 Monte Carlo simulations and included all parameters except baseline mortality, as the mortality census data has little uncertainty.

Results

Short-term cost-effectiveness

Table 1 summarises data on costs, EQ-5D-5L utility scores and QALYs by trial arm. At 3 months, average cost per participant was highest in the nurse group (£249) followed by the postal (£122) and control group (£107).

For peer review only

The cost of nurse-supported pedometer delivery was seven times greater (£50) than the postal group (£7), and set-up double. The mean and distribution of cost is affected considerably by inclusion of health service use. This resulted in the control group costing £35 more per participant than the postal group and £12 more than the nurse group. Results are similar at 12 months, except for the control arm, which has a higher overall average cost than the postal arm.

Table 2 shows that, at three months, mean incremental costs were significantly higher for the nurse group compared with the postal (+£120, 95% CI £95, £146) and control groups (+£135, 95% CI £99, £171) but not statistically significantly higher for the postal compared with control group. While increases in both daily steps and weekly minutes of MVPA in ≥10 minute bouts for both interventions compared with control, and for the nurse group compared with postal (nurse: +481steps (95% CI: 153, 809), +18mins MVPA (95% CI: 1, 35)) were statistically significant, the small mean decrease in QALYs is not statistically significant for any comparison. The cost per additional minute of MVPA was 35p for postal group and £2·21 for the nurse group and therefore the (slightly) fewer QALYs for both interventions compared with control contributed to the dominance of each intervention by the control group (ie the control group cost less and had more QALYs). To move from a postal to nurse delivered pedometer would cost 25p per additional step and £6·67 per additional MVPA minute. However, in terms of cost-effectiveness, the nurse group costs more and produces less QALYs on average than the postal group at 3 months.

Results differ at 12 months. Compared with the control group, the postal arm cost less on average (-£91) and the nurse group more (+£126) but neither are statistically significant. The increase in cost of moving from a postal to nurse delivery is also statistically significantly higher (+£217, CI £81, £354). While both interventions are associated with a statistically significant increase in steps and weekly mins of MVPA, the difference between intervention groups is not statistically significant at 12 months. The small decrements in QALYs at each incremental comparison are not statistically different. The postal group took more steps (+642) and cost less on average (-£91) compared with control and dominates control in terms of PA outcomes. The nurse group cost 19p per additional step and £3.61 per additional minute of MVPA compared with control, with this rising to £6 and £109 respectively when compared with the postal group. In terms of QALYs, the nurse group is still dominated (ie cost more and had worse outcomes) by the control and postal groups. However, on average, each

For peer review only - http://bmjopen.bmj.com/site/about/guidelines.xhtml

For peer review only

QALY lost in the postal group compared with control is associated with a saving of £21,162, which could therefore be considered cost-effective.

The probabilistic sensitivity analyses broadly confirm the findings of the base case; the postal group is most often associated with lower QALYs along with cost savings and the nurse group tends to have both lower QALYs and higher costs compared with control and postal group (Supplementary file, Figs S2-S4). Figure 1 shows that at £20,000 per QALY gained/lost, the postal group has a 50% chance of being cost-effective compared with control (usual care). This falls to 42% at £30,000/QALY, which reflects the postal group having most observations in the lower left hand quadrant (as seen in Supplementary file, Fig S2). Figure 1 also shows that, at a willingness to pay/lose a QALY of £20,000, the nurse group has a 5.5% chance of being cost-effective compared with control.

The deterministic sensitivity analyses (Supplementary File, Table S7) mostly produced results consistent with the base case findings. However, in four circumstances, usual care would dominate both the postal and nurse groups at 12 months; i) using health service use based on self-reported serious adverse effects; ii) excluding all health service costs; iii) changing perspective (including all participant costs); and iv) the worst-case 'combined scenario' sensitivity analyses..

Long-term cost-effectiveness

Table 3 shows that, over the remaining life-time from age 59, the nurse group would be costlier (£11m, 95% CI: £10m, £12m) but have more QALYs (671 95% CI: 346, 1071) per 100,000 population than the control group and therefore provide each additional QALY at a cost of £16,368. However, the postal group would have lower life-time costs than the control arm (-£11m per 100,000 population, 95% CI: £-12m, £-10m) and more QALYs (759, CI: 400, 1247) it is therefore the dominant option, with an incremental net benefit of £26million per 100,000 population (95% CI: £18m, £36m). These results are confirmed by the incremental net benefit, which shows the £2m per 100,000 for nurse group compared with control is not significantly different and compared with the post group is significantly negative (-£24m 95% CI: -£27, -£21).

For peer review only - http://bmjopen.bmj.com/site/about/guidelines.xhtml

The stochastic uncertainty associated with the mean incremental cost-effectiveness ratio (ICER) (Figure 2) indicates the above findings are robust. There is a 100% likelihood, at a willingness to pay of £20,000/QALY, that the postal group is cost-effective compared with the control and nurse groups. This is consistent with the estimates of net monetary benefit in Table 3. At £20,000/QALY, there is a 70% likelihood that the nurse group would be cost-effective compared with control (Figure 2).

The results for scenario 1 of the sensitivity analyses were: (i) Postal vs control: postal moved from a dominant position to a more expensive option (+£4m) with more QALY gains (+609QALYs), and an ICER of £6,100;(ii) Nurse vs control: The ICER increased from £16,000 to £26,000 (+£14m, +538QALYs); (iii) Nurse vs postal: The Nurse group remained dominated by postal group (+£10m, -87QALYs).For scenario 2, the sensitivity analyses showed: (i) postal vs control: postal moved from a dominant position to more expensive (+£16m) and more QALY gains (+609 QALYs) with an ICER of £26,600; (ii) Nurse vs control: The ICER increased from £16,000 to £25,400 (+£13.7m, +538QALYs); (iii) Nurse vs postal: Nurse moved from dominated position (where costs are higher and QALYs lower to a cost-effective position (where both costs and QALYs are lower) (-£2m, -87QALYs).

Discussion

The life-time cost-effectiveness of posting a pedometer with written instructions to a cohort of 100,000 insufficiently active people aged 59 years (who have indicated an interest in research or participation in walking) would cost less (-£11m, 95%CI -12,-10) and provide more QALYs (759 QALYs, 95%CI 400, 1247) than usual care. Most cost-savings and quality of life benefits derive from reductions in stroke, CHD and type-2 diabetes. This finding was robust (incremental net benefit of £26m, 95%CI £18m, £36m) and sensitivity analyses showed that even excluding short-term cost savings would not change the conclusion that the postal group would be extremely cost-effective in the long-term (ICER: £6,100/QALY). Sending a pedometer by post with instructions from a primary care provider to inactive people aged 45-75 also has a 50% chance of being cost-effective *within a year*, as a 1 QALY loss was associated with saving over £21,000. The nurse group had higher costs and lower QALYs than both control and postal groups at 1 year. While sensitivity analyses did not change conclusions in most cases, in three cases (using self-reported serious adverse events, excluding health

For peer review only - http://bmjopen.bmj.com/site/about/guidelines.xhtml

service use, including all participant costs) it did, indicating that the control group would dominate (ie have lower costs and more QALYs) than both the postal and nurse groups.

A key strength of this study is the base of individualised cost and effectiveness data on a large, population-based, cluster-randomised, controlled trial with excellent follow-up data to one year (93.4%, Harris et al 2017)[4], designed to produce generalisable results, for cost per QALY estimates at one year and as inputs to a long-term model of cost-effectiveness. It is also the only study to have included provider and user perspectives, extended commonly used techniques to account for clustering and used conservative assumptions for both short- and long-term sensitivity analyses.

One weakness of the within-trial cost-effectiveness study concerns the use of PI judgement to determine costs of admissions, and therefore alternative assumptions were explored in sensitivity analyses. Patient reported cost data were collected for months 1-3 and 9-12, with the last 3 months multiplied to represent costs across all months from 4-12. If significantly underestimated, this could be decisional. To date, there are no primary economic data beyond 12 months of an intervention and very few trials include measures of quality of life measures alongside PA. Therefore, with respect to the long-term modelling, a key gap in knowledge is the likelihood of maintaining PA beyond 12 months. This model assumes differences in PA at 1 year in the trial relate to the same long-term benefit associated with the same difference in cohort studies, but this could be updated once longer-term follow-up data become available. Other challenges set out in (Anokye et al 2014)[13] are relevant here eg cancer and adverse events are not accounted for, which could lead to over or under-estimation of cost-effectiveness.

This study feeds into an area with very limited primary data[26,27] populated only by small studies[5,6]. In New Zealand pedometers were shown to have a 95% probability of being a cost-effective addition to green prescriptions at 12 months[5], much higher than the 50% likelihood we found. Other models of long-term cost-effectiveness studies identified cost savings and improved quality of life at a population level from pedometers in the long term[8,28] or indicated high probabilities of long-term cost-[7,29]. Guidance has also suggested that long-term monitoring/support at £25/year would be very cost-effective. Our study provides further support that pedometer-based programmes are a cost-effective method of improving health-related quality of life in both the

For peer review only - http://bmjopen.bmj.com/site/about/guidelines.xhtml

short and long-term. Assumptions about intervention effectiveness beyond one year has mixed impacts, and further research is required to better judge whether existing models over- or under-predict cost-effectiveness.

Current public health guidance from NICE on pedometers[30] advises using pedometers as "part of a package which includes support to set realistic goals in one to one meetings (whereby the number of steps taken is gradually increased), monitoring and feedback. Our results not only provide substantially better economic data for use by NICE but also suggest guidance should be updated to reflect the value of providing pedometers, to people who have made some form of commitment (ie to a trial), through the post. For those practices that have implemented consultation-based distribution of pedometers, moving to postal delivery could save costs within a year, with similar outcomes.

Postal delivery of pedometer interventions to inactive people aged 45-75 through primary care is cost-effective in the long-term and has a 50% chance of being cost-effective, through resource savings, within one year. Further research is needed to ascertain the extent to which higher PA levels are maintained beyond one year and the impact of PA on quality of life and general health service use in both the short and long-term.

For peer review only - http://bmjopen.bmj.com/site/about/guidelines.xhtml

Supporting Information

Supplementary file 1: Copy of open access protocol for trial

Supplementary file 2: Figures 1 & 2

Supplementary file 3: Document for online supplement giving fuller study details

Supplementary file 4: CHEERS reporting statement

Author Contributions

Contributors: JFR conceived of the economic analysis, was co-applicant for funding, jointly designed the economic data collections tools, wrote the economic analysis plan, collected part of the data, supervised the economics, and jointly drafted and amended the script. She is guarantor for this script. NA jointly designed the data collection tools, cleaned and analysed the economic data, and jointly drafted and amended the script. SS collated and analysed the hospital cost data, commented on drafts and reviewed the final script. DC, EL and SK designed data collection for and analysed the intermediate outcome data underpinning the economic analyses, discussed plans and results as presented through the trial, commented on drafts of this manuscript and reviewed the final script. CF collated and provided access to the administrative data used for the economic analysis, was the research project administrator, commented on drafts and reviewd the final script. TH was the principal investigator, involved at all points of the planning, progress and review of the economic evaluation including commenting on drafts and review of the final script. She is guarantor for the whole trial. CRV, PHW, MU, SI, UE,SdW all conceived the trial plan and applied for funding, they contributed to conceptualisation of the economics within the broader context of the trial, discussed plans and results as presented through the trial, commented on drafts of this manuscript and revised the final script.

Acknowledgements

We thank Iain Carey for help with processing the primary care data as well as Abbie Hill and Julie Whittaker who ably supported administration of the economics project at Brunel University London. We remember and

For peer review only

For peer review only - http://bmjopen.bmj.com/site/about/guidelines.xhtml

thank too, our colleague and co-investigator, Dr S M Shah who died in September, 2015. We would like to thank the South-West London (UK) general practices, their practice nurses who supported this study, and all the patients from these practices who participated: Upper Tooting Road Practice, Tooting; Chatfield Practice, Battersea; Wrythe Green Practice, Carshalton; Francis Grove Practice, Wimbledon; Putneymead Practice, Putney; Heathfield Practice, Putney; and Cricket Green Practice, Mitcham. We would also like to thank our supportive Trial Steering Committee: Professor Sarah Lewis (chair); Professor Paul Little (GP representative); Mr Bob Laventure (Patient and Public Involvement representative).

For peer review only

For peer review only - http://bmjopen.bmj.com/site/about/guidelines.xhtml

Funding Statement

This research was supported by the Health Technology Assessment (HTA) Programme, National Institute for Health Research (project number HTA 10/32/02 ISRCTN42122561), The funding organisation played no role in the study design, collection, analysis and interpretation of the data, writing or decision to submit the paper for publication The views and opinions expressed therein are those of the authors and do not necessarily reflect those of the Health Technology Assessment (HTA) Programme, National Institute for Health Research, National Health Service or the Department of Health.

Competing Interests

JFR reports Board Membership of the Public Health Research Funding Board of NIHR. TH sits on Primary Care Community Interventions Panel for the Health Technology Assessment Programme of NIHR. All authors have been contracted to evaluate other public health interventions, including pedometers, for the NIHR.

Patient Consent

No individual can be identified from the work presented in this paper.

Data Sharing

Data is available upon request from Dr Tess Harris

For peer review only

References

1. Lichfield District Council (2016) Physical Activity and Sport Strategy 2016-2020 Available from: https://www.lichfielddc.gov.uk/Residents/Leisure-and-culture/Leisure-centres/Downloads/Lichfield-District-Physical-Activity-and-Sport-Strategy.pdf. (downloaded 21.02.201)

2. World Health Organisation (2010) Global recommendations on physical activity for health http://www.who.int/dietphysicalactivity/publications/9789241599979/en/

3. Kyu H, Bachman V, Alexander L, Mumford J, Afshin A, Estep K, Veerman J, et al. Physical activity and risk of breast cancer, colon cancer, diabetes, ischemic heart disease, and ischemic stroke events: systematic review and dose-response meta-analysis for the Global Burden of Disease Study 2013BMJ 2016;354:i3857

4. Harris T, Kerry SM, Limb ES, Victor CR, Iliffe S, Ussher M et al. Effect of a Primary Care Walking Intervention with and without Nurse Support on Physical Activity Levels in 45- to 75-Year-Olds:The Pedometer And Consultation Evaluation (PACE-UP) Cluster Randomised Clinical Trial. Plos Med 2017 http://dx.doi.org/10.1371/journal.pmed.1002210

5. Shaw R, Fenwick E, Baker G, McAdam C, Fitzsimons C, Mutrie N. Pedometers cost buttons: the feasibility of implementing a pedometer based walking programme within the community. *BMC Public Health* 2011, **11**:200

6. Leung W, Ashton T, Kolt G, Schofield G, Garrett N, Kerse N, et al.Cost-effectiveness of pedometer-based versus time-based Green Prescriptions: the Healthy Steps Study Australian *Journal of Primary Health*, 2012, **18**: 204–211

7. Over E, Wendel-Vos G, van den Berg M, Hamberg-van Reenen H, Tariq L, Hoogenveen R, van Baal P. Cost-effectiveness of counseling and pedometer use to increase physical activity in the Netherlands: a modeling study. Cost Effectiveness and Resource Allocation, 10:13

8. Cobiac LJ, Vos T, Barendregt JJ. Cost-effectiveness of interventions to promote physical activity: a modelling study. *PLOS medicine* 2009 https://doi.org/10.1371/journal.pmed.1000110

9. Vijay G, Wilson E, Suhrcke M, Hardeman W, Sutton S, on behalf of the VBI Programme Team . Are brief interventions to increase physical activity cost-effective? A systematic review Br J *Sports Med* 2015; **0**:1–11

10. Honeycutt A, Clayton L, Khavjou O, Finkelstein E, Prabhu M, Blitstein J et al. Guide to Analyzing the Cost-Effectiveness of Community Public Health Prevention Approaches Report to U.S. Department of Health and Human Services RTI Project Number 0208827.001. 2006. Available from: https://aspe.hhs.gov/system/files/pdf/74686/report.pdf

11. National Institute for Health and Care Excellence (2014) Developing NICE guidelines: the manual. Available from: https://www.nice.org.uk/process/pmg20/chapter/incorporating-economic-evaluation

12. Matrix. Estimating Return on Investment for interventions and strategies to increase physical activity London: Matrix Knowledge, 2014.

13. Anokye, NK., Lord, J., Fox-Rushby, J., Is brief advice in primary care a cost-effective way to promote physical activity?, *British Journal of Sports Medicine* 2014, **48 (3)** : 202–206

14. Harris T, Kerry SM, Victor CR, Shah SM, Iliffe S, Ussher M, Ekelund U et al. PACE-UP (Pedometer and consultation evaluation - UP) - a pedometer-based walking intervention with and without practice nurse support in primary care patients aged 45-75 years: study protocol for a randomised controlled trial. Trials 2013;**14**:418.

15. Department of Health (2015). NHS Reference Costs 2013 to 2014. Available from:https://www.gov.uk/government/publications/nhs-reference-costs-2013-to-2014

For peer review only - http://bmjopen.bmj.com/site/about/guidelines.xhtml

16. Curtis LA: Unit costs for health and social care Personal Social Services Research Unit, University of Kent: Canterbury; 2014

17. EuroQol Group (2013) EuroQOl EQ5D User Guide v2.0 (downloaded June 2013)

18. Dolan P: Modeling valuations for EuroQol health states. *Medical Care* 1997 Nov;**35**(11):1095-108

19. Simons CL, Rivero-Arias O, Yu LM, Simon J. Multiple imputation to deal with missing EQ-5D-3L data: Should we impute individual domains or the actual index? *Qual Life Res* 2014; DOI 10.1007/s11136-014-0837

20. Schafer, J. L. Multiple imputation: A primer. Statistical Methods in Medical Research; 2009, **8**:3–15.

21. Glick HA, Doshi JA, Sonnad SS, Polsky D. Economic evaluation in clinical trials. (2 ed.) Oxford University Press 2014

22. Basu A, Manca A. Regression estimators for generic health-related quality of life and quality adjusted life years. *Med Decision Making* 2012; **32**: 56–69

23. Smith L, Gardner B, Fisher A, et al. (2014) Patterns and correlates of physical activity behaviour over 10 years in older adults: prospective analyses from the English Longitudinal Study of Ageing. *BMJ Open* 2015;5:e007423. doi:10.1136/bmjopen-2014- 007423

24. Public Health Guidance [PH44] Physical activity: brief advice for adults in primary care National Institute for Health and Care Excellence.

25. Office for National Statistics (ONS). Life Expectancy. ONS Digital 2015 (Accessed August 2015) Available from: http://visual.ons.gov.uk/how-long-will-my-pension-need-to-last/)

26. Laine J, Kuvaja-Kollner V, Pietila E, Koivuneva M, Valtonen H, Kankaanpdd E. Cost-Effectiveness of Population-Level Physical Activity Interventions: A Systematic Review *American Journal of Health Promotion* November/December 2014, **29(2):** 71–80

27. Garrett S, Raina Elley C, Rose SB. Are physical activity interventions in primary care and the community cost-effective? A systematic review of the evidence. *British Journal of General Practice* 2011; 61(584):e125-e133

28. de Smedt D, de Cocker K, Annemans L, de Bourdeaudhuij I, Cardon G. (2012) A cost-effectiveness study of the community-based intervention '10000 Steps Ghent' *Public Health Nutrition* **15(3):**442–51

29. Brennan A, Blake L, Hill-McManus D, Payne N, Buckley Woods H, Blank L. Walking and cycling: local measures to promote walking and cycling as forms of travel or recreation: Health economic and modelling report, National Institute for Health and Clinical Excellence Public Health Programme; 2012. Available from: https://www.nice.org.uk/guidance/ph41/evidence/economic-modelling-report-430262893

30. National Institute for Health and Care Excellence (NICE), Public Health Guidance [PH41] Physical activity: walking and cycling (2012). Available from: https://www.nice.org.uk/guidance/ph41/resources/physical-activity-walking-and-cycling-pdf-1996352901061

For peer review only - http://bmjopen.bmj.com/site/about/guidelines.xhtml

Table 1: Average costs and QALYs per participant, by trial arm, (£'sterling2013/14, all randomised participants who provided required accelerometry data*, missing data imputed)

Cost and quality of life (EQ5D5L)	Control	Postal	Nurse
	Mean (SD)		
**0-3 months**	**n=318**	**n=317**	**n=319**
Total cost	*£107 (254)*	*£122(107)*	*£249 (215)*
Set up	£0 (0)	£45(0)	£105(0)
Delivery of intervention	£0 (0)	£7 (0)	£50 (18)
Health service use	£107(254)	£71(107)	£95 (214)
EQ 5D scores at baseline	0.839 (0.14)	0.853 (0.12)	0.851 (0.12)
EQ 5D scores at 3 months	0.844 (0.14)	0.848 (0.14)	0.841 (0.14)
QALYs 0-3 months	0.194 (0.03)	0.196 (0.03)	0.195 (0.03)
**0-12 months**	**n=323**	**n=312**	**n=321**
Total cost	*£461 (916)*	*£375(611)*	*£603 (987)*
Set up	£0 (0)	£45 (0)	£105 (0)
Delivery of intervention	£0 (0)	£10 (0)	£52 (18)
Health service use	£461 (916)	£320 (611)	£447 (987)
EQ 5D scores at baseline	0.837 (0.14)	0.850 (0.12)	0.849 (0.13)
EQ 5D scores at 3 months	0.840 (0.14)	0.847 (0.13)	0.837 (0.14)
EQ 5D scores at 12 months	0.833 (0.15)	0.836 (0.13)	0.831 (0.14)
QALYs 0-12 months	0.837 (0.13)	0.843 (0.11)	0.836 (0.13)

*The number of people who provided accelerometry data differed across time points within arms * For incremental analyses, the comparisons are postal vs control and nurse vs control

For peer review only - http://bmjopen.bmj.com/site/about/guidelines.xhtml

Table 2: Regression estimates for costs, effects and cost-effectiveness at 3 and 12 months(£'sterling 2013/14) (base case, adjusted for baseline differences)

Cost, effects or cost-effectiveness		Control		Postal*		Nurse *		Nurse vs Postal	
		**Mean**	**(95% CI)**	**Mean**	**(95% CI)**	**Mean**	**(95% CI)**	**Mean**	**(95% CI)**
Costs and effects over 3 months	Total cost per participant (£)	108	(80 to 136)	123	(111 to135)	244	(221 to 266)	-	
	Incremental cost (£)	-		15	(-15 to 45)	135	(99 to 171)	120	(95 to 146)
	Total QALYs per participant	0.1957	(0.1936 to 0.1978)	0.1952	(0.1930 to 0.1974)	0.1948	(0.1926 to 0.1970)	-	
	Incremental* QALYs	-		-0.0005	(-0.0027 to 0.0016)	-0.0009	(-0.0031 to 0.0012)	-0.0004	(-0.0026 to 0.0018)
	Incremental daily steps			692	(363 to 1020)	1172	(844 to 1501)	481	(153 to 809)
	Incremental weekly mins of MVPA in bouts of ≥10 mins			43	(26 to 60)	61	(44 to 78)	18	(1 to 35)
Costs and effects over 12 months	Total cost per participant (£)	467	(365 to 569)	376	(307 to 445)	593	(473 to 714)	-	
	Incremental cost (£)	-		-91	(-215 to 33)	126	(-37 to 290)	217	(81 to 354)
	Total QALYs per participant	0.842	(0.832 to 0.853)	0.838	(0.827 to 0.849)	0.836	(0.824 to 0.847)	-	
	Incremental QALYs	-		-0.004	(-0.017 to 0.009)	-0.007	(-0.020 to 0.007)	-0.002	(-0.016 to 0.011)
	Incremental daily steps	-		642	(329 to 955)	677	(365 to 989)	36	(-227 to 349)
	Incremental weekly mins of MVPA in bouts of ≥10 mins	-		33	(17 to 49)	35	(19 to 51)	2	(-14 to 17)
IC	Cost per additional QALY (£)	-		*Postal dominated by control*		*Nurse dominated by control*		*Nurse dominated by Postal*	

For peer review only - http://bmjopen.bmj.com/site/about/guidelines.xhtml

Cost, effects or cost-effectiveness		Control Mean	Control (95% CI)	Postal* Mean	Postal* (95% CI)	Nurse* Mean	Nurse* (95% CI)	Nurse vs Postal Mean	Nurse vs Postal (95% CI)
	Cost per additional step count (£)	-		£0.02		£0.12		£0.25	
	Cost per additional minute of MVPA in a bout of ≥10 mins (£)	£0.35		£0.35		£2.21		£6.67	
ICER* at 12 months	Cost per additional QALY (£)	-		*Postal is less costly but has fewer QALYs. £21,162 saved per QALY lost*		*Nurse dominated by control*		*Nurse dominated by Postal*	
ICER* at 12 months	Cost per additional step count (£)	-		*Postal dominates control*		0.19		6.03	
ICER* at 12 months	Cost per additional minute of MVPA in a bout of ≥10 mins (£)	-		*Postal dominates control*		3.61		109.00	

For peer review only

For peer review only - http://bmjopen.bmj.com/site/about/guidelines.xhtml

Table 3: Costs, effects and cost-effectiveness over a lifetime from age 59 (100,000 cohort)

	Control	Postal*	Nurse *	Nurse vs Postal
	Mean<br>(95% CI)	Mean<br>(95% CI)	Mean<br>(95% CI)	Mean<br>(95% CI)
Lifetime total cost (£million) **	340<br>(307, 371)	329<br>(296, 361)	351<br>(318, 384)	-
Lifetime incremental cost (£million)	-	-11<br>(-12, -10)	11<br>(10, 12)	22<br>(21 to 23)
Lifetime total QALYs (million)	1.07<br>(0.89, 1.30)	1.07<br>(0.89, 1.30)	1.07<br>(0.89, 1.30)	-
Lifetime incremental QALYs	-	759<br>(400, 1247)	671<br>(346, 1071)	-108<br>(-223 to -10)
Lifetime ICER for QALYs (£)		Postal dominates control	16,368	Postal dominates nurse
Lifetime Incremental Net Monetary Benefit (£million, @ £20,000 per QALY)	-	26<br>(18, 36)	2<br>(-5, 11)	-24<br>(-27 to -21)

* For incremental analyses, the comparisons are postal vs control and nurse vs control.

**£46.7m, £37.6m and £59.3m of the total costs for control, postal and nurse groups respectively, were estimated using PACE-UP trial results

For peer review only - http://bmjopen.bmj.com/site/about/guidelines.xhtml

Figure Legends

Figure 1: Cost-effectiveness acceptability curve showing the probability of short-term (at 1 year) cost-effectiveness for postal and nurse groups (vs control) at different willingness to pay per QALY thresholds.

Figure 2: Cost-effectiveness acceptability curve showing the probability of life-time cost-effectiveness for postal and nurse groups (vs control) at different willingness to pay per QALY threshold levels

For peer review only

For peer review only - http://bmjopen.bmj.com/site/about/guidelines.xhtml

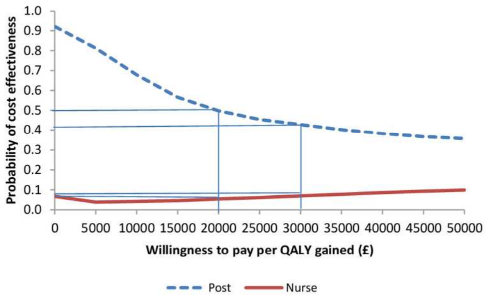

Figure 1: Cost-effectiveness acceptability curve showing the probability of short-term (at 1 year) cost-effectiveness for postal and nurse groups (vs control) at different willingness to pay per QALY thresholds.

140x198mm (300 x 300 DPI)

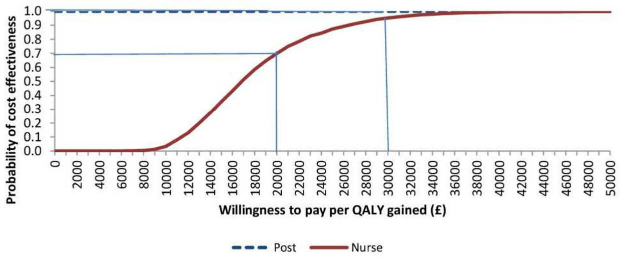

Figure 2: Cost-effectiveness acceptability curve showing the probability of life-time cost-effectiveness for postal and nurse groups (vs control) at different willingness to pay per QALY threshold levels

140x198mm (300 x 300 DPI)

Supplementary file for

"The short-term and long-term cost-effectiveness of a pedometer-based intervention in primary care: A within-trial analysis and beyond-trial modelling"

Nana Anokye PhD, Julia Fox-Rushby PhD, Sabina Sanghera PhD, Derek G. Cook PhD, Elizabeth Limb MSc, Cheryl Furness MSc, Sally Kerry PhD, Christina Victor PhD, Steve Iliffe FRCGP, Michael Ussher PhD, Peter H.Whincup PhD, Ulf Ekelund PhD, Steve DeWilde PhD, Tess Harris MD

For peer review only

Table S1: Resource use and cost components of 'Set-up Cost'*

**Activity (trial arm applicable to)**	**Resource**	**Total quantity**	**Cost per participant £ (nurse group)**	**Cost per participant £ (post group)**
**Design^**				
Designing of intervention (Both intervention arms)	Professor x1	0·5 days	4·43	4·43
	Readers x3	1 day		
	Senior lecturers x3	3·5 days		
	Consultants x2	1 days		
Designing of participants' handbooks and diaries (both intervention groups)	Professor x3	1·5 days	3·56	3·56
	Readers x2	1 day		
	Senior lecturers x3	2 days		
	Consultants x2	0·5 days		
Designing of nurse trainers handbooks (Nurse group)	Senior lecturers x1	1 day	2·74	0
	Consultants x1	0·5 days		
	Handbooks	9 handbooks	0·19	0
**Setting up GP practices**				
Planning for recruitment of practices (All trial arms)	Professor x1	1 hour	0·99	0·99
	Senior lecturer x1	5 hours		
	Consultants x2	5 hours		
Visits to recruit 6 practices (All trial arms)	Senior lecturers x2	13 hours	1·47	1·47
	Trial Manager x1	7 hours		
	Consultant x1	5 hours		
	Round trips to practices (by all)	25 hours	0·10	0·10
Searching practice computers to identify participants (All trial arms)	Senior lecturer x1	6 hours	0·71	0·71
	Trial Manager x1	6 hours		
	Practice Manager x6	6 hours		
Identify households from anonymised address list (All trial arms)	Senior lecturer x1	32 hours	2·28	2·28
	Trial Manager x1	32 hours		
Practice staff reviews lists for exclusion (All trial arms)	GP x5 (for sorting out 2 practices)	20 hours	4·50	4·0

Activity (trial arm applicable to)	Resource	Total quantity	Cost per participant £ (nurse group)	Cost per participant £ (post group)
	Nurse x10 (for sorting out other 5 practices)	50 hours	1·96	1·96
Printing letters at practice (All trial arms)	Trial Manager x1	64 hours	1·57	1·57
	Practice administrative staff x2	4 hours		
	Number of printed letters	24000	0·94	0·94
Packing envelopes with leaflets and letters (All trial arms)	Trial Manager x1	240 hours	7·04	7·04
	Research Assistants x2	56 hours		
	Practice admin. Staff x11	27·5 hours		
	Cost of Envelopes	£497·30	0·49	0·49
	Cost of Postal stamps	£5,530·50	5·41	5·41
	Cost of Information leaflets	£5,973·00	5·84	5·84
Preparing rooms at practices for trial (All trial arms)	Round trip to practices by RA	14 trips	0·04	0·04
	Research Assistants x2	-*	0·11	0·11
**Training**				
Training of Trial manager (All trial arms)	Trial Manager x1	4 days	1·51	1·51
	Senior lecturer x1	2 days		
Preparation of nurse training course (Nurse support group)	Trial Manager x1	1 day	9·63	0
	Senior lecturer x1	2 days		
	Reader x1	0·5 days		
	Consultants x2	2 days		
Mini-training day of nurses (Nurse group)	Nurses x11	33 hours	7·46	0
	Trial Manager x1	17·33 hours		
	Senior lecturer x1	17·33 hours		
	Round trips to training centre (by tutors)	16 hours	0·19	0
	Pedometers given to nurses	12 hours	0·04	0
Full training day of nurses (Nurse group)	Nurses x10	107·5 hours	22·99	0
	Reader x1	1 hour		
	Senior lecturer x1	10 hours		
	Consultants x2	22·5 hours		
	Round trips for training by nurses x10	10 trips	0·12	0
	Round trips for training by consultants x2	2 trips	0·13	0
	Refreshments	1 set	0·26	0
Training for an absentee nurse (Nurse group)	Nurse x 1	10 hours	2·47	0
	Trial Manager x1	11·33 hours		
	Research assistant x1	11·33 hours		
	Round trips to training centre	2 trips	0·02	0
Discussion of nurses recorded sessions(Nurse group)	Senior lecturer x1	0·5 days	3·78	0
	Consultants x2	1 day		
	Nurses x9	4·5	0·99	0
	Senior lecturer x1	0·5		

For peer review only

**Activity (trial arm applicable to)**	**Resource**	**Total quantity**	**Cost per participant £ (nurse group)**	**Cost per participant £ (post group)**
	Consultants x2	1		
	Duration of phone calls	270 mins	0·09	0
Follow-up half day training(Nurse group)	Nurses x 9	4.5 days	7·70	0
	Trial Manager x1	0·5 days		
	Senior lecturer x1	0·5 days		
	Consultants x2	1 day		
	Nurse time travelling x 9	6·75 hours	0·78	0
	Round trips to training centre (nurses)	9 trips	0·10	0
	Refreshment	1 set	0·15	0
Training of Research assistants (All trial arms)	Research assistant x3	6·6 days	1·91	1·91
	Senior lecturer x1	0·5 days		
	Reader x1	0·5 days		
	Trial Manager x1	4 days		
Total cost per participant			104·64	44·83

^ Design was included as materials couldn't be used wholesale from a previous study and we judged that this may occur in the future following further learning from this trial*Value removed at present to maintain confidentiality
*Data source: Interviews with trial PI and trial manager, review of trial records, diaries, and routine administrative records

For peer review only

Table S2: Components of delivery cost of intervention (Post group)

Components	Resource (from administrative records)	Quantity of resource	Unit cost (data source)	3 months analysis		12 months analysis	
				Total cost	Cost per participant	Total cost	Cost per participant
Envelopes for posting pedometers (including replacement)	Number of envelopes	426	£0·03 (invoice)	£12·78	£0·04	£12·78	£0·04
Stamps for posting pedometer	Number of stamps	426	£2·50 (invoice)	£1,065	£3·14	£1,065	£3·14
Pedometers (including replacements) given to participants	Number of pedometers	426	£1 / £4*(invoice)	£426	£1·26	£1,704	£5·03
Replacement batteries for pedometer	Number of replacement batteries	11	£0·67 (invoice)	£7·37	£0·02	£7·37	£0·02
Patient handbooks	Number of handbooks	339	£0·80 (administrative records)	£271	£0·80	£271	£0·80
Step count diary	Number of diaries	339	£1·30 (administrative records)	£440·70	£1·30	£440·70	£1v30
Total cost per participant					£6·56		£10·33

*£1 was pro rata unit cost for 3 months and £4 is for 12 months. As pedometers were required only for the period of analysis but could be used beyond, their costs were spread over their expected lifetime, following Sharples et al (2014)[1]. As pedometers had an expected lifetime of 2 years, the average cost of pedometer was multiplied by 13[1]/104[2](weeks), in the case of 3 month analysis and 52/104 for the 12 month analysis.

[1] Intervention period in weeks

[2] Life expectancy of pedometer (in weeks)- based experience from PACE lift trial

For peer review only - http://bmjopen.bmj.com/site/about/guidelines.xhtml

For peer review only

Table S3: Components of delivery cost of intervention (Nurse group)

Components	Resource (data source)	Quantity of resource	Unit cost (data source)	3 months analysis		12 months analysis	
				**Total cost**	**Cost per participant**	**Total cost**	**Cost per participant**
Pedometers given to participants	Number of pedometers (administrative records)	346	£1 / £4* (Invoice)	£346	£1	£1384	£4
Patient handbooks	Number of handbooks (administrative records)	346	£0·80 (administrative records)	£277	£1	£277	£1
Step count diary	Number of diaries (administrative records)	346	£1·30 (administrative records)	£449·80	£1·30	£449·80	£1·30
RAs time to arrange consultation	Time spent by RAs (diary)	50·46 hours	£16·51 (administrative records)	£833·07	£2·41	£833·07	£2·41
Phone calls by RA to arrange consultation	Duration of phone calls (administrative records)	3,027·5 mins	£0·11 (BT tariff)	£333·03	£0·96	£333·03	£0·96
Cost of nurse visit per participant (project database for nurse group)					£43		£42
Total cost per participant					£49·67		£51·67

*£1 was pro rata unit cost for 3 months and £4 is for 12 months.

For peer review only - http://bmjopen.bmj.com/site/about/guidelines.xhtml

Table S4: Costs to participants of participating in interventions and physical activity

Participant costs	Control (n=323)	Post (n=312)	Nurse (n=321)
	£ Mean (SD)		
Intervention related			
Time working out how to use pedometer	0(0)	2 (6)	1 (3)
Time planning how to increase walking/step count	0(0)	5 (15)	3 4)
Time filling in PACE-UP diary	0(0)	51 (80)	58 (122)
Parking fees to visit nurse	0(0)	0(0)	0·11 (0.73)
Time spent in consultation with nurse	0(0)	0(0)	10 (5)
Time travelling (irrespective of mode of transport) to visit nurse	0(0)	0(0)	11 (10)
Transportation cost (for those who took public transport) of attending the nurse visit	0(0)	0(0)	0·13 (1.33)
Time waiting time prior to consultation with nurse	0(0)	0(0)	3 (4)
Child care during nurse visits	0(0)	0(0)	0·3 (3.21)
Personal costs of participation in physical activity	411 (817)	492 (1,293)	333 (684)
Personal costs from falls/ fractures/ sprains/ injuries	17 (103)	22 (184)	6 (40)

For peer review only

For peer review only - http://bmjopen.bmj.com/site/about/guidelines.xhtml

Table S5: Health service use by trial arm with unit costs

Health service use	Trial arm (Qty)			Unit cost (£) Weighted average (Q1 – Q3)	Source of unit cost
	Control n=323	Post n=312	Nurse n=321		
**Outpatient referrals (total)[2]**	**164**	**158**	**186**		
Opthalmology	10	18	15	86 (70-99)	DH (2015) National Reference Costs
Urology	4	3	6	99 (76-116)	
General medicine	4	0	2	157 (120-187)	
ENT	9	6	12	92 (70-109)	
Podiatry	9	7	7	44 (27-45)	
Trauma & orthopaedics	14	13	10	113 (88-133)	
Physiotherapy	26	33	37	46 (35-50)	
Nephrology	0	1	0	145 (94-178)	
Oral surgery	0	2	0	115 (85-142)	
Gynaecology	6	7	14	134 (104-164)	
Audiology	4	6	7	104 (55-174)	
Colorectal surgery	1	5	1	117 (83-135)	
Neurology	8	8	5	174 (136-204)	
Cardiology	12	5	4	131 (92-154)	
Gastroenterology	6	2	6	130 (99-153)	
Rheumatology	4	6	7	135 (99-150)	
Dermatology	1	8	7	98 (74-109)	
General surgery	4	1	3	125 (98-165)	
Endocrinology	2	1	2	144 (100-167)	
Neurosurgery	2	0	0	181 (138-228)	
Oncology	8	5	11	133 (97-165)	
Psychotherapy	1	0	0	100 (47-217)	
Respiratory medicine	4	6	3	150 (107-181)	
Clinical neurophysiology	2	0	1	165 (107-197)	
Programmed pulmonary rehab	0	0	1	20 (12-31)	
Pain management	2	0	4	135 (82-164)	
Allergy service	0	1	0	149 (126-175)	
Dietetics	2	2	3	62 (38-76)	
Vascular surgery	2	1	4	149 (100-176)	
Mental illness	1	1	1	234 (181-256)	
Clinical Genetics	1	0	1	429 (248-601)	
Clinical Haematology	2	1	0	160 (93-189)	
Spinal surgery services	0	1	0	142 (112-164)	
Maxillo-facial surgery	0	0	1	111 (70-133)	
Plastic surgery	1	1	1	93 (68-109)	
Clinical immunology	0	1	0	215 (140-243)	
Interventional radiology	1	0	0	192 (88-260)	
Breast surgery	9	4	5	139 (103-166)	
Tropical medicine	0	1	0	202 (203-203)	
Clinical psychology	1	0	3	177 (116-245)	
Old age psychiatry	0	1	2	108 (108-108)	
Referral to Accident & Emergency	1	0	0	135 (54-166)	
**Community based referrals (total)[3]**	**27**	**19**	**21**		
District nurse	1	3	2	39 (31-43)	PSSRU
Community Podiatrist	4	3	8	42 (35-58)	PSSRU
Community Dietitian	0	2	0	80 (53-96)	DH (2015) National Reference Costs
Smoking cessation (Nurse)	5	3	4	14	15.5 mins nurse time (Curtis 2014)
Healthy lifestyle (Nurse)	0	2	0	14	15.5 mins nurse time (Curtis 2014)
Community Gynaecologist	5	1	0	134 (104-164)	DH (2014) National Reference Costs
Community Physiotherapist	7	4	1	52 (44-58)	(Curtis 2014)
Community Diabetic	1	0	0	69 (38-93)	DH (2015) National Reference Costs
DESMOND diabetes programme	4	0	6	230	Gillett et al (2010) (inflated to 2014)
Expert Patient Programme	0	1	0	302	Richardson et al (2008) (inflated to 2014)
**Primary Care – excludes practice**	**2074**	**1748**	**2094**		

For peer review only

**Health service use**	**Trial arm (Qty)**			**Unit cost (£) Weighted average (Q1 – Q3)**	**Source of unit cost**
	**Control n=323**	**Post n=312**	**Nurse n=321**		
**visits related to the delivery and participation in intervention (total)**[1]					
GP (11.7mins)	1743	1436	1729	42	(Curtis 2014)
GP nurse (15.5mins)	331	312	365	14	(Curtis 2014)
**A&E visit**[4]	49	36	46	124	DH (2015) National Reference Costs
**Non- Elective hospital admissions (total)**[5,6]	**12**	**4**	**20**		
Biliary acute pancreatitis	0	0	3	2037 (1247-2492)	DH (2015) National Reference Costs
Cardiac catheterisation for coronary artery disease	1	0	1	2643 (1980-3028)	
Chest pain	0	1	0	490 (370-563)	
Abdominal pain	0	0	1	718 (922 -1298)	
Acute ST segment elevation myocardial infarction	2	0	0	1497 (1102-1740)	
Transient ischaemic attack	0	0	1	878 (643-994)	
Guillain-Barre syndrome	0	0	1	1571 (1069-1792)	
Pneumonia	1	0	0	1894 (1406-2238)	
Epilepsy	1	0	0	1125 (788-1266)	
Stroke and cerebrovascular accident	1	0	0	2817 (2018-3396)	
UTI	0	0	1	1530 (1187-1755)	
Detached Retina	0	0	1	908 (303-1935)	
Anxiety states	0	0	1	1393 (984-1628)	
Infective endocarditis in diseases EC, NOS	1	0	0	4480 (2351-5906)	
Acute appendicitis	0	0	1	3017 (2459-3365)	
IUD removed	0	0	1	1780 (1142-2135)	
Ankle fracture	1	0	0	3762 (3109-4271)	
no procedure (NES)	4	3	8	611 (408-726)	
**Elective hospital admissions (total)**[5,7]	**10**	**2**	**3**		
Cardiac catheterisation	2	0	0	2086 (1185-2709)	DH (2015) National Reference Costs
Percut tranlum balloon angioplasty mult coronary	1	0	0	1813 (880-2233)	
Inguinal hernia	0	1	0	2121 (1682-2392)	
Coronary artery bypass graft operations	0	1	0	9310 (7369-9929)	
Laparoscopic cholecystectomy	3	0	0	2567 (2082-2924)	
Endarterectomy of femoral artery NEC	0	0	2	6028 (4593-7209)	
Malignant neoplasm of female breast for chemotherapy	1	0	0	1780 (856-2139)	
Endarterectomy of carotid artery NEC	1	0	0	3911 (2986-4497)	
Neurophysiological operation NOS	2	0	0	1497 (1111-2118)	
Ovarian Cancer	0	0	1	1469 (741-1966)	
Total resource use (All HSU)	2336	1967	2370		

Unit costs are rounded to the nearest whole number and presented in the 2013/14 price year. The health service use presented in this table refers to the base case sample. All the data are based on participant-specific GP records for the trial period with different assumptions and approaches for costing by type of service use:

[1]*Primary care*: GP visits 11.7 minutes; Nurse visits 15·5 minutes;

[2]*Outpatient referrals*: where appropriate, linked to outpatient service descriptions in the reference costs (and reviewed by principal investigator) and a weighted (by throughout) average for consultant/non-consultant led attendances taken; referrals to private sector excluded (n=1);

[3]community referral services costed as referenced; if service use was unclear, an NHS hospital out-patient department was assigned by the principal investigator;

[4]*A&E visit*: as reason for A&E visits was not recorded, an average A&E visit cost for 2013-14 was assigned.

For peer review only

For peer review only - http://bmjopen.bmj.com/site/about/guidelines.xhtml

[5]*Hospital admissions*: The principal investigator (blind to study group) reviewed all hospital admissions, and provided either a 'best guess diagnosis/procedure' or listed 'unknown' (n=2). As details on the type of procedure or severity of the symptoms were not available, a weighted (by activity) average of all of the possible scores/procedures was used to derive average cost for elective.

[6]The unit cost for the emergency admissions are a weighted average of the non-elective short stay and non-elective long stay admissions, as the length of stay was unclear.

[7]Hospital admissions without a procedure were treated as non-elective short stay admissions (one day or less). Where hospital admission code was unclear the diagnosis was reviewed by the PI for advice on the nearest appropriate code.

For peer review only

For peer review only - http://bmjopen.bmj.com/site/about/guidelines.xhtml

Figure S1: Illustration of pathways within the long-term cost-effectiveness model (Anokye et al 2014a)

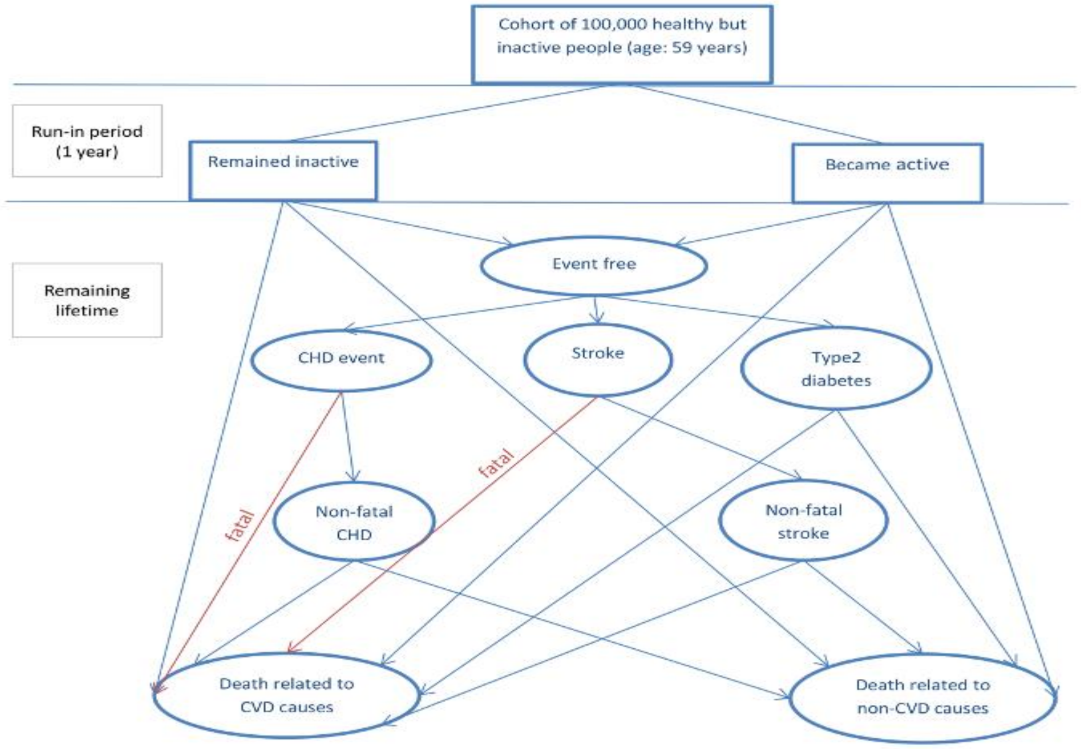

For peer review only - http://bmjopen.bmj.com/site/about/guidelines.xhtml

Table S6: Parameter values for long-term cost-effectiveness model

Parameter		Value	Source of data
Relative risks of:			
Becoming active (at year 1)*:	Postal vs control	1·8 (95% CI: 1·4, 2.3)	PACE-UP trial data
	Nurse vs control	1·7 (95% CI: 1·3, 2.2)	
	Nurse vs postal	0·9 (95% CI: 0·7, 1.3)	
Disease (active vs inactive)	CHD	0·90	Hu et al (2007)
	Stroke	0·86	Hu et al (2005)
	Diabetes	0·67	Hu et al (2003)
Non-CVD mortality after:	Non-fatal CHD	1·71	Bronum-Hansen et al (2001)
	Non-fatal Stroke	1·71	
	Diabetes	1·49	Preis et al (2009)
CVD mortality after:	Non-fatal CHD	3·89	Bronum-Hansen et al (2001)
	Non-fatal Stroke	3·89	
	Diabetes	2·61	Preis et al (2009)
CHD fatalities	59-64	11·55%	Ward et al (2005)
	65-74	21·07%	
	75+	14·76%	
Stroke fatalities	55-64	23·28%	Ward et al (2005)
	65-74	23·47%	
	75+	23·42%	
CHD incidence	59-64	0·63%	Ward et al (2005); NCGC (2011)
	65-74	0·97%	
	75+	0·97%	
Stroke incidence	59-64	0·29%	
	65-74	0·69%	
	75+	1·43%	
Diabetes incidence	59	0·06%	Gonzalez et al (2009)
	60-69	0·10%	
	70-79	0·11%	
	80+	0·11%	
Age-specific quality of life	59-64	0·82	Health Survey for England (2011)
	65-74	0·78	
	75+	0·72	
Health state utility weight	Healthy	1·00	Ward et al (2005); NCGC (2011)
	CHD 1st event	0·80	
	post CHD 1st event	0·92	
	Stroke 1st event	0·63	
	post stroke 1st event	0·65	
	Diabetes	0·90	
	Short term psychological benefit of achieving 150 mins of MVPA per week	0·01	PACE UP trial data
Annual costs	Control	£467 (95% CI 365 to569)	PACE UP trial data
	Postal	£376 (95% CI 307 to445)	

For peer review only - http://bmjopen.bmj.com/site/about/guidelines.xhtml

Parameter		Value	Source of data
	Nurse	£593 (95% CI 473 to714)	
	CHD 1st event	£4,248	NCGC (2011)
	post CHD 1st event	£485	
	Stroke 1st event	£10,968	
	post stroke 1st event	£2,409	
	Diabetes	£979	

*Relative risks (RR) for achieving at least 150 minutes of MVPA in ≥10 minute bouts at 12 months were estimated from odds ratios (OR) using the formula OR / {(1-P_{ref}) + (P_{ref} *OR)} where P_{ref} is the proportion of all subjects achieving 150 minutes of MVPA in ≥10 minute bouts at baseline i.e. 218/1023 = 0.21. The odds ratios had been derived from a logistic regression model in which the dependent variable, achieving 150 minutes of MVPA in bouts of ≥10 minutes at 12 months, was regressed on baseline minutes of MVPA in bouts of ≥10 minutes, month of baseline accelerometry, day order of wear, day of week, age, gender, general practice and treatment group, with household as a cluster.

For peer review only

Figure S2: Cost-effectiveness plane for postal vs control at 12 months

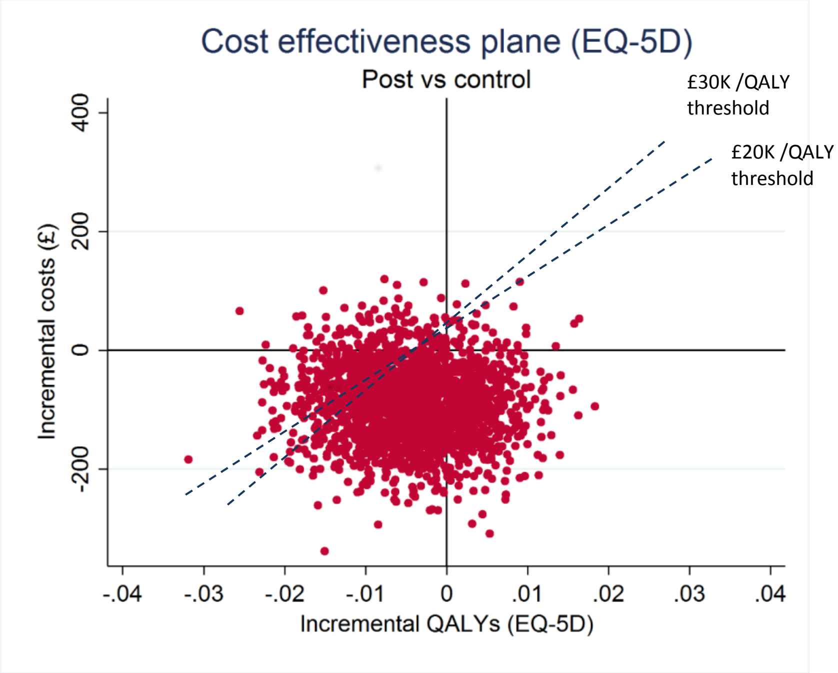

Figure S3: Cost-effectiveness plane for nurse vs control at 12 months

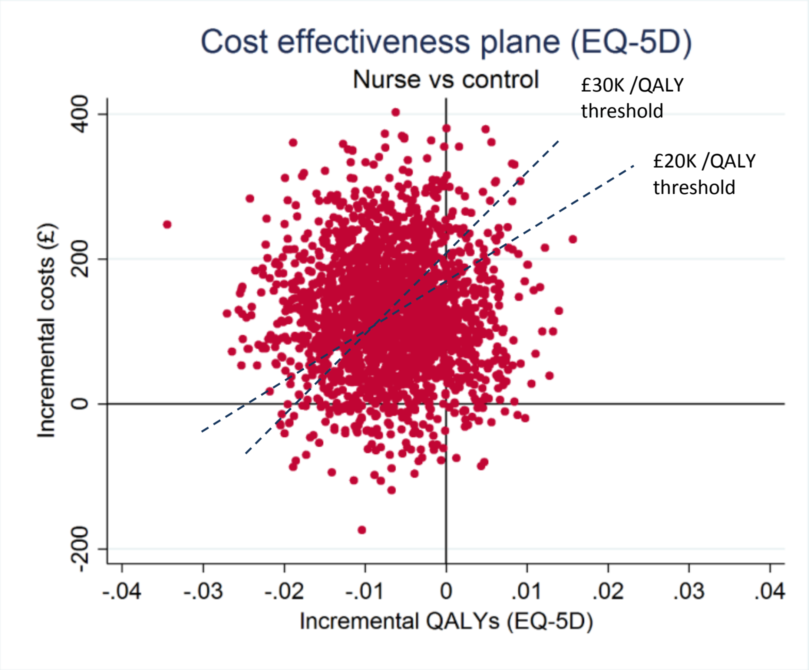

For peer review only - http://bmjopen.bmj.com/site/about/guidelines.xhtml

Figure S4: Cost-effectiveness plane for nurse vs post at 12 months

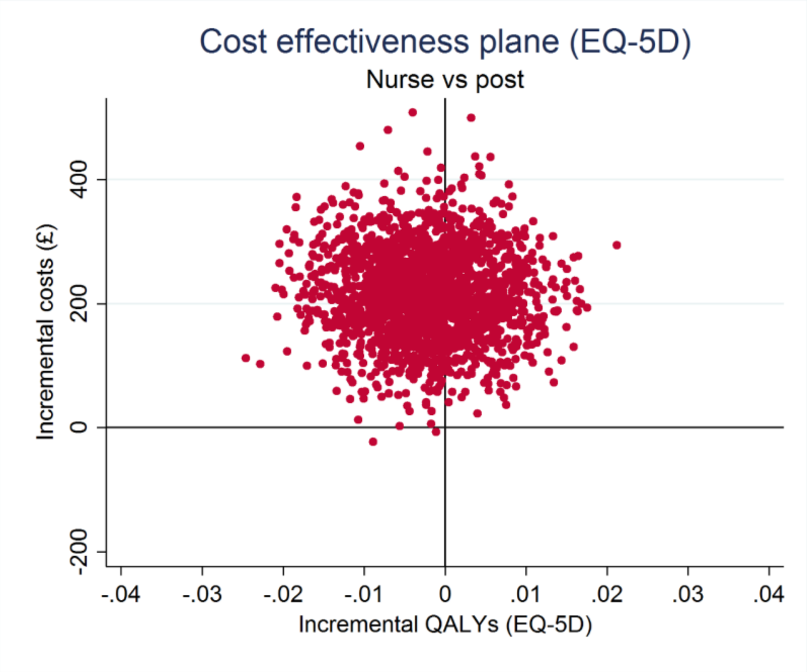

For peer review only

Table S7: Within trial sensitivity analyses (at 12 months)

Parameter	Post vs Control			Nurse vs Control			Nurse vs Post		
	**Incre-mental cost (£)**	**Incre-mental QALY**	**ICER**	**Incre-mental cost(£)**	**Incre-mental QALY**	**ICER**	**Incre-mental cost (£)**	**Incre-mental QALY**	**ICER**
	**Mean (95% CI)**			**Mean (95% CI)**			**Mean (95% CI)**		
Base case	-91 (-215, 33)	-0.0043 (-0.0172, 0.0087)	Less costly but less effective than control	126 (-37, 290)	-0.0066 (-0.0201, 0.0068)	Intervention dominated by control	£217 (8, 354)	-0.0024 (-0.0156, 0.0109)	*Nurse dominated by Post*
Whole sample (all randomised)	-40 (-169, -89)	-0.0070 (-0.0195, 0.0054)	Less costly but less effective than control	150 (-6, 306)	-0.0093 (-0.0222, 0.0036)	Intervention dominated by control	190 (48, 332)	-0.0023 (-0.0148, 0.0102)	*Nurse dominated by Post*
Health service use including only GP data on referrals and admissions	-55 (-166, -56)	-0.0043 (-0.0172, 0.0087)	Less costly but less effective than control	129 (-17, 275)	-0.0066 (-0.020, 0.0068)	Intervention dominated by control	184 (61, 307)	-0.0024 (-0.0156, 0.0109)	*Nurse dominated by Post*
Health service use including only self-reported serious adverse effects	21 (-65, 107)	-0.0043 (-0.0172, 0.0087)	Intervention dominated by control	144 (65, 224)	-0.0066 (-0.020, 0.0068)	Intervention dominated by control	123 (47, 200)	-0.0024 (-0.0156, 0.0109)	*Nurse dominated by Post*
Health service use including only GP data on adverse effects	-11 (-107, 85)	-0.0043 (-0.0172, 0.0087)	Less costly but less effective than control	64 (-15, 142)	-0.0066 (-0.020, 0.0068)	Intervention dominated by control	74 (13, 135)	-0.0024 (-0.0156, 0.0109)	*Nurse dominated by Post*
Excluding all health service use cost	55.2 (55, 55.4)	-0.0043 (-0.0172, 0.0087)	Intervention dominated by control	156.2 (-154, 158)	-0.0066 (-0.0201, 0.0068)	Intervention dominated by control	101 (99, 103)	-0.0024 (-0.0156, 0.0109)	*Nurse dominated by Post*
Changing cost perspective (both participants (all participant costs) and NHS costs)	36 (-177, 250)	-0.0043 (-0.0172, 0.0087)	Intervention dominated by control	107 (-97, 311)	-0.0066 (-0.020, 0.0068)	Intervention dominated by control	71 (-150, 291)	-0.0024 (-0.0156, 0.0109)	*Nurse dominated by Post*
Changing cost perspective (both participants (part)[3] and NHS costs)	-22 (-235, 191)	-0.0043 (-0.0172, 0.0087)	Less costly but less effective than control	47 (-157, 250)	-0.0066 (-0.020, 0.0068)	Intervention dominated by control	69 (-152, 289)	-0.0024 (-0.0156, 0.0109)	*Nurse dominated by Post*
Combination of excluding all health service use cost and including all participants costs (minus health service use cost borne by participants)	179 (-1, 361)	-0.0043 (-0.0172, 0.0087)	Intervention dominated by control	153 (24, 281)	-0.0066 (-0.020, 0.0068)	Intervention dominated by control	-27 (-203, 149)	-0.0024 (-0.0156, 0.0109)	Less costly but less effective than control
Pedometer lasts for 1 year (equivalent to pedometers not being re-usable and full cost of pedometer borne in year 1)	-86 (-210, 38)	-0.0043 (-0.0172, 0.0087)	Less costly but less effective than control	130 (-33, 294)	-0.0066 (-0.0201, 0.0068)	Intervention dominated by control	216 (80, 353)	-0.0024 (-0.0156, 0.0109)	*Nurse dominated by Post*
Pedometer lasts for 4 years (double length of life considered in base case)	-93 (-218, 31)	-0.0043 (-0.0172, 0.0087)	Less costly but less effective than control	124 (-39, 287)	-0.0066 (-0.0201, 0.0068)	Intervention dominated by control	218 (81, 354)	-0.0024 (-0.0156, 0.0109)	*Nurse dominated by Post*

[3] This excludes time costs of working out how to use pedometer, diary, and planning to increase work

For peer review only - http://bmjopen.bmj.com/site/about/guidelines.xhtml

References

Anokye NK., Lord J., Fox-Rushby J. Is brief advice in primary care a cost-effective way to promote physical activity?, *British Journal of Sports Medicine* 2014; **48 (3)** : 202–206

Brønnum-Hansen H, Davidsen M, Thorvaldsen P. Long-term survival and causes of death after stroke. *Stroke* 2001; **32**: 2131–6.

Curtis LA: Unit costs for health and social care Personal Social Services Research Unit, University of Kent: Canterbury; 2014.

De Cocker K, De Bourdeaudhuij I, Brown W, Cardon G. Moderators and mediators of pedometer use and step count increase in the "10,000 Steps Ghent" intervention. *International Journal of Behavioral Nutrition and Physical Activity* 2009; **6**:3 http://dx.doi.org/10.1186/1479-5868-6-3

Department of Health (2015). NHS Reference Costs 2013 to 2014. Available from:https://www.gov.uk/government/publications/nhs-reference-costs-2013-to-2014

Gillett M, Dallosso HM, Dixon S, et al. Delivering the diabetes education and self- management for ongoing and newly diagnosed (DESMOND) programme for people with newly diagnosed type 2 diabetes: cost effectiveness analysis. *BMJ 2010*: 341:c4093

Gonzalez ELM, Johansson S, Wallander MA. Trends in the prevalence and incidence of diabetes in the UK, 1996–2005. *J Epidemiol Community Health* 2009; **63**:332–6.

Hu G, Jousilahti P, Borodulin K, et al. Occupational, commuting and leisure-time physical activity in relation to coronary heart disease among middle-aged Finnish men and women. *Atherosclerosis* 2007; **194**:490–7

Hu G, Sarti C, Jousilahti P, et al. Leisure time, occupational, and commuting physical activity and the risk of stroke. *Stroke* 2005; **36**:1994–9.

Hu G, Qiao Q, Silventoinen K, et al. Occupational, commuting, and leisure-time physical activity in relation to risk for type 2 diabetes in middle-aged Finnish men and women. *Diabetologia* 2003; **46**:322–29

Health Survey for England, 2011 National Centre for Social Research, University College London-Department of Epidemiology and Public Health. [computer file]. *3rd Edition.* Colchester, Essex: UK Data Archive [distributor], July 2011. SN: 6397

National Clinical Guideline Centre (NCGC). Hypertension: the clinical management of primary hypertension in adults. Clinical guideline: methods, evidence and recommendations. London: National Institute for Health and Clinical Excellence. 2011. Ref Type: Report.

Preis SR, Hwang SJ, Coady S, et al. Trends in all-cause and cardiovascular disease mortality among women and men with and without diabetes mellitus in the Framingham Heart Study, 1950 to 2005. *Circulation* 2009; **119**:1728–35.

Richardson G, Kennedy A, Reeves D, et al. A Cost effectiveness of the Expert Participants Programme (EPP) for participants with chronic conditions. *Journal of Epidemiology and Community Health* 2008; **62**: 361–367

For peer review only - http://bmjopen.bmj.com/site/about/guidelines.xhtml

Sharples L, Glover M, Bennett M, Jordan J, Clutterbuck-James A, Davies M, Smith I, Cameron M, Chadwick R, Oscroft N, Morrell M, Fox-Rushby J, Pittman M, Quinnell T (2014) TOMADO: A Crossover Randomised Controlled Trial of Oral Mandibular Advancement Devices for Obstructive Sleep Apnoea-Hypopnoea *Health Technology Assessment 18, 67,* *http://www.journalslibrary.nihr.ac.uk/hta/volume-18/issue-67#*

Ward S, Jones ML, Pandor A, et al. Statins for the prevention of coronary events. London. National Institute for Clinical Excellence 2005; Ref Type: Report World Health Organisation (2010) Global recommendations on physical activity for health http://www.who.int/dietphysicalactivity/publications/9789241599979/en/

For peer review only - http://bmjopen.bmj.com/site/about/guidelines.xhtml

CHEERS Checklist
Items to include when reporting economic evaluations of health interventions

The **ISPOR CHEERS Task Force Report**, *Consolidated Health Economic Evaluation Reporting Standards (CHEERS)—Explanation and Elaboration: A Report of the ISPOR Health Economic Evaluations Publication Guidelines Good Reporting Practices Task Force,* provides examples and further discussion of the 24-item CHEERS Checklist and the CHEERS Statement. It may be accessed via the *Value in Health* or via the ISPOR Health Economic Evaluation Publication Guidelines – CHEERS: Good Reporting Practices webpage: http://www.ispor.org/TaskForces/EconomicPubGuidelines.asp

Section/item	Item No	Recommendation	Reported on page No/ line No
**Title and abstract**			
Title	1	Identify the study as an economic evaluation or use more specific terms such as "cost-effectiveness analysis", and describe the interventions compared.	P1 L1-2
Abstract	2	Provide a structured summary of objectives, perspective, setting, methods (including study design and inputs), results (including base case and uncertainty analyses), and conclusions.	P2 L1-39
**Introduction**			
Background and objectives	3	Provide an explicit statement of the broader context for the study.<br>Present the study question and its relevance for health policy or practice decisions.	P4 all & P5L4-5
**Methods**			
Target population and subgroups	4	Describe characteristics of the base case population and subgroups analysed, including why they were chosen.	P4L1-21 & P2L11-12
Setting and location	5	State relevant aspects of the system(s) in which the decision(s) need(s) to be made.	P2L23 - P3L5
Study perspective	6	Describe the perspective of the study and relate this to the costs being evaluated.	P6L14, P6L29-30
Comparators	7	Describe the interventions or strategies being compared and state why they were chosen.	P5 L16-21
Time horizon	8	State the time horizon(s) over which costs and consequences are being evaluated and say why appropriate.	P6L4-12&21, P6L16
Discount rate	9	Report the choice of discount rate(s) used for costs and outcomes and say why appropriate.	P6L7, P7L17
Choice of health outcomes	10	Describe what outcomes were used as the measure(s) of benefit in the evaluation and their relevance for the type of analysis performed.	P6L10-14, P7L23-29
Measurement of effectiveness	11a	*Single study-based estimates:* Describe fully the design features of the single effectiveness study and why the single study was a sufficient source of clinical effectiveness data.	P4L14-16

	11b	*Synthesis-based estimates:* Describe fully the methods used for identification of included studies and synthesis of clinical effectiveness data.	P7L15-P8L14, ref 13
Measurement and valuation of preference based outcomes	12	If applicable, describe the population and methods used to elicit preferences for outcomes.	Na
Estimating resources and costs	13a	*Single study-based economic evaluation:* Describe approaches used to estimate resource use associated with the alternative interventions. Describe primary or secondary research methods for valuing each resource item in terms of its unit cost. Describe any adjustments made to approximate to opportunity costs.	P5L24-P6L8 TablsS1-5
	13b	*Model-based economic evaluation:* Describe approaches and data sources used to estimate resource use associated with model health states. Describe primary or secondary research methods for valuing each resource item in terms of its unit cost. Describe any adjustments made to approximate to opportunity costs.	P7L25, P8L7-8 Ref 13
Currency, price date, and conversion	14	Report the dates of the estimated resource quantities and unit costs. Describe methods for adjusting estimated unit costs to the year of reported costs if necessary. Describe methods for converting costs into a common currency base and the exchange rate.	P6L6-7, P7L16
Choice of model	15	Describe and give reasons for the specific type of decision-analytical model used. Providing a figure to show model structure is strongly recommended.	P7L15-6,FigS1, P8Sup1
Assumptions	16	Describe all structural or other assumptions underpinning the decision-analytical model.	SuplTS6(p10-11)
Analytical methods	17	Describe all analytical methods supporting the evaluation. This could include methods for dealing with skewed, missing, or censored data; extrapolation methods; methods for pooling data; approaches to validate or make adjustments (such as half cycle corrections) to a model; and methods for handling population heterogeneity and uncertainty.	P6L16-P7L12, P8L18-25
**Results**			
Study parameters	18	Report the values, ranges, references, and, if used, probability distributions for all parameters. Report reasons or sources for distributions used to represent uncertainty where appropriate. Providing a table to show the input values is strongly recommended.	P8L2-15, TblS1-S5
Incremental costs and outcomes	19	For each intervention, report mean values for the main categories of estimated costs and outcomes of interest, as well as mean differences between the comparator groups. If applicable, report incremental cost-effectiveness ratios.	Tabl1-3, P8L29-P10L2, P10L20-27
Characterising uncertainty	20a	*Single study-based economic evaluation:* Describe the effects of sampling uncertainty for the estimated incremental cost and incremental effectiveness parameters, together with the impact	P10L4-17, Fig1&2, TblS7

For peer review only

		of methodological assumptions (such as discount rate, study perspective).	Fig S2-4, P11L1-16
	20b	*Model-based economic evaluation:* Describe the effects on the results of uncertainty for all input parameters, and uncertainty related to the structure of the model and assumptions.	Na
Characterising heterogeneity	21	If applicable, report differences in costs, outcomes, or cost-effectiveness that can be explained by variations between subgroups of patients with different baseline characteristics or other observed variability in effects that are not reducible by more information.	P11L19-P12L15
**Discussion** Study findings, limitations, generalisability, and current knowledge	22	Summarise key study findings and describe how they support the conclusions reached. Discuss limitations and the generalisability of the findings and how the findings fit with current knowledge.	P15L1-7
**Other**			
Source of funding	23	Describe how the study was funded and the role of the funder in the identification, design, conduct, and reporting of the analysis. Describe other non-monetary sources of support.	P15L10-12
Conflicts of interest	24	Describe any potential for conflict of interest of study contributors in accordance with journal policy. In the absence of a journal policy, we recommend authors comply with International Committee of Medical Journal Editors recommendations.	

For consistency, the CHEERS Statement checklist format is based on the format of the CONSORT statement checklist

The **ISPOR CHEERS Task Force Report** provides examples and further discussion of the 24-item CHEERS Checklist and the CHEERS Statement. It may be accessed via the *Value in Health* link or via the ISPOR Health Economic Evaluation Publication Guidelines – CHEERS: Good Reporting Practices webpage: http://www.ispor.org/TaskForces/EconomicPubGuidelines.asp

The citation for the CHEERS Task Force Report is:

Husereau D, Drummond M, Petrou S, et al. Consolidated health economic evaluation reporting standards (CHEERS)—Explanation and elaboration: A report of the ISPOR health economic evaluations publication guidelines good reporting practices task force. Value Health 2013;16:231-50.

For peer review only

BMJ Open

The short-term and long-term cost-effectiveness of a pedometer-based exercise intervention in primary care: A within-trial analysis and beyond-trial modelling

Journal:	*BMJ Open*
Manuscript ID	bmjopen-2018-021978.R1
Article Type:	Research
Date Submitted by the Author:	07-Jun-2018
Complete List of Authors:	Anokye, Nana; Brunel University, Health Economics Research Group<br>Fox-Rushby, Julia; Kings College London, School Population Health and Environmental Sciences<br>Sanghera, Sabina ; University of Bristol, School of Social and Community Medicine<br>Cook, Derek; St George's University, Division of Population Health Sciences<br>Limb, Elizabeth; St George's University, Division of Population Health Sciences<br>Furness, Cheryl; St George's University, Division of Population Health Sciences<br>Kerry, Sally; Queen Mary University of London<br>Victor, Christina; Brunel University, Social Work & Community/Public Health<br>Iliffe, Steve; University College London, Primary Care and Population Sciences<br>Ussher, Michael; St George's University of London, Division of Population Health Sciences and Education<br>Whincup, Peter; St Georges, University of London<br>Ekelund, Ulf; Norwegian School of Sport Sciences<br>DeWilde, Stephen; St George's University of London<br>Harris, Tess; St George's University of London
**Primary Subject Heading**:	Health economics
Secondary Subject Heading:	Public health, General practice / Family practice, Sports and exercise medicine
Keywords:	physical activity, cost-effectiveness, RCT, long-term modelling

SCHOLARONE™ Manuscripts

Full Title: The short-term and long-term cost-effectiveness of a pedometer-based exercise intervention in primary care: A within-trial analysis and beyond-trial modelling

Short title: Economic evaluation of pedometers delivered through primary care

Nana Anokye[1] PhD, JuliaFox-Rushby[2] PhD, SabinaSanghera[3] PhD, Derek G. Cook[4] PhD, Elizabeth Limb MSc[4], Cheryl Furness MSc[4], Sally Kerry PhD[5], Christina Victor PhD[6], Steve Iliffe FRCGP[7], Michael Ussher PhD[4], Peter H.Whincup PhD[4], Ulf Ekelund PhD[8,9], Steve DeWilde MD[4], Tess Harris MD[4]

[1]Health Economics Research Group, Brunel University London, Kingston Lane, Uxbridge, UB8 3PH, UK;
[2]Department of Population Health Sciences, Guys Campus, Kings College London, SE1 1UL
[3]School of Social & Community Medicine, University of Bristol, BS8 1TH
[4]Population Health Research Institute, St George's University of London, Cranmer Terrace, London, SW17 0RE, UK;
[5]Pragmatic Clinical Trials Unit, Queen Mary's University of London, 4 Newark Street, London, E1 2AT, UK;
[6]Department of Clinical Sciences, Brunel University London, Kingston Lane, Uxbridge, UB8 3PH, UK;
[7]Research Department of Primary Care & Population Health, University College London, St Dunstans Road, London, W6 6RP, UK;
[8]Department of Sport Medicine, Norwegian School of Sport Sciences, Postbox 5 Torshov, 0412 Oslo, Norway;
[9]MRC Epidemiology Unit, University of Cambridge, Cambridge Biomedical Campus, Cambridge, CB2 0QQ, UK;

Corresponding author
Professor Julia Fox-Rushby, Department of Population Health Sciences, Faculty of Medicine and Life Sciences, 5th Floor Addison House, Guys Campus, Kings College London, SE1 1UL
Email: Julia.Fox-Rushby@kcl.ac.uk Telephone 020 7848 8672
Orcid id: 0000-0003-0748-0871

For peer review only - http://bmjopen.bmj.com/site/about/guidelines.xhtml

Abstract

Objectives: A short- and long-term cost-effectiveness analysis (CEA) of two pedometer-based walking interventions compared with usual care

Design: a) Short-term CEA: parallel three-arm cluster randomised trial randomised by household b) Long-term CEA: Markov decision-model

Setting: Seven primary care practices in South London, United Kingdom

Participants: a) Short-term CEA: 1023 people (922 households) aged 45-75yrs without physical activity (PA) contraindications b) Long-term CEA: 100,000 cohort aged 59-88yrs

Interventions: Pedometers, 12-wk walking programmes, and PA diaries delivered by post or through three PA consultations with practice nurses

Primary and Secondary Outcome Measures: Accelerometer-measured change (baseline-12months) in average daily step-count and time in 10-min bouts of moderate-vigorous PA, and EQ5D5L quality-adjusted life-years (QALYs)

Methods: Resource use costs (£2013/4) from an NHS perspective, presented as incremental cost-effectiveness ratios for each outcome over a 1-year and life-time horizon, with cost-effectiveness acceptability curves and willingness to pay per QALY. Deterministic and probabilistic sensitivity analyses evaluate uncertainty.

Results: a) Short-term CEA: At 12months, incremental cost was £3.61(£109) per minute in ≥10 minute MVPA bouts for nurse-support compared with control (postal group). At £20,000/QALY, the postal group had a 50% chance of being cost-saving compared with control. b) Long-term CEA: The postal group had more QALYs (+759QALYs, 95% CI 400, 1247) and lower costs (-£11m, 95% CI -12,-10), than control and nurse groups, resulting in an incremental net monetary benefit of £26m per 100,000 population. Results were sensitive to reporting serious adverse events, excluding health service use, and including all participant costs.

Conclusions: Postal delivery of a pedometer intervention in primary care is cost-effective long-term and has a 50% chance of being cost-effective, through resource savings, within one year. Further research should ascertain maintenance of the higher levels of PA, and its impact on quality of life and health service use.

Trial Registration: ISRCTN98538934

For peer review only - http://bmjopen.bmj.com/site/about/guidelines.xhtml

Strengths and Limitations of this study

- This study provides the first primary data on the short-term costs associated with delivering pedometers to a large (n=1023), population-based, sample from primary care alongside a high quality randomised controlled trial that achieved a 93% follow-up rate at 12 months.
- Results from the trial are fed into a peer-reviewed, policy-relevant, Markov model to estimate long-term cost-effectiveness as trials of public health interventions are unable to reflect the balance of costs and effects when benefits occur in the long term.
- Results are tested in a number of sensitivity analyses to assess the impact of changing perspective, missing data, changes assumptions about maintenance of PA and of taking more conservative views of outcomes and cost impact.
- The main limitation of the economic analysis is the lack of information about the likelihood of maintaining PA beyond three years into the long term and the exclusion of long term impacts on other conditions e.g. cancers

For peer review only

Introduction

Increasing physical activity (PA) is a widely-stated policy aim from local to international level.[1,2] Walking is a safe and, potentially cheap, activity that has the potential to reduce cardiovascular disease, diabetes, cancer and poor mental health.[3] It is therefore important to establish which approaches are effective at: encouraging inactive people to do at least some walking; increasing the number of people walking briskly for at least 150 mins a week (i.e. achieving moderate-to-vigorous PA (MVPA) guidelines[2]); and/or maintaining increases in walking over time. This would also provide the basis for estimating cost-effectiveness and supporting recommendations for policy and practice.

Until recently, the best evidence of pedometer-based walking programmes was from systematic reviews that relied on small, short-term, studies where the independence of pedometer effects, from other support provided was unclear.[4] These had shown that walking interventions can achieve increases of ~2000-2500 steps/day at 3 months, but often relied on volunteer samples or high risk groups and did not assess time in MVPA, as defined in PA guidelines, as an outcome. New evidence from a large, randomised, trial clustered by household (PACE-UP) compared delivery of pedometers by post or through primary care nurse-supported PA consultations. The trial was undertaken with 1,023 inactive primary care patients aged 45-75 years from seven practices in south London. Results showed that step-counts increased by around 10% and time in MVPA in 10-minute bouts by around a third, with both the nurse and postal delivery arms achieving similar 12-month outcomes.[4] This is important because primary care can be a key to reaching directly into the community and offering continuity of care for increasing PA. It is shown that this type of intervention is suitable for older adults, where exercise referral schemes have been disappointing[4]. Compared with national averages (from Health Survey for England 2012 dataset) for the same age range of the PACE-UP trial, the trial sample were more overweight/obese (66% vs 61%), more likely to have/have had a higher managerial, administrative, professional occupation (59% vs 36%), and less likely to be white (80% vs 93%)..

Other than a small, highly selected, study which limited outcomes to steps achieved among 79 people from one family physician practice in Glasgow,[5] there is no primary evidence of the cost-effectiveness of pedometer programmes in the UK. Elsewhere, in Australia, New Zealand, and the Netherlands, economic models from community-based adults with low PA levels compare pedometer prescriptions and pedometer-based telephone

For peer review only - http://bmjopen.bmj.com/site/about/guidelines.xhtml

coaching with usual practice.[6–8] These indicate, pedometer-based interventions may be cost-effective in the long term, but estimates vary widely and generalisability is not considered.[9]

The analytic horizon of cost-effectiveness analyses should extend far enough into the future to capture all benefits and harms, although in practice this can be limited by the amount and quality of data.[10] NICE's public health guidance[11] also recommends providing results that reflect the short term (one to three years). This is reinforced in NICE's return on investment models,[12] which argue that shorter-term decision-making is of key interest to some decision-makers and which have been used by commissioners.

This paper estimates the short-term (one year) and long-term (life-time) cost-effectiveness of pedometers delivered by post or through practice nurse consultation for 1,023 inactive adults aged 45-75years. The short-term evaluation arises from a within-trial analysis of individual resource use and costs of interventions provided in the PACE-UP trial.[4] The cost and effectiveness results from the trial are used to populate a long-term model[13] for life-time cost-effectiveness.

Methods

Short-term cost-effectiveness

The short-term within-trial cost-effectiveness analysis was conducted alongside the PACE-UP trial[4,14] that evaluated two intervention groups against control (no intervention group). The two intervention groups received pedometers (SW-200 Yamax Digi-Walker) (one by post), patient handbook; PA diary (including individual 12-wk walking plan), with the nurse group also offered three individually tailored practice nurse PA (10- to 20-min) consultations (nurse-support group only) at approximately weeks 1, 5, and 9.[4] The control group followed usual practice and were not provided with any feedback on their PA levels or materials promoting PA during the trial.[4] These interventions could therefore evaluate the incremental effect of adding nurse support to pedometers.

The costs for the two intervention arms include set-up costs, staff training and intervention delivery (including; pedometers & clips, batteries, handbooks, diaries, postage, nurse time, time making appointments). Measures of each resource use were taken from administrative/trial management records, computer-based diaries, and interviews with the trial manager and principal investigator. To account for potential changes in falls, change in

For peer review only - http://bmjopen.bmj.com/site/about/guidelines.xhtml

use of health services following differential contact of health services by participants or unintended resources consequences, general health service use (eg general (family) physician visits, hospital admissions, accident and emergency attendances, referrals) was collected at participant level, through a one-time download of physician records at the end of the trial, and linked to procedure codes using PI judgement (blind to treatment group) to facilitate costing across elective and non-elective admissions. Information on costs borne by patients (eg time use, out of pocket expenses associated with walking groups, plus any related travel costs) was collected by questionnaire at 3 and 12 months. Resources were valued using national tariffs where possible[15,16] to increase generalisability; where not available tariffs from St Georges Hospital, London, were used. All costs are expressed in £2013-2014 sterling, inflated to this base year where appropriate using the Hospital & Community Health Service inflation index. As the trial lasted for one year, a discount rate was not applied. (See Supplementary File Tables S1-S5)

Outcomes were; (a) changes in daily steps and weekly minutes of MVPA in bouts of ≥10 mins, based on objectively measured PA by accelerometer and (b) changes in Quality Adjusted Life Years (QALYs), based on participant completion of the EQ-5D-5L questionnaires at baseline, 3 and 12 months. Utility weights were assigned using the ‘crosswalk’ function[17] linked to the standard UK-based weights[18], with QALYs based on the area under the curve.

Standard practice for accounting for missing data was followed. [19, 20] Patterns of missing data were investigated, with multiple imputation by chained equations fitted to replace item non-response. Missing EQ-5D data were replaced using an index rather than domain imputation as recommended[21]. Mean imputation was used where missing data was ≤5%[22]. Imputation models were fitted to match the model used for main analysis whilst including the predictors of missingness as appropriate. Second, the dependent variables were included in imputation models to ensure that the imputed values have similar relationships to the dependent variable as the observed values [23].

Results are reported, from an NHS perspective, as incremental cost-effectiveness ratios for cost per change in daily steps and cost per QALY for a one-year time-period, adjusted for baseline differences. A generalised linear model was fitted separately for costs and QALYs with clustered standard errors. To provide more precise estimates of uncertainty, the ‘margins method’ was used to generate sample means by trial arm for costs and

For peer review only - http://bmjopen.bmj.com/site/about/guidelines.xhtml

QALYs[24]. Cost models were fitted using the Poisson distribution and QALY models using the binomial 1 family, equivalent to beta regression[25]. The choice of distributional family for the models was based on the modified Park test and comparison of observed and predicted values. Covariates included baseline level (for the QALY-based models)[24], practice and variables found to be correlates of PA-related outcomes[26]- ie demography (age, gender, ethnicity, marital status, education, employment, socio economic status, cohabitation), health (number of disease conditions), and other lifestyle behaviours (smoking and alcohol intake). Reduced models were generated using Wald tests to examine the joint significance of variables found not to be significant (at 5%) in the base model.

Deterministic sensitivity analyses assessed: (a) inclusion of all randomised patients (rather than only those who provided accelerometry data); (b) exclusion of costs of general health service use beyond immediate intervention; (c) exclusion of missing data; (d) methods of accounting for adverse events; (e) perspective of analysis (ie including all and parts of participant costs); (f) varying the length of life of a pedometer; (g) the combination of excluding all health service use costs, and (h) including participant costs related to participation in physical activity and the interventions (minus health service use cost borne by participants, to ensure consistency in perspective). To reflect stochastic uncertainty surrounding mean incremental cost-effectiveness, cost-effectiveness planes (CEPs) and acceptability curves (CEACs) were constructed using 2000 non-parametric bootstrap samples from the base case estimates.

Long-term cost-effectiveness

A Markov model used to support NICE public health guidance[27] and return on investment modelling[12] was adapted to examine the long-term (life-time) cost effectiveness. From an NHS perspective, costs (2013/4 prices) and health outcomes from reduced disease, expressed as QALYs were discounted at the rate of 3.5% per annum. Results are reported as incremental cost-effectiveness ratios, cost-effectiveness acceptability curves and incremental net benefit statistics.

In the original model,[13] a cohort of 100,000 33 year-old people were followed in annual cycles over their life-time. At the end of the first year of the model, the cohort is either 'active' (doing 150 minutes of MVPA in 10 mins bouts per week) or 'inactive' and they could have one of 3 events (non-fatal CHD, non-fatal stroke, type 2 diabetes), remain event free (ie without CHD, stroke, or diabetes) or die either from CVD or non-CVD causes,

For peer review only - http://bmjopen.bmj.com/site/about/guidelines.xhtml

each of which had assigned annual treatment costs (split by initial event and follow-up). After the first year, people would revert to PA patterns observed in long-term cohort studies (up to 10 year cycle in the model) on the relationship between PA and disease conditions[13]. The key driver of the long-term model is the protective effects of PA, which is a function of PA patterns after the first year of the intervention. In the base case analysis, PA behaviour was based on PA patterns observed in long-term cohort studies[28–30] on the relationship between PA and disease conditions. The cohort studies used followed up the same people (who were either active or inactive at baseline) for 10 years, during which some of the inactive people might have become active or vice versa. Thus the impact of changing habits is incorporated in the cohort relative risk (RR) estimates from these epidemiological studies. However, assuming that these estimates would persist after the follow-up periods might be impractical. It was therefore assumed, conservatively, that these RR estimates held for an initial 10-year period (i.e. the period PA patterns were observed in the epidemiological studies), after which no protective benefit would persist. Hence, the RRs for developing CHD, stroke and T2D in the first 10 years of the model were based on the estimates from the epidemiological studies but from year 11 onwards they were assumed to be equal to 1 (no effect). This assumption was tested sensitivity analyses.

Active individuals had lower risks of developing CHD, stroke and type-2 diabetes. People who become active in the first year (irrespective of trial arm) also accrue short-term psychological benefits, a one-off utility gain associated with achieving the recommended level of physical activity[13] (see supplementary file Figure S1).

The model was adapted, using data from the PACE-UP trial, in the following ways:

a) a cohort of 100,000 people aged 59 years followed, in annual cycles, to 88 years, reflecting the average age of all trial participants at baseline and the average life expectancy for people aged 59 years in UK[31] and exposed, at this age, to interventions (either nurse or postal) in an unexposed population ie control group/usual care;

(b) age-specific estimates were revised to reflect the change in the cohort age,

(c) within-trial cost of interventions was used, with a second year of annuitized values included appropriately - postal (£5·03/person) and nurse group (£4·14/ person);

(d) effectiveness was reflected as the relative risk of achieving ≥150 MVPA mins per week in ≥10 minute bouts; and

For peer review only - http://bmjopen.bmj.com/site/about/guidelines.xhtml

(e) short-term psychological benefits of PA (one-off utility gain) estimated using beta regression fitted for EQ-5D scores at 12 months for active people controlling for EQ-5D scores at baseline, demographics, practice, disability and trial arm using.

All other parameters remained the same as the original model, based on literature reviews or evidence from national/international science-based guidance on PA and health. Parameter estimates are provided in supplementary file Table S6.

Deterministic sensitivity analysis explored four, conservative, scenarios: (1) assuming the protective effects of PA exist only for 1 year, as the trial MVPA data was assessed at 12 months; (2) assuming the protective effects of PA exist for 3 years. Recent evidence[32] relating to 3 year follow-up of participants of the interventions showed persistent effect at 3 years; (3) Exclusion of all health service use cost consequences during trial period (model year one) and assumed no psychological benefits in the first year of being physically active. This was considered due to the uncertainty around short term changes to health service use and because previous studies found the exclusion of short-term QALY gain associated with being physically active to affect conclusions[13]; (4) Scenario 3 plus all patient costs related to participation in physical activity and the interventions (details of the participants costs are provided in supplementary file Table S4). Probabilistic sensitivity analysis was based on 10,000 Monte Carlo simulations and included all parameters except baseline mortality, as the mortality census data has little uncertainty.

Results

Short-term cost-effectiveness

Table 1 summarises data on costs, EQ-5D-5L utility scores and QALYs by trial arm. At 3 months, average cost per participant was highest in the nurse group (£249) followed by the postal (£122) and control group (£107). In terms of the components of total costs, the cost of nurse-supported pedometer delivery was seven times greater (£50) than the postal group (£7), and set-up costs was double. Comparing the trial arms based on cost of health service use shows that the control group cost £35 more per participant than the postal group and £12 more than the nurse group. Results are similar at 12 months, except for the control arm, which has a higher overall average cost than the postal arm.

For peer review only - http://bmjopen.bmj.com/site/about/guidelines.xhtml

Table 2 shows that, at three months, mean incremental costs were significantly higher for the nurse group compared with the postal (+£120, 95% CI £95, £146) and control groups (+£135, 95% CI £99, £171) but not statistically significantly higher for the postal compared with control group. While increases in both daily steps and weekly minutes of MVPA in ≥10 minute bouts for both interventions compared with control, and for the nurse group compared with postal (nurse: +481steps (95% CI: 153, 809), +18mins MVPA (95% CI: 1, 35)) were statistically significant, the small mean decrease in QALYs is not statistically significant for any comparison. The cost per additional minute of MVPA was 35p for postal group and £2·21 for the nurse group and therefore the (slightly) fewer QALYs for both interventions compared with control contributed to the dominance of each intervention by the control group (ie the control group cost less and had more QALYs). To move from a postal to nurse delivered pedometer would cost 25p per additional step and £6·67 per additional MVPA minute. However, in terms of cost-effectiveness, the nurse group costs more and produces less QALYs on average than the postal group at 3 months.

Results differ at 12 months. Compared with the control group, the postal arm cost less on average (-£91) and the nurse group more (+£126) but neither are statistically significant. The increase in cost of moving from a postal to nurse delivery is also statistically significantly higher (+£217, CI £81, £354). While both interventions are associated with a statistically significant increase in steps and weekly mins of MVPA, the difference between intervention groups is not statistically significant at 12 months. The small decrements in QALYs at each incremental comparison are not statistically different. The postal group took more steps (+642) and cost less on average (-£91) compared with control and dominates control in terms of PA outcomes. The nurse group cost 19p per additional step and £3.61 per additional minute of MVPA compared with control, with this rising to £6 and £109 respectively when compared with the postal group. In terms of QALYs, the nurse group is still dominated (ie cost more and had worse outcomes) by the control and postal groups. However, on average, each QALY lost in the postal group compared with control is associated with a saving of £21,162, which could therefore be considered cost-effective.

The probabilistic sensitivity analyses broadly confirm the findings of the base case; the postal group is most often associated with lower QALYs along with cost savings and the nurse group tends to have both lower QALYs and higher costs compared with control and postal group (Supplementary file, Figs S2-S4). Figure 1 shows that at £20,000 per QALY gained/lost, the postal group has a 50% chance of being cost-effective

For peer review only - http://bmjopen.bmj.com/site/about/guidelines.xhtml

compared with control (usual care). This falls to 42% at £30,000/QALY, which reflects the postal group having most observations in the lower left hand quadrant (as seen in Supplementary file, Fig S2). Figure 1 also shows that, at a willingness to pay/lose a QALY of £20,000, the nurse group has a 5.5% chance of being cost-effective compared with control.

The deterministic sensitivity analyses (Supplementary File, Table S7) mostly produced results consistent with the base case findings. However, in four circumstances, usual care would dominate both the postal and nurse groups at 12 months; i) using health service use based on self-reported serious adverse effects; ii) excluding all health service costs; iii) changing perspective (including all participant costs); and iv) the worst-case 'combined scenario' sensitivity analyses.

Long-term cost-effectiveness

Table 3 shows that, over the remaining life-time from age 59, the nurse group would be costlier (£11m, 95% CI: £10m, £12m) but have more QALYs (671 95% CI: 346, 1071) per 100,000 population than the control group and therefore provide each additional QALY at a cost of £16,368. However, the postal group would have lower life-time costs than the control arm (-£11m per 100,000 population, 95% CI: £-12m, £-10m) and more QALYs (759, CI: 400, 1247) it is therefore the dominant option, with an incremental net benefit of £26million per 100,000 population (95% CI: £18m, £36m). These results are confirmed by the incremental net benefit, which shows the £2m per 100,000 for nurse group compared with control is not significantly different and compared with the post group is significantly negative (-£24m 95% CI: -£27, -£21).

The stochastic uncertainty associated with the mean incremental cost-effectiveness ratio (ICER) (Figure 2) indicates the above findings are robust. There is a 100% likelihood, at a willingness to pay of £20,000/QALY, that the postal group is cost-effective compared with the control and nurse groups. This is consistent with the estimates of net monetary benefit in Table 3. At £20,000/QALY, there is a 70% likelihood that the nurse group would be cost-effective compared with control (Figure 2).

The results for the sensitivity analyses were:

For peer review only - http://bmjopen.bmj.com/site/about/guidelines.xhtml

(a) Scenario 1 - (i) postal vs control: postal remained dominant, less expensive (-£9m) with more QALY gains (+211QALYs); (ii) Nurse vs control: The ICER further increased from £16,000 to £69,000 (+£12.8m, +186QALYs); (iii) Nurse vs postal: The Nurse group remained dominated by postal group (+£21.6m, -32QALYs).

(b) Scenario 2 - (i) postal vs control: postal was still dominant, less expensive (-£9.2m) with more QALY gains (+327QALYs); (ii) Nurse vs control: The ICER increased from £16,000 to £43,000 (+£12.4m, +289QALYs); (iii) Nurse vs postal: The Nurse group remained dominated by postal group (+£21.7m, -48QALYs).

(c) Scenario 3 - (i) postal vs control: postal moved from a dominant position to a more expensive option (+£4m) with more QALY gains (+609QALYs), and an ICER of £6,100; (ii) Nurse vs control: The ICER increased from £16,000 to £26,000 (+£14m, +538QALYs); (iii) Nurse vs postal: The Nurse group remained dominated by postal group (+£10m, -87QALYs).

(d) Scenario 4 - (i) postal vs control: postal moved from a dominant position to more expensive (+£16m) and more QALY gains (+609 QALYs) with an ICER of £26,600; (ii) Nurse vs control: The ICER increased from £16,000 to £25,400 (+£13.7m, +538QALYs); (iii) Nurse vs postal: Nurse moved from dominated position (where costs are higher and QALYs lower to a cost-effective position (where both costs and QALYs are lower) (-£2m, -87QALYs).

Discussion

The life-time cost-effectiveness of posting a pedometer with written instructions to a cohort of 100,000 insufficiently active people aged 59 years (who have indicated an interest in research or participation in walking) would cost less (-£11m, 95%CI -12,-10) and provide more QALYs (759 QALYs, 95%CI 400, 1247) than usual care. Most cost-savings and quality of life benefits derive from reductions in stroke, CHD and type-2 diabetes. This finding was robust (incremental net benefit of £26m, 95%CI £18m, £36m) and sensitivity analyses showed that even excluding short-term cost savings would not change the conclusion that the postal group would be extremely cost-effective in the long-term (ICER: £6,100/QALY). Sending a pedometer by post with instructions from a primary care provider to inactive people aged 45-75 also has a 50% chance of being cost-effective *within a year*, as a 1 QALY loss was associated with saving over £21,000. The nurse group had higher costs and lower QALYs than both control and postal groups at 1 year. While sensitivity analyses did not

For peer review only - http://bmjopen.bmj.com/site/about/guidelines.xhtml

change conclusions in most cases, in three cases (using self-reported serious adverse events, excluding health service use, including all participant costs) it did, indicating that the control group would dominate (ie have lower costs and more QALYs) than both the postal and nurse groups.

A key strength of this study is the base of individualised cost and effectiveness data on a large, population-based, cluster-randomised, controlled trial with excellent follow-up data to one year (93.4%, Harris et al 2017)[4], designed to produce generalisable results, for cost per QALY estimates at one year and as inputs to a long-term model of cost-effectiveness. It is also the only study to have included provider and user perspectives, extended commonly used techniques to account for clustering and used conservative assumptions for both short- and long-term sensitivity analyses.

One weakness of the within-trial cost-effectiveness study concerns the use of PI judgement to determine costs of admissions, and therefore alternative assumptions were explored in sensitivity analyses. Patient reported cost data were collected for months 1-3 and 9-12, with the last 3 months multiplied to represent costs across all months from 4-12. If significantly underestimated, this could be decisional. To date, there are no primary economic data beyond 12 months of an intervention and very few trials include measures of quality of life measures alongside PA. Therefore, with respect to the long-term modelling, a key gap in knowledge is the likelihood of maintaining PA beyond 12 months. This model assumes differences in PA at 1 year in the trial relate to the same long-term benefit associated with the same difference in cohort studies, but this could be updated once longer-term follow-up data become available. Other challenges set out in Anokye et al 2014[13] are relevant here eg cancer and adverse events are not accounted for, which could lead to over or under-estimation of cost-effectiveness. Other challenges relate to the generalisability of effectiveness data, given the focus on South London and 10% recruitment rate, even though recruitment was comparable with other PA trials [33,34]. The trial was shown to recruit fewer: men, people aged 55-64yrs compared with those over 65yrs, people from the most deprived quintile compared with least deprived, and Asian compared with white people[35.]. However, there was good representation of women, older adults and people who were overweight, all of whom are groups likely to benefit from the intervention[4]. Investigation into the reasons for non-participation showed an important minority cited existing medical conditions, too many other commitments or considered themselves sufficiently active[35, 36].

For peer review only - http://bmjopen.bmj.com/site/about/guidelines.xhtml

.

This study feeds into an area with very limited primary data[37,38] populated only by small studies[5,6]. In New Zealand, pedometers were shown to have a 95% probability of being a cost-effective addition to green prescriptions at 12 months[5], much higher than the 50% likelihood we found. Other models of long-term cost-effectiveness studies identified cost savings and improved quality of life at a population level from pedometers in the long term[8,39] or indicated high probabilities of long-term cost-[7,40]. Guidance has also suggested that long-term monitoring/support at £25/year would be very cost-effective. Our study provides further support that pedometer-based programmes are a cost-effective method of improving health-related quality of life in both the short and long-term. Assumptions about intervention effectiveness beyond one year has mixed impacts, and further research is required to better judge whether existing models over- or under-predict cost-effectiveness.

Current public health guidance from NICE on pedometers[41] advises using pedometers as "part of a package which includes support to set realistic goals in one to one meetings (whereby the number of steps taken is gradually increased), monitoring and feedback. Our results not only provide substantially better economic data for use by NICE but also suggest guidance should be updated to reflect the value of providing pedometers, to people who have made some form of commitment (ie to a trial), through the post. For those practices that have implemented consultation-based distribution of pedometers, moving to postal delivery could save costs within a year, with similar outcomes.

Postal delivery of pedometer interventions to inactive people aged 45-75 through primary care is cost-effective in the long-term and has a 50% chance of being cost-effective, through resource savings, within one year. Further research is needed to ascertain the extent to which higher PA levels are maintained beyond three years and the impact of PA on quality of life and general health service use in both the short and long-term.

For peer review only - http://bmjopen.bmj.com/site/about/guidelines.xhtml

Supporting Information

Supplementary file 1: Copy of open access protocol for trial

Supplementary file 2: Figures 1 & 2

Supplementary file 3: Document for online supplement giving fuller study details

Supplementary file 4: CHEERS reporting statement

Author Contributions

Contributors: JFR conceived of the economic analysis, was co-applicant for funding, jointly designed the economic data collections tools, wrote the economic analysis plan, collected part of the data, supervised the economics, and jointly drafted and amended the script. She is guarantor for this script. NA jointly designed the data collection tools, cleaned and analysed the economic data, and jointly drafted and amended the script. SS collated and analysed the hospital cost data, commented on drafts and reviewed the final script. DC, EL and SK designed data collection for and analysed the intermediate outcome data underpinning the economic analyses, discussed plans and results as presented through the trial, commented on drafts of this manuscript and reviewed the final script. CF collated and provided access to the administrative data used for the economic analysis, was the research project administrator, commented on drafts and reviewed the final script. TH was the principal investigator, involved at all points of the planning, progress and review of the economic evaluation including commenting on drafts and review of the final script. She is guarantor for the whole trial. CRV, PHW, MU, SI, UE,SdW all conceived the trial plan and applied for funding, they contributed to conceptualisation of the economics within the broader context of the trial, discussed plans and results as presented through the trial, commented on drafts of this manuscript and revised the final script.

Acknowledgements

We thank Iain Carey for help with processing the primary care data as well as Abbie Hill and Julie Whittaker who ably supported administration of the economics project at Brunel University London. We remember and

For peer review only - http://bmjopen.bmj.com/site/about/guidelines.xhtml

thank too, our colleague and co-investigator, Dr S M Shah who died in September, 2015. We would like to thank the South-West London (UK) general practices, their practice nurses who supported this study, and all the patients from these practices who participated: Upper Tooting Road Practice, Tooting; Chatfield Practice, Battersea; Wrythe Green Practice, Carshalton; Francis Grove Practice, Wimbledon; Putneymead Practice, Putney; Heathfield Practice, Putney; and Cricket Green Practice, Mitcham. We would also like to thank our supportive Trial Steering Committee: Professor Sarah Lewis (chair); Professor Paul Little (GP representative); Mr Bob Laventure (Patient and Public Involvement representative).

For peer review only

Funding Statement

This research was supported by the Health Technology Assessment (HTA) Programme, National Institute for Health Research (project number HTA 10/32/02 ISRCTN42122561), The funding organisation played no role in the study design, collection, analysis and interpretation of the data, writing or decision to submit the paper for publication The views and opinions expressed therein are those of the authors and do not necessarily reflect those of the Health Technology Assessment (HTA) Programme, National Institute for Health Research, National Health Service or the Department of Health.

Competing Interests

JFR reports Board Membership of the Public Health Research Funding Board of NIHR. TH sits on Primary Care Community Interventions Panel for the Health Technology Assessment Programme of NIHR. All authors have been contracted to evaluate other public health interventions, including pedometers, for the NIHR.

Patient Consent

No individual can be identified from the work presented in this paper.

Data Sharing

Data is available upon request from Dr Tess Harris

For peer review only

References

1. Lichfield District Council (2016) Physical Activity and Sport Strategy 2016-2020 Available from: https://www.lichfielddc.gov.uk/Residents/Leisure-and-culture/Leisure-centres/Downloads/Lichfield-District-Physical-Activity-and-Sport-Strategy.pdf. (downloaded 21.02.201)

2. World Health Organisation (2010) Global recommendations on physical activity for health http://www.who.int/dietphysicalactivity/publications/9789241599979/en/

3. Kyu H, Bachman V, Alexander L, Mumford J, Afshin A, Estep K, Veerman J, et al. Physical activity and risk of breast cancer, colon cancer, diabetes, ischemic heart disease, and ischemic stroke events: systematic review and dose-response meta-analysis for the Global Burden of Disease Study 2013BMJ 2016;354:i3857

4. Harris T, Kerry SM, Limb ES, Victor CR, Iliffe S, Ussher M et al. Effect of a Primary Care Walking Intervention with and without Nurse Support on Physical Activity Levels in 45- to 75-Year-Olds:The Pedometer And Consultation Evaluation (PACE-UP) Cluster Randomised Clinical Trial. Plos Med 2017 http://dx.doi.org/10.1371/journal.pmed.1002210

5. Shaw R, Fenwick E, Baker G, McAdam C, Fitzsimons C, Mutrie N. Pedometers cost buttons: the feasibility of implementing a pedometer based walking programme within the community. *BMC Public Health* 2011, **11**:200

6. Leung W, Ashton T, Kolt G, Schofield G, Garrett N, Kerse N, et al.Cost-effectiveness of pedometer-based versus time-based Green Prescriptions: the Healthy Steps Study Australian *Journal of Primary Health*, 2012, **18**: 204–211

7. Over E, Wendel-Vos G, van den Berg M, Hamberg-van Reenen H, Tariq L, Hoogenveen R, van Baal P. Cost-effectiveness of counseling and pedometer use to increase physical activity in the Netherlands: a modeling study. Cost Effectiveness and Resource Allocation, 10:13

8. Cobiac LJ, Vos T, Barendregt JJ. Cost-effectiveness of interventions to promote physical activity: a modelling study. *PLOS medicine* 2009 https://doi.org/10.1371/journal.pmed.1000110

9. Vijay G, Wilson E, Suhrcke M, Hardeman W, Sutton S, on behalf of the VBI Programme Team . Are brief interventions to increase physical activity cost-effective? A systematic review Br J *Sports Med* 2015; **0**:1–11

10. Honeycutt A, Clayton L, Khavjou O, Finkelstein E, Prabhu M, Blitstein J et al. Guide to Analyzing the Cost-Effectiveness of Community Public Health Prevention Approaches Report to U.S. Department of Health and Human Services RTI Project Number 0208827.001. 2006. Available from: https://aspe.hhs.gov/system/files/pdf/74686/report.pdf

11. National Institute for Health and Care Excellence (2014) Developing NICE guidelines: the manual. Available from: https://www.nice.org.uk/process/pmg20/chapter/incorporating-economic-evaluation

12. Matrix. Estimating Return on Investment for interventions and strategies to increase physical activity London: Matrix Knowledge, 2014.

13. Anokye, NK., Lord, J., Fox-Rushby, J., Is brief advice in primary care a cost-effective way to promote physical activity?, *British Journal of Sports Medicine* 2014, **48 (3)** : 202–206

14. Harris T, Kerry SM, Victor CR, Shah SM, Iliffe S, Ussher M, Ekelund U et al. PACE-UP (Pedometer and consultation evaluation - UP) - a pedometer-based walking intervention with and without practice nurse support in primary care patients aged 45-75 years: study protocol for a randomised controlled trial. Trials 2013;**14**:418.

15. Department of Health (2015). NHS Reference Costs 2013 to 2014. Available from:https://www.gov.uk/government/publications/nhs-reference-costs-2013-to-2014

For peer review only - http://bmjopen.bmj.com/site/about/guidelines.xhtml

16. Curtis LA: Unit costs for health and social care Personal Social Services Research Unit, University of Kent: Canterbury; 2014

17. EuroQol Group (2013) EuroQOl EQ5D User Guide v2.0 (downloaded June 2013)

18. Dolan P: Modeling valuations for EuroQol health states. *Medical Care* 1997 Nov;**35**(11):1095-108

19. Allison PD. Multiple Imputation for Missing Data: A Cautionary Tale. Social Methods Research. 2000;28:301-9.

20. Carpenter JR, Kenward, M.G. MAR methods for quantitative data. In: Missing data in randomised controlled trials— a practical guide. National Institute for Health Research. 2008.

21. Simons CL, Rivero-Arias O, Yu LM, Simon J. Multiple imputation to deal with missing EQ-5D-3L data: Should we impute individual domains or the actual index? *Qual Life Res* 2014; DOI 10.1007/s11136-014-0837

22. Schafer, J. L. Multiple imputation: A primer. Statistical Methods in Medical Research; 2009, **8**:3–15.

23. Sterne JA, White IR, Carlin JB, Spratt M, Royston P, Kenward MG, et al. Multiple imputation for missing data in epidemiological and clinical research: potential and pitfalls. *BMJ*. 2009;338:b2393.

24. Glick HA, Doshi JA, Sonnad SS, Polsky D. Economic evaluation in clinical trials. (2 ed.) Oxford University Press 2014

25. Basu A, Manca A. Regression estimators for generic health-related quality of life and quality adjusted life years. *Med Decision Making* 2012; **32**: 56–69

26. Smith L, Gardner B, Fisher A, et al. (2014) Patterns and correlates of physical activity behaviour over 10 years in older adults: prospective analyses from the English Longitudinal Study of Ageing. *BMJ Open* 2015;5:e007423. doi:10.1136/bmjopen-2014- 007423

27. Public Health Guidance [PH44] Physical activity: brief advice for adults in primary care National Institute for Health and Care Excellence.

28. Hu G**,** Qiao Q**,** Silventoinen K, Eriksson JG, Jousilahti P, Lindstrom J et al**,** Occupational, commuting, and leisure-time physical activity in relation to risk for Type 2 diabetes in middle-aged Finnish men and women. *Diabetologia* 2003; 46:322–329

29. Hu G, Sarti C, Jousilahti P, Silventoinen K, Barengo NC, Jaakko Tuomilehto Leisure Time, Occupational, and Commuting Physical Activity and the Risk of Stroke. *Stroke.* 2005; 36:1994-1999.

30. Hu G, Jousilahti P, Borodulin K, Barengo NC, Lakka TA, Nissinen A et al. Occupational, commuting and leisure-time physical activity in relation to coronary heart disease among middle-aged Finnish men and women.*Atherosclerosis* 2007;194:490-7

31. Office for National Statistics (ONS). Life Expectancy. ONS Digital 2015 (Accessed August 2015) Available from: http://visual.ons.gov.uk/how-long-will-my-pension-need-to-last/)

32. Harris T, Kerry SM, Limb ES et al. Physical activity levels in adults and older adults 3-4 years after pedometer-based walking interventions: Long-term follow-up of participants from two randomised controlled trials in UK primary care. *Plos Med* 2018 https://doi.org/10.1371/journal.pmed.1002526

33. Tully MA, Cupples ME, Chan WS, McGlade K, Young IS. Brisk walking, fitness, and cardiovascular risk: a randomized controlled trial in primary care. *Prev Med*. 2005;41(2):622-8.

For peer review only - http://bmjopen.bmj.com/site/about/guidelines.xhtml

34. McMurdo ME, Sugden J, Argo I, Boyle P, Johnston DW, Sniehotta FF, et al. Do pedometers increase physical activity in sedentary older women? A randomized controlled trial. *J Am Geriatr Soc*. 2010;58(11):2099-106

35. Kerry S, Morgan K, Limb E, Cook D, Furness C, Carey I, DeWilde S, Victor C, Whincup P, Ussher M, Ekelund U, Fox-Rushby J, Ibison J, Harris T., (2018) Interpreting population reach of a large, successful physical activity trial delivered through primary care *BMC Public Health* BMC Public Health **1**8:170

36. Normansell R, Holmes R, Victor C, Cook DG, Kerry S, Iliffe S, Ussher M, Fox-Rushby J, Whincup P, Harris T (2016) Exploring non-participation in primary care physical activity interventions: PACE-UP trial interview findings *Trials* 17:178

37. Laine J, Kuvaja-Kollner V, Pietila E, Koivuneva M, Valtonen H, Kankaanpdd E. Cost-Effectiveness of Population-Level Physical Activity Interventions: A Systematic Review *American Journal of Health Promotion* November/December 2014, **29(2):** 71–80

38. Garrett S, Raina Elley C, Rose SB. Are physical activity interventions in primary care and the community cost-effective? A systematic review of the evidence. *British Journal of General Practice* 2011; 61(584):e125-e133

39. de Smedt D, de Cocker K, Annemans L, de Bourdeaudhuij I, Cardon G. (2012) A cost-effectiveness study of the community-based intervention '10000 Steps Ghent' *Public Health Nutrition* **15(3):**442–51

40. Brennan A, Blake L, Hill-McManus D, Payne N, Buckley Woods H, Blank L. Walking and cycling: local measures to promote walking and cycling as forms of travel or recreation: Health economic and modelling report, National Institute for Health and Clinical Excellence Public Health Programme; 2012. Available from: https://www.nice.org.uk/guidance/ph41/evidence/economic-modelling-report-430262893

41. National Institute for Health and Care Excellence (NICE), Public Health Guidance [PH41] Physical activity: walking and cycling (2012). Available from: https://www.nice.org.uk/guidance/ph41/resources/physical-activity-walking-and-cycling-pdf-1996352901061

For peer review only - http://bmjopen.bmj.com/site/about/guidelines.xhtml

Table 1: Average costs and QALYs per participant, by trial arm, (£'sterling2013/14, all randomised participants who provided required accelerometry data*, missing data imputed)

Cost and quality of life (EQ5D5L)	Control	Postal	Nurse
	Mean (SD)		
**0-3 months**	**n=318**	**n=317**	**n=319**
Total cost	*£107 (254)*	*£122(107)*	*£249 (215)*
Set up	£0 (0)	£45(0)	£105(0)
Delivery of intervention	£0 (0)	£7 (0)	£50 (18)
Health service use	£107(254)	£71(107)	£95 (214)
EQ 5D scores at baseline	0.839 (0.14)	0.853 (0.12)	0.851 (0.12)
EQ 5D scores at 3 months	0.844 (0.14)	0.848 (0.14)	0.841 (0.14)
QALYs 0-3 months	0.194 (0.03)	0.196 (0.03)	0.195 (0.03)
**0-12 months**	**n=323**	**n=312**	**n=321**
Total cost	*£461 (916)*	*£375(611)*	*£603 (987)*
Set up	£0 (0)	£45 (0)	£105 (0)
Delivery of intervention	£0 (0)	£10 (0)	£52 (18)
Health service use	£461 (916)	£320 (611)	£447 (987)
EQ 5D scores at baseline	0.837 (0.14)	0.850 (0.12)	0.849 (0.13)
EQ 5D scores at 3 months	0.840 (0.14)	0.847 (0.13)	0.837 (0.14)
EQ 5D scores at 12 months	0.833 (0.15)	0.836 (0.13)	0.831 (0.14)
QALYs 0-12 months	0.837 (0.13)	0.843 (0.11)	0.836 (0.13)

*The number of people who provided accelerometry data differed across time points within arms * For incremental analyses, the comparisons are postal vs control and nurse vs control

For peer review only - http://bmjopen.bmj.com/site/about/guidelines.xhtml

Table 2: Regression estimates for costs, effects and cost-effectiveness at 3 and 12 months(£'sterling 2013/14) (base case, adjusted for baseline differences)

Cost, effects or cost-effectiveness		Control		Postal*		Nurse *		Nurse vs Postal	
		Mean	(95% CI)	Mean	(95% CI)	Mean	(95% CI)	Mean	(95% CI)
Costs and effects over 3 months	Total cost per participant (£)	108	(80 to 136)	123	(111 to135)	244	(221 to 266)	-	
	Incremental cost (£)	-		15	(-15 to 45)	135	(99 to 171)	120	(95 to 146)
	Total QALYs per participant	0.1957	(0.1936 to 0.1978)	0.1952	(0.1930 to 0.1974)	0.1948	(0.1926 to 0.1970)	-	
	Incremental* QALYs	-		-0.0005	(-0.0027 to 0.0016)	-0.0009	(-0.0031 to 0.0012)	-0.0004	(-0.0026 to 0.0018)
	Incremental daily steps			692	(363 to 1020)	1172	(844 to 1501)	481	(153 to 809)
	Incremental weekly mins of MVPA in bouts of ≥10 mins			43	(26 to 60)	61	(44 to 78)	18	(1 to 35)
Costs and effects over 12 months	Total cost per participant (£)	467	(365 to 569)	376	(307 to 445)	593	(473 to 714)	-	
	Incremental cost (£)	-		-91	(-215 to 33)	126	(-37 to 290)	217	(81 to 354)
	Total QALYs per participant	0.842	(0.832 to 0.853)	0.838	(0.827 to 0.849)	0.836	(0.824 to 0.847)	-	
	Incremental QALYs	-		-0.004	(-0.017 to 0.009)	-0.007	(-0.020 to 0.007)	-0.002	(-0.016 to 0.011)
	Incremental daily steps	-		642	(329 to 955)	677	(365 to 989)	36	(-227 to 349)
	Incremental weekly mins of MVPA in bouts of ≥10 mins	-		33	(17 to 49)	35	(19 to 51)	2	(-14 to 17)
IC	Cost per additional QALY (£)	-		*Postal dominated by control*		*Nurse dominated by control*		*Nurse dominated by Postal*	

For peer review only

For peer review only - http://bmjopen.bmj.com/site/about/guidelines.xhtml

Cost, effects or cost-effectiveness		Control		Postal*		Nurse*		Nurse vs Postal	
		Mean	(95% CI)	Mean	(95% CI)	Mean	(95% CI)	Mean	(95% CI)
	Cost per additional step count (£)	-		£0.02		£0.12		£0.25	
	Cost per additional minute of MVPA in a bout of ≥10 mins (£)	£0.35		£0.35		£2.21		£6.67	
ICER* at 12 months	Cost per additional QALY (£)	-		*Postal is less costly but has fewer QALYs. £21,162 saved per QALY lost*		*Nurse dominated by control*		*Nurse dominated by Postal*	
	Cost per additional step count (£)	-		*Postal dominates control*		0.19		6.03	
	Cost per additional minute of MVPA in a bout of ≥10 mins (£)	-		*Postal dominates control*		3.61		109.00	

For peer review only - http://bmjopen.bmj.com/site/about/guidelines.xhtml

Table 3: Costs, effects and cost-effectiveness over a lifetime from age 59 (100,000 cohort)

	Control	Postal*	Nurse *	Nurse vs Postal
	**Mean (95% CI)**	**Mean (95% CI)**	**Mean (95% CI)**	**Mean (95% CI)**
Lifetime total cost (£million) **	340 (307, 371)	329 (296, 361)	351 (318, 384)	-
Lifetime incremental cost (£million)	-	-11 (-12, -10)	11 (10, 12)	22 (21 to 23)
Lifetime total QALYs (million)	1.0709 (0.879, 1.273)	1.0717 (0.889, 1.274)	1.0716 (0.880, 1.273)	-
Lifetime incremental QALYs	-	759 (400, 1247)	671 (346, 1071)	-108 (-223 to -10)
Lifetime ICER for QALYs (£)		Postal dominates control	16,368	Postal dominates nurse
Lifetime Incremental Net Monetary Benefit (£million, @ £20,000 per QALY)	-	26 (18, 36)	2 (-5, 11)	-24 (-27 to -21)

* For incremental analyses, the comparisons are postal vs control and nurse vs control.

**£46.7m, £37.6m and £59.3m of the total costs for control, postal and nurse groups respectively, were estimated using PACE-UP trial results

For peer review only

Figure Legends

Figure 1: Cost-effectiveness acceptability curve showing the probability of short-term (at 1 year) cost-effectiveness for postal and nurse groups (vs control) at different willingness to pay per QALY thresholds.

Figure 2: Cost-effectiveness acceptability curve showing the probability of life-time cost-effectiveness for postal and nurse groups (vs control) at different willingness to pay per QALY threshold levels

For peer review only

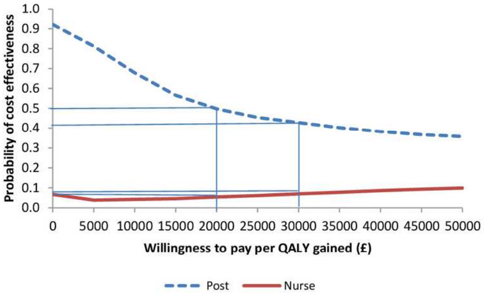

Figure 1: Cost-effectiveness acceptability curve showing the probability of short-term (at 1 year) cost-effectiveness for postal and nurse groups (vs control) at different willingness to pay per QALY thresholds.

140x198mm (300 x 300 DPI)

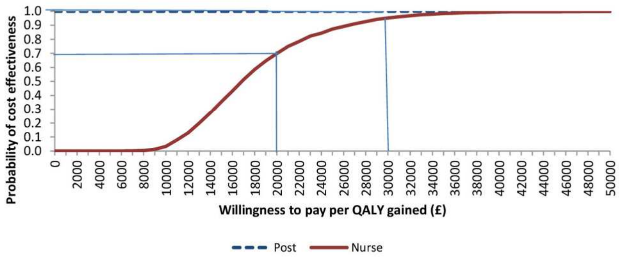

Figure 2: Cost-effectiveness acceptability curve showing the probability of life-time cost-effectiveness for postal and nurse groups (vs control) at different willingness to pay per QALY threshold levels

140x198mm (300 x 300 DPI)

Supplementary file for

"The short-term and long-term cost-effectiveness of a pedometer-based intervention in primary care: A within-trial analysis and beyond-trial modelling"

Nana Anokye PhD, Julia Fox-Rushby PhD, Sabina Sanghera PhD, Derek G. Cook PhD, Elizabeth Limb MSc, Cheryl Furness MSc, Sally Kerry PhD, Christina Victor PhD, Steve Iliffe FRCGP, Michael Ussher PhD, Peter H.Whincup PhD, Ulf Ekelund PhD, Steve DeWilde PhD, Tess Harris MD

For peer review only

Table S1: Resource use and cost components of 'Set-up Cost'*

**Activity (trial arm applicable to)**	**Resource**	**Total quantity**	**Cost per participant £ (nurse group)**	**Cost per participant £ (post group)**
**Design^**				
Designing of intervention (Both intervention arms)	Professor x1	0·5 days	4·43	4·43
	Readers x3	1 day		
	Senior lecturers x3	3·5 days		
	Consultants x2	1 days		
Designing of participants' handbooks and diaries (both intervention groups)	Professor x3	1·5 days	3·56	3·56
	Readers x2	1 day		
	Senior lecturers x3	2 days		
	Consultants x2	0·5 days		
Designing of nurse trainers handbooks (Nurse group)	Senior lecturers x1	1 day	2·74	0
	Consultants x1	0·5 days		
	Handbooks	9 handbooks	0·19	0
**Setting up GP practices**				
Planning for recruitment of practices (All trial arms)	Professor x1	1 hour	0·99	0·99
	Senior lecturer x1	5 hours		
	Consultants x2	5 hours		
Visits to recruit 6 practices (All trial arms)	Senior lecturers x2	13 hours	1·47	1·47
	Trial Manager x1	7 hours		
	Consultant x1	5 hours		
	Round trips to practices (by all)	25 hours	0·10	0·10
Searching practice computers to identify participants (All trial arms)	Senior lecturer x1	6 hours	0·71	0·71
	Trial Manager x1	6 hours		
	Practice Manager x6	6 hours		
Identify households from anonymised address list (All trial arms)	Senior lecturer x1	32 hours	2·28	2·28
	Trial Manager x1	32 hours		
Practice staff reviews lists for exclusion (All trial arms)	GP x5 (for sorting out 2 practices)	20 hours	4·50	4·0

Activity (trial arm applicable to)	Resource	Total quantity	Cost per participant £ (nurse group)	Cost per participant £ (post group)
	Nurse x10 (for sorting out other 5 practices)	50 hours	1·96	1·96
Printing letters at practice (All trial arms)	Trial Manager x1	64 hours	1·57	1·57
	Practice administrative staff x2	4 hours		
	Number of printed letters	24000	0·94	0·94
Packing envelopes with leaflets and letters (All trial arms)	Trial Manager x1	240 hours	7·04	7·04
	Research Assistants x2	56 hours		
	Practice admin. Staff x11	27·5 hours		
	Cost of Envelopes	£497·30	0·49	0·49
	Cost of Postal stamps	£5,530·50	5·41	5·41
	Cost of Information leaflets	£5,973·00	5·84	5·84
Preparing rooms at practices for trial (All trial arms)	Round trip to practices by RA	14 trips	0·04	0·04
	Research Assistants x2	-*	0·11	0·11
**Training**				
Training of Trial manager (All trial arms)	Trial Manager x1	4 days	1·51	1·51
	Senior lecturer x1	2 days		
Preparation of nurse training course (Nurse support group)	Trial Manager x1	1 day	9·63	0
	Senior lecturer x1	2 days		
	Reader x1	0·5 days		
	Consultants x2	2 days		
Mini-training day of nurses (Nurse group)	Nurses x11	33 hours	7·46	0
	Trial Manager x1	17·33 hours		
	Senior lecturer x1	17·33 hours		
	Round trips to training centre (by tutors)	16 hours	0·19	0
	Pedometers given to nurses	12 hours	0·04	0
Full training day of nurses (Nurse group)	Nurses x10	107·5 hours	22·99	0
	Reader x1	1 hour		
	Senior lecturer x1	10 hours		
	Consultants x2	22·5 hours		
	Round trips for training by nurses x10	10 trips	0·12	0
	Round trips for training by consultants x2	2 trips	0·13	0
	Refreshments	1 set	0·26	0
Training for an absentee nurse (Nurse group)	Nurse x 1	10 hours	2·47	0
	Trial Manager x1	11·33 hours		
	Research assistant x1	11·33 hours		
	Round trips to training centre	2 trips	0·02	0
Discussion of nurses recorded sessions(Nurse group)	Senior lecturer x1	0·5 days	3·78	0
	Consultants x2	1 day		
	Nurses x9	4·5	0·99	0
	Senior lecturer x1	0·5		

For peer review only

Activity (trial arm applicable to)	Resource	Total quantity	Cost per participant £ (nurse group)	Cost per participant £ (post group)
	Consultants x2	1		
	Duration of phone calls	270 mins	0·09	0
Follow-up half day training(Nurse group)	Nurses x 9	4.5 days	7·70	0
	Trial Manager x1	0·5 days		
	Senior lecturer x1	0·5 days		
	Consultants x2	1 day		
	Nurse time travelling x 9	6·75 hours	0·78	0
	Round trips to training centre (nurses)	9 trips	0·10	0
	Refreshment	1 set	0·15	0
Training of Research assistants (All trial arms)	Research assistant x3	6·6 days	1·91	1·91
	Senior lecturer x1	0·5 days		
	Reader x1	0·5 days		
	Trial Manager x1	4 days		
Total cost per participant			104·64	44·83

^ Design was included as materials couldn't be used wholesale from a previous study and we judged that this may occur in the future following further learning from this trial*Value removed at present to maintain confidentiality
*Data source: Interviews with trial PI and trial manager, review of trial records, diaries, and routine administrative records

For peer review only

Table S2: Components of delivery cost of intervention (Post group)

Components	Resource (from administrative records)	Quantity of resource	Unit cost (data source)	3 months analysis		12 months analysis	
				Total cost	Cost per participant	Total cost	Cost per participant
Envelopes for posting pedometers (including replacement)	Number of envelopes	426	£0·03 (invoice)	£12·78	£0·04	£12·78	£0·04
Stamps for posting pedometer	Number of stamps	426	£2·50 (invoice)	£1,065	£3·14	£1,065	£3·14
Pedometers (including replacements) given to participants	Number of pedometers	426	£1 / £4*(invoice)	£426	£1·26	£1,704	£5·03
Replacement batteries for pedometer	Number of replacement batteries	11	£0·67 (invoice)	£7·37	£0·02	£7·37	£0·02
Patient handbooks	Number of handbooks	339	£0·80 (administrative records)	£271	£0·80	£271	£0·80
Step count diary	Number of diaries	339	£1·30 (administrative records)	£440·70	£1·30	£440·70	£1v30
Total cost per participant					£6·56		£10·33

*£1 was pro rata unit cost for 3 months and £4 is for 12 months. As pedometers were required only for the period of analysis but could be used beyond, their costs were spread over their expected lifetime, following Sharples et al (2014)[1]. As pedometers had an expected lifetime of 2 years, the average cost of pedometer was multiplied by 13[1]/104[2](weeks), in the case of 3 month analysis and 52/104 for the 12 month analysis.

[1] Intervention period in weeks
[2] Life expectancy of pedometer (in weeks)- based experience from PACE lift trial

For peer review only - http://bmjopen.bmj.com/site/about/guidelines.xhtml

Table S3: Components of delivery cost of intervention (Nurse group)

Components	Resource (data source)	Quantity of resource	Unit cost (data source)	3 months analysis		12 months analysis	
				Total cost	Cost per participant	Total cost	Cost per participant
Pedometers given to participants	Number of pedometers (administrative records)	346	£1 / £4* (Invoice)	£346	£1	£1384	£4
Patient handbooks	Number of handbooks (administrative records)	346	£0·80 (administrative records)	£277	£1	£277	£1
Step count diary	Number of diaries (administrative records)	346	£1·30 (administrative records)	£449·80	£1·30	£449·80	£1·30
RAs time to arrange consultation	Time spent by RAs (diary)	50·46 hours	£16·51 (administrative records)	£833·07	£2·41	£833·07	£2·41
Phone calls by RA to arrange consultation	Duration of phone calls (administrative records)	3,027·5 mins	£0·11 (BT tariff)	£333·03	£0·96	£333·03	£0·96
Cost of nurse visit per participant (project database for nurse group)					£43		£42
Total cost per participant					£49·67		£51·67

*£1 was pro rata unit cost for 3 months and £4 is for 12 months.

For peer review only

For peer review only - http://bmjopen.bmj.com/site/about/guidelines.xhtml

Table S4: Costs to participants of participating in interventions and physical activity

Participant costs	Control (n=323)	Post (n=312)	Nurse (n=321)
	£ Mean (SD)		
Intervention related			
Time working out how to use pedometer	0(0)	2 (6)	1 (3)
Time planning how to increase walking/step count	0(0)	5 (15)	3 4)
Time filling in PACE-UP diary	0(0)	51 (80)	58 (122)
Parking fees to visit nurse	0(0)	0(0)	0·11 (0.73)
Time spent in consultation with nurse	0(0)	0(0)	10 (5)
Time travelling (irrespective of mode of transport) to visit nurse	0(0)	0(0)	11 (10)
Transportation cost (for those who took public transport) of attending the nurse visit	0(0)	0(0)	0·13 (1.33)
Time waiting time prior to consultation with nurse	0(0)	0(0)	3 (4)
Child care during nurse visits	0(0)	0(0)	0·3 (3.21)
Personal costs of participation in physical activity	411 (817)	492 (1,293)	333 (684)
Personal costs from falls/ fractures/ sprains/ injuries	17 (103)	22 (184)	6 (40)

For peer review only

For peer review only - http://bmjopen.bmj.com/site/about/guidelines.xhtml

Table S5: Health service use by trial arm with unit costs

Health service use	Trial arm (Qty)			Unit cost (£) Weighted average (Q1 – Q3)	Source of unit cost
	Control n=323	Post n=312	Nurse n=321		
**Outpatient referrals (total)[2]**	**164**	**158**	**186**		
Opthalmology	10	18	15	86 (70-99)	DH (2015) National Reference Costs
Urology	4	3	6	99 (76-116)	
General medicine	4	0	2	157 (120-187)	
ENT	9	6	12	92 (70-109)	
Podiatry	9	7	7	44 (27-45)	
Trauma & orthopaedics	14	13	10	113 (88-133)	
Physiotherapy	26	33	37	46 (35-50)	
Nephrology	0	1	0	145 (94-178)	
Oral surgery	0	2	0	115 (85-142)	
Gynaecology	6	7	14	134 (104-164)	
Audiology	4	6	7	104 (55-174)	
Colorectal surgery	1	5	1	117 (83-135)	
Neurology	8	8	5	174 (136-204)	
Cardiology	12	5	4	131 (92-154)	
Gastroenterology	6	2	6	130 (99-153)	
Rheumatology	4	6	7	135 (99-150)	
Dermatology	1	8	7	98 (74-109)	
General surgery	4	1	3	125 (98-165)	
Endocrinology	2	1	2	144 (100-167)	
Neurosurgery	2	0	0	181 (138-228)	
Oncology	8	5	11	133 (97-165)	
Psychotherapy	1	0	0	100 (47-217)	
Respiratory medicine	4	6	3	150 (107-181)	
Clinical neurophysiology	2	0	1	165 (107-197)	
Programmed pulmonary rehab	0	0	1	20 (12-31)	
Pain management	2	0	4	135 (82-164)	
Allergy service	0	1	0	149 (126-175)	
Dietetics	2	2	3	62 (38-76)	
Vascular surgery	2	1	4	149 (100-176)	
Mental illness	1	1	1	234 (181-256)	
Clinical Genetics	1	0	1	429 (248-601)	
Clinical Haematology	2	1	0	160 (93-189)	
Spinal surgery services	0	1	0	142 (112-164)	
Maxillo-facial surgery	0	0	1	111 (70-133)	
Plastic surgery	1	1	1	93 (68-109)	
Clinical immunology	0	1	0	215 (140-243)	
Interventional radiology	1	0	0	192 (88-260)	
Breast surgery	9	4	5	139 (103-166)	
Tropical medicine	0	1	0	202 (203-203)	
Clinical psychology	1	0	3	177 (116-245)	
Old age psychiatry	0	1	2	108 (108-108)	
Referral to Accident & Emergency	1	0	0	135 (54-166)	
**Community based referrals (total)[3]**	**27**	**19**	**21**		
District nurse	1	3	2	39 (31-43)	PSSRU
Community Podiatrist	4	3	8	42 (35-58)	PSSRU
Community Dietitian	0	2	0	80 (53-96)	DH (2015) National Reference Costs
Smoking cessation (Nurse)	5	3	4	14	15.5 mins nurse time (Curtis 2014)
Healthy lifestyle (Nurse)	0	2	0	14	15.5 mins nurse time (Curtis 2014)
Community Gynaecologist	5	1	0	134 (104-164)	DH (2014) National Reference Costs
Community Physiotherapist	7	4	1	52 (44-58)	(Curtis 2014)
Community Diabetic	1	0	0	69 (38-93)	DH (2015) National Reference Costs
DESMOND diabetes programme	4	0	6	230	Gillett et al (2010) (inflated to 2014)
Expert Patient Programme	0	1	0	302	Richardson et al (2008) (inflated to 2014)
**Primary Care – excludes practice**	**2074**	**1748**	**2094**		

For peer review only

For peer review only - http://bmjopen.bmj.com/site/about/guidelines.xhtml

Health service use	Trial arm (Qty)			Unit cost (£) Weighted average (Q1 – Q3)	Source of unit cost
	Control n=323	Post n=312	Nurse n=321		
**visits related to the delivery and participation in intervention (total)[1]**					
GP (11.7mins)	1743	1436	1729	42	(Curtis 2014)
GP nurse (15.5mins)	331	312	365	14	(Curtis 2014)
**A&E visit[4]**	49	36	46	124	DH (2015) National Reference Costs
**Non- Elective hospital admissions (total)[5,6]**	**12**	**4**	**20**		
Biliary acute pancreatitis	0	0	3	2037 (1247-2492)	DH (2015) National Reference Costs
Cardiac catheterisation for coronary artery disease	1	0	1	2643 (1980-3028)	
Chest pain	0	1	0	490 (370-563)	
Abdominal pain	0	0	1	718 (922 -1298)	
Acute ST segment elevation myocardial infarction	2	0	0	1497 (1102-1740)	
Transient ischaemic attack	0	0	1	878 (643-994)	
Guillain-Barre syndrome	0	0	1	1571 (1069-1792)	
Pneumonia	1	0	0	1894 (1406-2238)	
Epilepsy	1	0	0	1125 (788-1266)	
Stroke and cerebrovascular accident	1	0	0	2817 (2018-3396)	
UTI	0	0	1	1530 (1187-1755)	
Detached Retina	0	0	1	908 (303-1935)	
Anxiety states	0	0	1	1393 (984-1628)	
Infective endocarditis in diseases EC, NOS	1	0	0	4480 (2351-5906)	
Acute appendicitis	0	0	1	3017 (2459-3365)	
IUD removed	0	0	1	1780 (1142-2135)	
Ankle fracture	1	0	0	3762 (3109-4271)	
no procedure (NES)	4	3	8	611 (408-726)	
**Elective hospital admissions (total)[5,7]**	**10**	**2**	**3**		
Cardiac catheterisation	2	0	0	2086 (1185-2709)	DH (2015) National Reference Costs
Percut tranlum balloon angioplasty mult coronary	1	0	0	1813 (880-2233)	
Inguinal hernia	0	1	0	2121 (1682-2392)	
Coronary artery bypass graft operations	0	1	0	9310 (7369-9929)	
Laparoscopic cholecystectomy	3	0	0	2567 (2082-2924)	
Endarterectomy of femoral artery NEC	0	0	2	6028 (4593-7209)	
Malignant neoplasm of female breast for chemotherapy	1	0	0	1780 (856-2139)	
Endarterectomy of carotid artery NEC	1	0	0	3911 (2986-4497)	
Neurophysiological operation NOS	2	0	0	1497 (1111-2118)	
Ovarian Cancer	0	0	1	1469 (741-1966)	
Total resource use (All HSU)	2336	1967	2370		

Unit costs are rounded to the nearest whole number and presented in the 2013/14 price year. The health service use presented in this table refers to the base case sample. All the data are based on participant-specific GP records for the trial period with different assumptions and approaches for costing by type of service use:

[1] *Primary care*: GP visits 11.7 minutes; Nurse visits 15·5 minutes;

[2] *Outpatient referrals*: where appropriate, linked to outpatient service descriptions in the reference costs (and reviewed by principal investigator) and a weighted (by throughout) average for consultant/non-consultant led attendances taken; referrals to private sector excluded (n=1);

[3] community referral services costed as referenced; if service use was unclear, an NHS hospital out-patient department was assigned by the principal investigator;

[4] *A&E visit*: as reason for A&E visits was not recorded, an average A&E visit cost for 2013-14 was assigned.

For peer review only - http://bmjopen.bmj.com/site/about/guidelines.xhtml

[5]*Hospital admissions*: The principal investigator (blind to study group) reviewed all hospital admissions, and provided either a 'best guess diagnosis/procedure' or listed 'unknown' (n=2). As details on the type of procedure or severity of the symptoms were not available, a weighted (by activity) average of all of the possible scores/procedures was used to derive average cost for elective.

[6]The unit cost for the emergency admissions are a weighted average of the non-elective short stay and non-elective long stay admissions, as the length of stay was unclear.

[7]Hospital admissions without a procedure were treated as non-elective short stay admissions (one day or less). Where hospital admission code was unclear the diagnosis was reviewed by the PI for advice on the nearest appropriate code.

For peer review only

For peer review only - http://bmjopen.bmj.com/site/about/guidelines.xhtml

Figure S1: Illustration of pathways within the long-term cost-effectiveness model (Anokye et al 2014a)

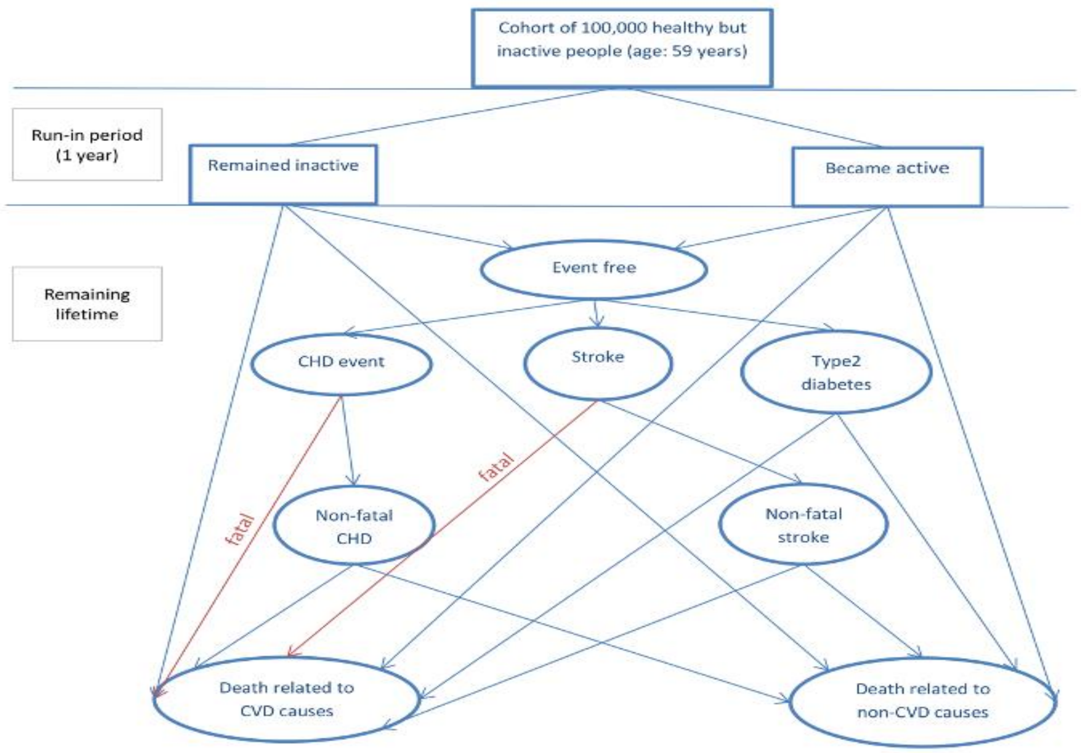

iew only

Table S6: Parameter values for long-term cost-effectiveness model

Parameter		Value	Source of data
Relative risks of:			
Becoming active (at year 1)*:	Postal vs control	1·8 (95% CI: 1·4, 2.3)	PACE-UP trial data
	Nurse vs control	1·7 (95% CI: 1·3, 2.2)	
	Nurse vs postal	0·9 (95% CI: 0·7, 1.3)	
Disease (active vs inactive)	CHD	0·90	Hu et al (2007)
	Stroke	0·86	Hu et al (2005)
	Diabetes	0·67	Hu et al (2003)
Non-CVD mortality after:	Non-fatal CHD	1·71	Bronum-Hansen et al (2001)
	Non-fatal Stroke	1·71	
	Diabetes	1·49	Preis et al (2009)
CVD mortality after:	Non-fatal CHD	3·89	Bronum-Hansen et al (2001)
	Non-fatal Stroke	3·89	
	Diabetes	2·61	Preis et al (2009)
CHD fatalities	59-64	11·55%	Ward et al (2005)
	65-74	21·07%	
	75+	14·76%	
Stroke fatalities	55-64	23·28%	Ward et al (2005)
	65-74	23·47%	
	75+	23·42%	
CHD incidence	59-64	0·63%	Ward et al (2005); NCGC (2011)
	65-74	0·97%	
	75+	0·97%	
Stroke incidence	59-64	0·29%	
	65-74	0·69%	
	75+	1·43%	
Diabetes incidence	59	0·06%	Gonzalez et al (2009)
	60-69	0·10%	
	70-79	0·11%	
	80+	0·11%	
Age-specific quality of life	59-64	0·82	Health Survey for England (2011)
	65-74	0·78	
	75+	0·72	
Health state utility weight	Healthy	1·00	Ward et al (2005); NCGC (2011)
	CHD 1st event	0·80	
	post CHD 1st event	0·92	
	Stroke 1st event	0·63	
	post stroke 1st event	0·65	
	Diabetes	0·90	
	Short term psychological benefit of achieving 150 mins of MVPA per week	0·01	PACE UP trial data
Annual costs	Control	£467 (95% CI 365 to569)	PACE UP trial data
	Postal	£376 (95% CI 307 to445)	

For peer review only - http://bmjopen.bmj.com/site/about/guidelines.xhtml

Parameter		Value	Source of data
	Nurse	£593 (95% CI 473 to714)	
	CHD 1st event	£4,248	NCGC (2011)
	post CHD 1st event	£485	
	Stroke 1st event	£10,968	
	post stroke 1st event	£2,409	
	Diabetes	£979	

*Relative risks (RR) for achieving at least 150 minutes of MVPA in ≥10 minute bouts at 12 months were estimated from odds ratios (OR) using the formula OR / {(1-P_{ref}) + (P_{ref} *OR)} where P_{ref} is the proportion of all subjects achieving 150 minutes of MVPA in ≥10 minute bouts at baseline i.e. 218/1023 = 0.21. The odds ratios had been derived from a logistic regression model in which the dependent variable, achieving 150 minutes of MVPA in bouts of ≥10 minutes at 12 months, was regressed on baseline minutes of MVPA in bouts of ≥10 minutes, month of baseline accelerometry, day order of wear, day of week, age, gender, general practice and treatment group, with household as a cluster.

For peer review only

For peer review only - http://bmjopen.bmj.com/site/about/guidelines.xhtml

Figure S2: Cost-effectiveness plane for postal vs control at 12 months

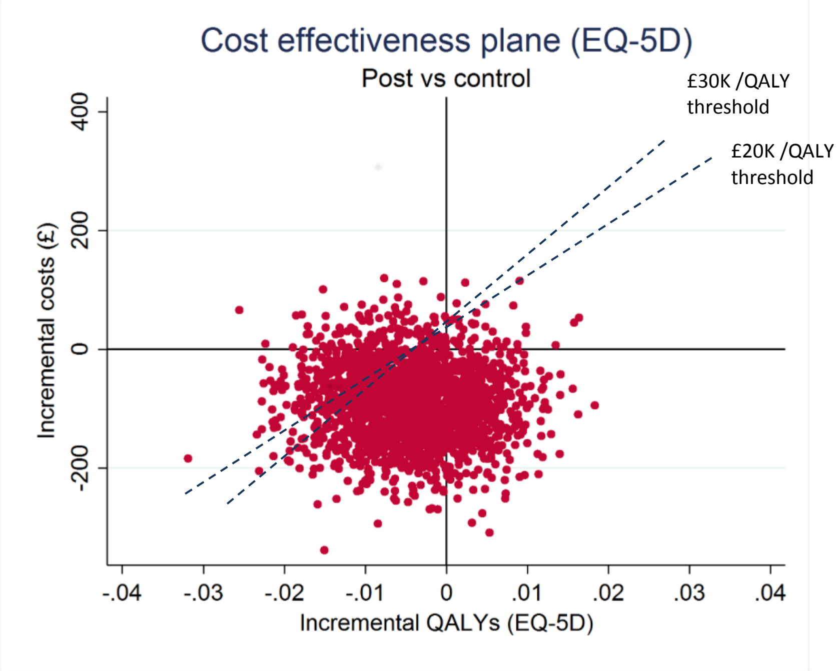

Figure S3: Cost-effectiveness plane for nurse vs control at 12 months

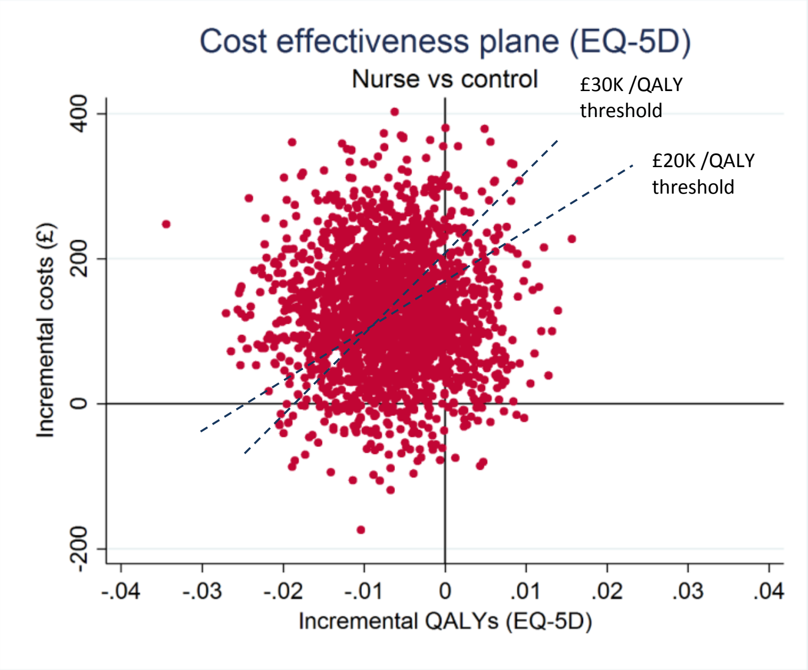

For peer review only - http://bmjopen.bmj.com/site/about/guidelines.xhtml

Figure S4: Cost-effectiveness plane for nurse vs post at 12 months

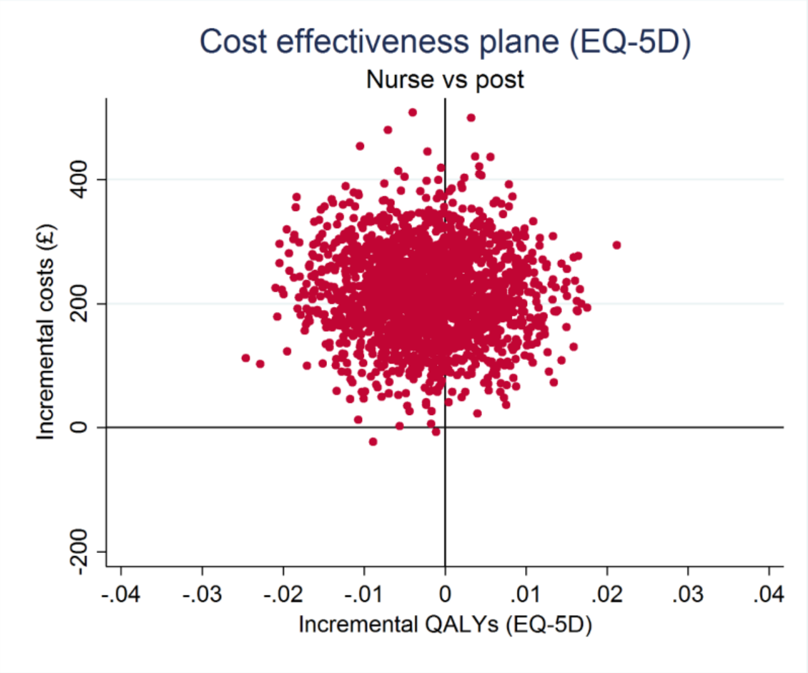

For peer review only - http://bmjopen.bmj.com/site/about/guidelines.xhtml

Table S7: Within trial sensitivity analyses (at 12 months)

Parameter	Post vs Control			Nurse vs Control			Nurse vs Post		
	Incremental cost (£)	Incremental QALY	ICER	Incremental cost(£)	Incremental QALY	ICER	Incremental cost (£)	Incremental QALY	ICER
	Mean (95% CI)			Mean (95% CI)			Mean (95% CI)		
Base case	-91* (-215, 3)	-0.0043 (-0.0172, 0.0087)	Less costly but less effective than control	126 (-37, 290)	-0.0066 (-0.0201, 0.0068)	Intervention dominated by control	217 (8, 354)	-0.0024 (-0.0156, 0.0109)	Nurse dominated by Post
Whole sample (all randomised)	-40 (-169, -89)	-0.0070 (-0.0195, 0.0054)	Less costly but less effective than control	150 (-6, 306)	-0.0093 (-0.0222, 0.0036)	Intervention dominated by control	190 (48, 332)	-0.0023 (-0.0148, 0.0102)	Nurse dominated by Post
Health service use including only GP data on referrals and admissions	-55 (-166, -56)	-0.0043 (-0.0172, 0.0087)	Less costly but less effective than control	129 (-17, 275)	-0.0066 (-0.020, 0.0068)	Intervention dominated by control	184 (61, 307)	-0.0024 (-0.0156, 0.0109)	Nurse dominated by Post
Health service use including only self-reported serious adverse effects	21 (-65, 107)	-0.0043 (-0.0172, 0.0087)	Intervention dominated by control	144 (65, 224)	-0.0066 (-0.020, 0.0068)	Intervention dominated by control	123 (47, 200)	-0.0024 (-0.0156, 0.0109)	Nurse dominated by Post
Health service use including only GP data on adverse effects	-11 (-107, 85)	-0.0043 (-0.0172, 0.0087)	Less costly but less effective than control	64 (-15, 142)	-0.0066 (-0.020, 0.0068)	Intervention dominated by control	74 (13, 135)	-0.0024 (-0.0156, 0.0109)	Nurse dominated by Post
Excluding all health service use cost	55.2 (55, 55.4)	-0.0043 (-0.0172, 0.0087)	Intervention dominated by control	156.2 (-154, 158)	-0.0066 (-0.0201, 0.0068)	Intervention dominated by control	101 (99, 103)	-0.0024 (-0.0156, 0.0109)	Nurse dominated by Post
Exclusion of missing data**	-91 (-215, 33)	-0.0088 (-0.0231, 0.0055)	Less costly but less effective than control	126 (-37, 290)	-0.0078 (-0.0233, 0.0076)	Intervention dominated by control	217 (8, 354)	0.0009 (-0.0141, 0.0160)	More costly but less effective than control (ICER:£241k)
Changing cost perspective (both participants (all participant costs) and NHS costs)	36 (-177, 250)	-0.0043 (-0.0172, 0.0087)	Intervention dominated by control	107 (-97, 311)	-0.0066 (-0.020, 0.0068)	Intervention dominated by control	71 (-150, 291)	-0.0024 (-0.0156, 0.0109)	Nurse dominated by Post
Changing cost perspective (both participants (part)[3] and NHS costs)	-22 (-235, 191)	-0.0043 (-0.0172, 0.0087)	Less costly but less effective than control	47 (-157, 250)	-0.0066 (-0.020, 0.0068)	Intervention dominated by control	69 (-152, 289)	-0.0024 (-0.0156, 0.0109)	Nurse dominated by Post
Combination of excluding all health service use cost and including all participants costs (minus health service use cost borne by participants)	179 (-1, 361)	-0.0043 (-0.0172, 0.0087)	Intervention dominated by control	153 (24, 281)	-0.0066 (-0.020, 0.0068)	Intervention dominated by control	-27 (-203, 149)	-0.0024 (-0.0156, 0.0109)	Less costly but less effective than control
Pedometer lasts for 1 year (equivalent to pedometers not being re-usable and full	-86 (-210, 38)	-0.0043 (-0.0172,	Less costly but less effective than control	130 (-33, 294)	-0.0066 (-0.0201,	Intervention dominated by	216 (80, 353)	-0.0024 (-0.0156,	Nurse dominated by

* This excludes time costs of working out how to use pedometer, diary, and planning to increase work

** The pattern of missing data for the base case analysis was multivariate (i.e. some but not all variables had data missing for some participants). The amount of missing data, where observed, was less than 5% except for EQ5D scores (baseline data: 5% (n=51); 3 months data: 7% (n=67); 12 months data: 8%(n=74)).

For peer review only - http://bmjopen.bmj.com/site/about/guidelines.xhtml

Parameter	Post vs Control			Nurse vs Control			Nurse vs Post		
	**Incremental cost (£)**	**Incremental QALY**	**ICER**	**Incremental cost(£)**	**Incremental QALY**	**ICER**	**Incremental cost (£)**	**Incremental QALY**	**ICER**
	**Mean (95% CI)**			**Mean (95% CI)**			**Mean (95% CI)**		
Base case	-91* (-215, 3)	-0.0043 (-0.0172, 0.0087)	Less costly but less effective than control	126 (-37, 290)	-0.0066 (-0.0201, 0.0068)	Intervention dominated by control	217 (8, 354)	-0.0024 (-0.0156, 0.0109)	Nurse dominated by Post
cost of pedometer borne in year 1)		0.0087)			0.0068)	control		0.0109)	Post
Pedometer lasts for 4 years (double length of life considered in base case)	-93 (-218, 31)	-0.0043 (-0.0172, 0.0087)	Less costly but less effective than control	124 (-39, 287)	-0.0066 (-0.0201, 0.0068)	Intervention dominated by control	218 (81, 354)	-0.0024 (-0.0156, 0.0109)	Nurse dominated by Post

For peer review only

References

Anokye NK., Lord J., Fox-Rushby J. Is brief advice in primary care a cost-effective way to promote physical activity?, *British Journal of Sports Medicine* 2014; **48 (3)** : 202–206

Brønnum-Hansen H, Davidsen M, Thorvaldsen P. Long-term survival and causes of death after stroke. *Stroke* 2001; **32**: 2131–6.

Curtis LA: Unit costs for health and social care Personal Social Services Research Unit, University of Kent: Canterbury; 2014.

De Cocker K, De Bourdeaudhuij I, Brown W, Cardon G. Moderators and mediators of pedometer use and step count increase in the "10,000 Steps Ghent" intervention. *International Journal of Behavioral Nutrition and Physical Activity* 2009; **6**:3 http://dx.doi.org/10.1186/1479-5868-6-3

Department of Health (2015). NHS Reference Costs 2013 to 2014. Available from:https://www.gov.uk/government/publications/nhs-reference-costs-2013-to-2014

Gillett M, Dallosso HM, Dixon S, et al. Delivering the diabetes education and self- management for ongoing and newly diagnosed (DESMOND) programme for people with newly diagnosed type 2 diabetes: cost effectiveness analysis. *BMJ 2010*: 341:c4093

Gonzalez ELM, Johansson S, Wallander MA. Trends in the prevalence and incidence of diabetes in the UK, 1996–2005. *J Epidemiol Community Health* 2009; **63**:332–6.

Hu G, Jousilahti P, Borodulin K, et al. Occupational, commuting and leisure-time physical activity in relation to coronary heart disease among middle-aged Finnish men and women. *Atherosclerosis* 2007; **194**:490–7

Hu G, Sarti C, Jousilahti P, et al. Leisure time, occupational, and commuting physical activity and the risk of stroke. *Stroke* 2005; **36**:1994–9.

Hu G, Qiao Q, Silventoinen K, et al. Occupational, commuting, and leisure-time physical activity in relation to risk for type 2 diabetes in middle-aged Finnish men and women. *Diabetologia* 2003; **46**:322–29

Health Survey for England, 2011 National Centre for Social Research, University College London-Department of Epidemiology and Public Health. [computer file]. *3rd Edition.* Colchester, Essex: UK Data Archive [distributor], July 2011. SN: 6397

National Clinical Guideline Centre (NCGC). Hypertension: the clinical management of primary hypertension in adults. Clinical guideline: methods, evidence and recommendations. London: National Institute for Health and Clinical Excellence. 2011. Ref Type: Report.

Preis SR, Hwang SJ, Coady S, et al. Trends in all-cause and cardiovascular disease mortality among women and men with and without diabetes mellitus in the Framingham Heart Study, 1950 to 2005. *Circulation* 2009; **119**:1728–35.

Richardson G, Kennedy A, Reeves D, et al. A Cost effectiveness of the Expert Participants Programme (EPP) for participants with chronic conditions. *Journal of Epidemiology and Community Health* 2008; **62**: 361–367

For peer review only - http://bmjopen.bmj.com/site/about/guidelines.xhtml

Sharples L, Glover M, Bennett M, Jordan J, Clutterbuck-James A, Davies M, Smith I, Cameron M, Chadwick R, Oscroft N, Morrell M, Fox-Rushby J, Pittman M, Quinnell T (2014) TOMADO: A Crossover Randomised Controlled Trial of Oral Mandibular Advancement Devices for Obstructive Sleep Apnoea-Hypopnoea *Health Technology Assessment 18, 67, http://www.journalslibrary.nihr.ac.uk/hta/volume-18/issue-67#*

Ward S, Jones ML, Pandor A, et al. Statins for the prevention of coronary events. London. National Institute for Clinical Excellence 2005; Ref Type: Report World Health Organisation (2010) Global recommendations on physical activity for health http://www.who.int/dietphysicalactivity/publications/9789241599979/en/

For peer review only

For peer review only - http://bmjopen.bmj.com/site/about/guidelines.xhtml

Harris et al. Trials 2013, 14:418
http://www.trialsjournal.com/content/14/1/418

TRIALS

STUDY PROTOCOL Open Access

PACE-UP (Pedometer and consultation evaluation - UP) – a pedometer-based walking intervention with and without practice nurse support in primary care patients aged 45–75 years: study protocol for a randomised controlled trial

Tess Harris[1], Sally M Kerry[2], Christina R Victor[3*], Sunil M Shah[1], Steve Iliffe[4], Michael Ussher[1], Ulf Ekelund[5,6], Julia Fox-Rushby[7], Peter Whincup[1], Lee David[8], Debbie Brewin[8], Judith Ibison[1], Stephen DeWilde[1], Elizabeth Limb[1], Nana Anokye[7], Cheryl Furness[1], Emma Howard[1], Rebecca Dale[1] and Derek G Cook[1]

Abstract

Background: Most adults do not achieve the 150 minutes weekly of at least moderate intensity activity recommended for health. Adults' most common physical activity (PA) is walking, light intensity if strolling, moderate if brisker. Pedometers can increase walking; however, most trials have been short-term, have combined pedometer and support effects, and have not reported PA intensity. This trial will investigate whether pedometers, with or without nurse support, can help less active 45–75 year olds to increase their PA over 12 months.

Methods/design: *Design:* Primary care-based 3-arm randomized controlled trial with 12-month follow-up and health economic and qualitative evaluations.

Participants: Less active 45–75 year olds (n = 993) will be recruited by post from six South West London general practices, maximum of two per household and households randomised into three groups. Step-count and time spent at different PA intensities will be assessed for 7 days at baseline, 3 and 12 months by accelerometer. Questionnaires and anthropometric assessments will be completed.

Intervention: The pedometer-alone group will be posted a pedometer (Yamax Digi-Walker SW-200), handbook and diary detailing a 12-week pedometer-based walking programme, using targets from their baseline assessment. The pedometer-plus-support group will additionally receive three practice nurse PA consultations. The handbook, diary and consultations include behaviour change techniques (e.g., self-monitoring, goal-setting, relapse prevention planning). The control group will receive usual care.

Outcomes: Changes in average daily step-count (primary outcome), time spent sedentary and in at least moderate intensity PA weekly at 12 months, measured by accelerometry. Other outcomes include change in body mass index, body fat, self-reported PA, quality of life, mood and adverse events. Cost-effectiveness will be assessed by the incremental cost of the intervention to the National Health Service and incremental cost per change in step-count
(Continued on next page)

* Correspondence: christina.victor@brunel.ac.uk
[3]Gerontology and Health Services Research Unit, Brunel University, London UB8 3PH, UK
Full list of author information is available at the end of the article

BioMed Central

© 2013 Harris et al.; licensee BioMed Central Ltd. This is an Open Access article distributed under the terms of the Creative Commons Attribution License (http://creativecommons.org/licenses/by/2.0), which permits unrestricted use, distribution, and reproduction in any medium, provided the original work is properly cited. The Creative Commons Public Domain Dedication waiver (http://creativecommons.org/publicdomain/zero/1.0/) applies to the data made available in this article, unless otherwise stated.

For peer review only - http://bmjopen.bmj.com/site/about/guidelines.xhtml

(Continued from previous page)
and per quality adjusted life year. Qualitative evaluations will explore reasons for trial non-participation and the interventions' acceptability.

Discussion: The PACE-UP trial will determine the effectiveness and cost-effectiveness of a pedometer-based walking intervention delivered by post or practice nurse to less active primary care patients aged 45–75 years old. Approaches to minimise bias and challenges anticipated in delivery will be discussed.

Trial registration: ISRCTN98538934

Keywords: Accelerometers, Behaviour change techniques, Cognitive behavioural, Middle-aged adults, Older people, Pedometers, Physical activity, Postal, Practice nurse, Primary care, Walking intervention

For peer review only

Background

Benefit and risks of PA and current PA guidelines

Why is physical activity (PA) important for adults and older adults? PA leads to reduced mortality, a reduced risk of over 20 diseases and conditions, and improved function, quality of life and emotional well-being [1]. Physical inactivity is the fourth leading risk factor for global mortality [2] and a major cost burden on health services [1].

What are the PA guidelines? Adults and older adults are advised to be active daily and, in order to obtain health benefits, should achieve at least 150 minutes (2 ½ hours) per week of at least moderate intensity activity in bouts of 10 minutes or more. One effective way to do this is by 30 minutes of moderate intensity activity on at least 5 days weekly [1,3,4]. Regular walking is the most common PA of adults and older adults, walking at a moderate pace (3 mph /5 km/h) qualifies as moderate intensity PA [5]. Time spent being sedentary for extended periods should also be minimised, as this is an independent disease risk factor [1] and increases steeply from the age of 45 [6]. Whilst amongst adults in England aged 16 and over, 39% of men and 29% of women were judged to meet the recommended PA levels, based on their self-reported data, only 20% and 17% of men and women aged 60–74 met recommended levels [6], despite most of these inactive older people being capable of walking [7]. Lower socioeconomic groups [6] and Indian, Pakistani, Bangladeshi and Chinese ethnic groups are significantly less likely to report activity levels that meet the recommended levels, whilst the activity levels of other ethnic groups (Black Caribbean, Black African and Irish) are similar to that of the general population [8]. Surveys of adults in Europe and the USA also confirm that over 50% do not achieve public health PA recommendations [9,10]. Since PA, including walking, is unreliably recalled, surveys may overestimate PA levels [11]. Objective accelerometer measurement found that only 5% of men and 4% of women aged 35–64 years and 5% men and 0% of women aged 65 or more achieved the recommended PA levels, only a fraction of those self-reporting achieving these levels [6].

What are the risks from increasing PA? Risks from a sedentary lifestyle far exceed the risks from regular PA [3,12,13]. Moderate intensity PA carries a low injury risk [14], mainly musculoskeletal injury or falls [15]. Walking is very low risk, "a near perfect exercise" [5]. Screening participants for contraindications before participating in light to moderate intensity PA programmes is no longer advocated [3,16]. An important safety feature of our study is that individualised goals can be set from the participant's own baseline, in line with advice that older adults in particular should start with low intensity PA and increase intensity gradually, the "start-low-and-go-slow" approach [12,13].

Strategies for increasing PA

How can adults and older adults increase their PA levels? A systematic review of PA interventions reported moderate positive short-term effects, but findings were limited by mainly unreliable self-report measures in motivated volunteers [17]. Effective interventions explored factors associated with behavioural change, including beliefs about costs and benefits of PA [18]. Exercise programs in diverse populations can promote short- to medium-term increases in PA when interventions are based on health behaviour theoretical constructs, individually tailored with personalised activity goals and use behavioural strategies [3,19]. A critical review and a best practices statement on older peoples' PA interventions advised home rather than gym-based programmes and behavioural strategies (e.g., goal-setting, self-monitoring, self-efficacy, support, relapse prevention training) rather than health education alone [13,20]. National Institute for Health and Clinical Excellence (NICE) guidance concluded that no particular behaviour change model was superior and that training should focus on generic competencies and skills rather than specific models [21]. Starting low, but gradually increasing to moderate intensity is promoted as best practice, with advice to incorporate interventions into

For peer review only - http://bmjopen.bmj.com/site/about/guidelines.xhtml

Harris et al. Trials 2013, 14:418
http://www.trialsjournal.com/content/14/1/418

the daily routine (e.g., walking) [13]. A recent systematic review concluded that walking interventions tailored to people's needs, targeted at the most sedentary and delivered at the individual or household level, can be effective, although evidence directly comparing interventions targeted at individuals, couples or households is lacking [22].

Are pedometers helpful? Pedometers are small, inexpensive devices, worn at the hip, that provide direct step-count feedback. A systematic review of 26 studies found pedometer users increased steps/day by 2,491 (1,098–3,885) and PA levels by 27%, with significant reductions in body mass index (BMI) and blood pressure [23]. A second review (32 studies) found an average increase of 2,000 steps/day for pedometer users [24]. Step-goals and diaries were key motivational factors [23,24]. Several limitations were recognised. Study sizes were relatively small and long-term effects undetermined; many included several components (e.g., pedometer and support) so independent effects were difficult to establish and the inclusion of older people and men was very limited [23,24]. Recent studies have addressed some of these limitations. A trial of 210 older women found that a pedometer plus behaviour change intervention increased PA at 3 months but not at 6 months [25]. Two trials in high risk groups (cardiac disease and impaired glucose tolerance) showed sustained increases in step-count at 12 months [26,27]. NICE recently updated its advice from only advising pedometers as part of research [28] to now advising their use as part of packages including support to set realistic goals, monitoring and feedback [29].

How do step-count goals relate to PA recommendations? Step-count goals lead to more effective interventions, but no specific approach to goal-setting is favoured [23]. Goals are based on either a fixed target (e.g., 10,000 steps/day) [30,31] or by advising incremental increases on baseline, as a percentage (5% per week [32], 10% biweekly [33] or 20% monthly [25]) or by a fixed number of extra steps. Those advocating a fixed number of extra daily steps have developed step-based guidelines to fit with existing evidence based guidelines with their emphasis on 30 minutes of at least moderate intensity PA on 5 or more days weekly [34]. Despite individual variation, moderate intensity walking appears approximately equal to at least 100 steps per minute [34,35]. Multiplied by 30 minutes this produces a minimum of 3,000 steps per day, to be done over and above habitual activity. Several studies have advocated adding in 3,000 steps/day on most days weekly, either from the beginning [26] or by increasing incrementally (initially an extra 1,500 steps/day and increasing) [36,37] or increasing by 500 steps/day biweekly [27]. Studies that advised adding 3,000 steps/day to baseline produced significant improvements in step-counts at 3 months and two measured outcomes at 12 months and showed sustained improvements in step-counts [26,27], waist circumference [26] and fasting glucose levels [27]. Although there is no evidence at present to inform a moderate intensity cadence (steps/minute) in older adults, Tudor-Locke et al. advocate using the adult cadence of 100 steps/minute in older adults (whilst recognising that this may be unobtainable for some individuals) and advise that the 30 minutes can be broken down into bouts of at least 10 minutes [38]. This model was used in a primary care walking intervention in 41 older people which found significant step-count increases from baseline to week 12, maintained at week 24 [39,40].

Could accelerometers be useful in a pedometer-based walking intervention? Accelerometers are small activity monitors, worn like pedometers, more expensive, but able to provide a time-stamped record of PA frequency (step-counts) and intensity (activity counts). They require computer analysis and give no immediate feedback, functioning as blinded pedometers in objectively measuring baseline and outcome data, but providing objective data on time spent in different PA intensities, including time spent in at least moderate intensity activity and time spent sedentary, two important public health outcomes. Pedometer studies without accelerometers have relied on self-report measures of these outcomes. Accelerometers are valid and acceptable to adults [6,41] and older adults [6,42,43]. Although both instruments measure step-count and are highly correlated [44,45], pedometers usually record lower step-counts, particularly at lower walking speeds, and accelerometers cannot reliably be substituted for pedometers at an individual level [45]. Thus, although we will use the accelerometer to measure outcomes, we will use a blinded pedometer, worn simultaneously at baseline, to set individual step-count targets.

Are pedometers cost-effective? There is limited knowledge on the cost-effectiveness of pedometer-based interventions in the UK. Recent systematic reviews that considered the economic outcomes of pedometer-based interventions found no evidence [46,47], partly attributable to insufficient data [48]. However, a recent study assessed the cost-effectiveness of giving an individualised walking programme and pedometer with or without a consultation compared with usual walking activity alongside a trial of 79 people [49]. The incremental cost-effectiveness ratios per person achieving an additional 15,000 steps/week were £591 and £92 with and without the consultation. However, no data on quality of life were collected and impacts on long-term outcomes were not estimated.

What is primary care's role in promoting PA? Primary care centres (general practices) in the UK provide healthcare and health promotion free at the point of access, to a

For peer review only

Harris et al. Trials 2013, 14:418
http://www.trialsjournal.com/content/14/1/418

registered list of local patients, using disease registers to provide annual or more frequent review of chronic disorders (for many of which PA will be of benefit), via a multidisciplinary health care team to provide continuity of care. NICE guidance found that brief interventions in primary care are cost-effective and therefore recommends that all primary care practitioners should take the opportunity, whenever possible, to identify inactive adults and provide advice on increasing PA levels [28]. New National Health Service health checks include adults up to age 74 and incorporate advice on increasing PA, often by primary care nurses [50]. Primary care nurses have been shown to be effective at increasing PA, particularly walking, in this age group [51]. Health professional PA advice in consultations is individually tailored [52] and has greater impact than other PA advice [53]. PA promotion by other routes, for older adults in particular, is unlikely to be as effective [54]]. Exercise prescribing guidance in primary care reinforces the importance of follow-up to chart progress, set goals, solve problems, and identify and use social support [55]; this will be an important feature of the nurse PA consultations in this trial. Evaluation of the UK Step-O-Meter Programme, delivering pedometers through primary care, showed self-reported PA increases, but advised investigation with a RCT design [36]. Two small trials have assessed the effectiveness of pedometers plus PA consultations: one showed a significant effect on step-counts at 12 weeks in 79 middle-aged adults [37]; the other showed a significant effect on step-counts at 12 weeks, maintained at 24 weeks in 41 older primary care patients and called for a further, larger primary care trial [39,40].

Theory on which the intervention is based and relevant pilot and preparatory work. The pedometer-based intervention is centred on work cited above showing that pedometers can increase step-counts and PA intensity [23,24], but extending this to ensure that the study covers older adults, men, has a 12 month follow-up, and is designed to examine pedometer and support components separately. The patient handbook, diary and practice nurse PA consultations will use behaviour change techniques (BCTs) (e.g., goal-setting, self-monitoring, feedback, boosting motivation, encouraging social support, addressing barriers, relapse anticipation etc.). These techniques have been successfully used by non-specialists in primary care after brief training [56] and are emphasized in the Health Trainer Handbook [57], based on evidence from a range of psychological methods and intended for National Health Service behaviour change programmes, with local adaptation [57]. We have adapted the Health Trainer Handbook for use in this trial into PACE-UP nurse and patient handbooks, to focus specifically on PA using pedometers. The BCTs have been classified according to Michie's refined taxonomy of BCTs for PA interventions [58] (Tables 1 and 2). Diary recording of pedometer step-counts provides clear material for PA goal setting, self-monitoring and feedback, and should fit well with this approach. Relevant pilot and preparatory work includes observational work using pedometers and accelerometers in primary care [42] and a trial with older primary care patients developing the PA consultations and pedometer-based walking intervention (PACE-Lift trial ISRCTN42122561) [59].

Study rationale and aims

Rationale

There is a need for a large, adequately powered primary care trial to test the effect of a pedometer-based walking intervention, with and without nurse PA consultations in inactive adults and older adults. It should include follow-up to 1 year and ensure that adequate numbers of men, older adults and individuals from diverse socioeconomic and ethnic backgrounds are included. It should enable the effectiveness of taking part as an individual or as a couple to be estimated. For greatest effect the intervention should use step-goals and diaries and the PA consultations and patient handbook should be based on BCTs, such as those used in the Health Trainer Handbook [57]. To objectively test the interventions' effectiveness on important public health outcomes, such as time spent in at least moderate intensity activity and time spent sedentary, accelerometer measurement of outcomes should be included. A qualitative assessment is needed to explore the intervention's acceptability and reasons for dropout and durability of effects. An economic evaluation should be performed alongside the trial and the costs and benefits of the alternatives, modelled beyond the end of the trial.

Study aims

The main hypotheses to be addressed are: i) does a 3 month pedometer-based walking intervention increase PA in inactive 45–75 year olds at 12 month follow-up; and ii) does providing practice nurse support through PA consultations provide additional benefit. The study will also assess the cost-effectiveness of both interventions and whether or not any effects are modified by age, gender, body mass index or taking part as a couple, and will estimate the effect of the interventions on patient reported outcomes and anthropometric measures.

Methods/design

This paper was written according to CONSORT reporting guidelines for RCTs of non-pharmacologic treatment [60].

Trial design

A three-arm parallel design cluster RCT with household as the unit of randomisation comparing the following:

For peer review only - http://bmjopen.bmj.com/site/about/guidelines.xhtml

Table 1 PACE-UP patient handbook and diary, and behavioural change techniques included

	Guide to content	Behavioural change techniques [58]
Patient handbook	Health benefits of increasing walking	1, 2
	PA guidelines	4
	Moderate intensity PA and relating it to number of steps	
	PACE-UP walking programme and step-count targets	7,9,16
	Review participant baseline step-count	19
	How to increase PA safely	21
	Useful websites	4
	How to keep going when PACE-UP programme finishes	1,2,16,26,29,35
Patient diary	How to use pedometer and record steps in diary	16, 21
	Frequently asked questions on PACE-UP trial	
	Weekly recording of step-count and walking in diary (weeks 1–12)	7,9,19,26
	Achievement of targets (weeks 1–12)	10,12,13
	Planning when to walk, where to walk, who to walk with	20,29
	Week 2 Tips and motivators: make walking part of your daily routine	20
	Week 3 Ttips and motivators: remember personal benefits, what to do if you	2,20,35
	are falling behind your targets	
	Week 4 Keep it up: praise and reward yourself, encouraging social support	12,13,29
	Week 5 Keep motivated: write down step-counts, ask for support	12,16,29
	Week 6 Now we are moving: obstacles and solutions	8
	Week 7 How to make these changes permanent – ideas for new walks, making time for walking, what gains have been made so far?	38,17,11
	Week 8 Maintain the gain: pacing, tips for safe exercising	9,21,35
	Week 9 Be busy being active: keep monitoring with pedometer, places, people and thoughts that motivate you	16,29,36
	Week 10 Change does not happen in a straight line! Preparing for setbacks	8,35
	Week 11 Make it a healthy habit: building regular exercise habits, creating if-then plans	1,2,7,23
	Week 12 I've changed: how to keep up your walking programme	16,20,29
	Congratulations you have completed the programme	11,16,17
	How to keep going when PACE-UP programme finishes	1,16,29

1. Provide general information on behaviour-health link; 2. Provide information on consequences to individual; 4. Provide normative information about others' behaviour; 7. Action planning; 8. Barrier identification; 9. Set graded tasks; 10. Prompt review of behavioural goals; 11. Prompt review of outcome goals; 12. Prompt rewards contingent on effort; 13. Prompt rewards contingent on successful behaviour; 16. Prompt self-monitoring of behaviour; 17. Prompting self-monitoring of behavioural outcome; 19. Provide feedback on performance; 20. Provide information on when and where to perform the behaviour; 21. Provide instructions on how to perform the behaviour; 23. Teach to use prompts/cues; 26. Prompt practice; 29. Plan social support/social change; 35. Relapse prevention/coping planning; 36. Stress management/emotional control training; 38. Time management.

a control group (usual PA); pedometer and written instructions by post; pedometer and support (written instructions and brief individually tailored PA consultations with a practice nurse). A 1:1:1 allocation will be used. The CONSORT flow diagram summarises the design, procedures and stages (Figure 1) [60].

Practice and participant recruitment

Practice inclusion criteria

South West London general practices with a list size >9,000; giving a commitment to participate over the study duration; having a practice nurse to carry out the PA consultations; and a room for the research assistant to recruit participants and conduct assessments.

Practice recruitment

The Primary Care Research Network Greater London will help us to identify potential participant practices within South West London who fit the above practice inclusion criteria. Approaches by mailed invitation, telephone contact with practice managers and personal contact with local general practitioners (GPs) and practice nurses will all be used as necessary to identify practices. We will select six from the list of potentially interested practices to include a range of socio-demographic factors

For peer review only - http://bmjopen.bmj.com/site/about/guidelines.xhtml

Table 2 PACE-UP practice nurse physical activity consultations and behaviour change techniques included

Week	Sessions	Guide to session content	Behavioural change techniques [58]
1	Session 1: First steps (30 minutes) Week 1	Review health status, current activity, health benefits of PA	1, 2
		Cost-benefit analysis for increasing PA	2
		PA guidelines and how to increase PA safely	4, 21
		Moderate intensity PA and relating it to number of steps	
		Review participant baseline step-count	19,
		Teach use of pedometer and recording walks and steps in diary	21, 26
		Ideas for increasing steps	20
		Goal-setting – PACE-UP goals or tailored to the individual patient	7, 9, 16
		Use of rewards for effort and for achieving goals	12, 13
		Summarise and check patient understanding, plan time for next meeting	
		Communication strategies to overcome resistance and promote patient-led change	37
5	Session 2: Continuing the changes (20 minutes) Week 5	Review step-count and walking diary	10, 19
		Encourage progress in increasing walking and achieving step-count goals	12, 13
		Troubleshoot any problems with pedometer or diary	8
		Review target and agree goals for next stage	7, 9, 16
		Barriers and facilitators to increasing PA, overcoming barriers, encouraging support	8, 29
		Pacing and avoiding boom and bust	9, 35
		Check confidence levels, build confidence to make change	18, 29, 36
		Summarise and check patient understanding, plan time for next meeting	
		Communication strategies to overcome resistance and promote patient-led change	37
9	Session 3: Building lasting habits (20 minutes) Week 9	Review step-count and walking diary	10, 19
		Review overall progress over the sessions	11, 17
		Encourage progress in increasing walking and achieving goals	12, 13
		Preparing for setbacks	35
		Building habits: discuss methods of maintaining lasting change, including repetition, if-then rules and support	7, 29, 23, 29, 35
		Setting goals: maintaining current activity or increasing further?	7, 9, 16, 26
		Remind re contact with research assistant in 3–4 weeks	
		Communication strategies to overcome resistance and promote patient-led change	37

1. Provide general information on behaviour-health link; 2. Provide information on consequences to individual; 4. Provide normative information about others' behaviour; 7. Action planning; 8. Barrier identification; 9. Set graded tasks; 10. Prompt review of behavioural goals; 11. Prompt review of outcome goals; 12. Prompt rewards contingent on effort; 13. Prompt rewards contingent on successful behaviour; 16. Prompt self-monitoring of behaviour; 17. Prompting self-monitoring of behavioural outcome; 18. Prompting focus on past success; 19. Provide feedback on performance; 20. Provide information on when and where to perform the behaviour; 21. Provide instructions on how to perform the behaviour; 23. Teach to use prompts/cues; 26. Prompt practice; 29. Plan social support/social change; 35. Relapse prevention/coping planning; 36.Stress management/emotional control training; 37. Motivational interviewing.

(including targeting some practices in areas with high numbers of ethnic minority patients) and geographical circumstances based on practice postcode index of multiple deprivation scores using national quintiles (at least 1 practice from each quintile). The index of multiple deprivation score includes factors such as distance to services, crime rates and road traffic accident rates, which could influence likelihood of outdoor PA, as well as material deprivation measures [61].

Participant inclusion criteria

Patients aged 45–75 years registered at a selected general practice, able to walk outside the home and with no contraindications to increasing their moderate intensity PA levels.

Participant exclusion criteria

In order to maximise the benefits of the intervention to individuals and the National Health Service, the trial

For peer review only

Harris *et al. Trials* 2013, **14**:418
http://www.trialsjournal.com/content/14/1/418

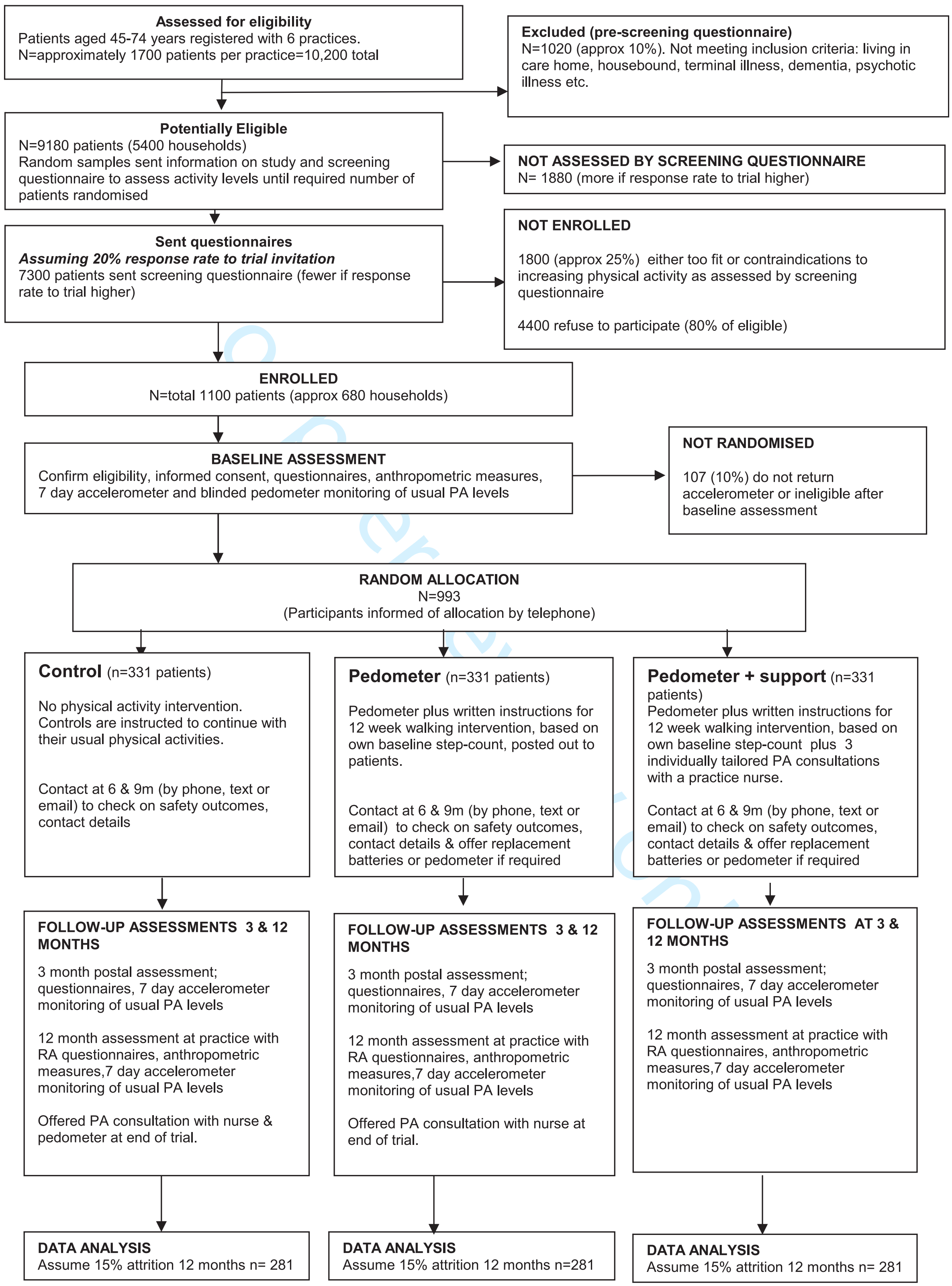

Figure 1 CONSORT flow diagram for PACE-UP trial. Detailed legend: CONSORT flow diagram showing participant flow through each stage of the randomized controlled trial (enrolment, intervention allocation, follow-up and data analysis).

focusses on less active adults, using a single-item validated questionnaire measure of self-reported PA as a screening question to identify them [51]. Those individuals reporting achieving a minimum of 150 minutes of at least moderate intensity PA weekly [1] will be excluded. Participants found on subsequent baseline accelerometer assessment to be above this PA level will not be excluded, as these patients would be included if this intervention were to be rolled out in primary care. Other exclusions: living in a residential or nursing home; housebound; ≥3 falls in previous year or ≥1 fall in previous year requiring medical attention; terminal illness; dementia or significant cognitive impairment (unable to follow simple instructions); registered blind; new onset chest pain, myocardial infarction, coronary artery bypass graft or angioplasty within the last 3 months; medical or psychiatric condition which the GP considers excludes the patient (e.g., acute systemic illness such as pneumonia, unstable heart failure, unable to move about independently, psychotic illness). Pregnant women will also be excluded.

Participant recruitment

The number of patients aged 45–74 years will be recorded at each practice. Practice staff will search practice electronic primary health care records to identify patients aged 45–74, using Read codes supplied by researchers and local care home knowledge to exclude ineligible patients (as above). Initial sampling will include 45–74 year olds, but some individuals will become 75 before randomisation and will still be included. A list of potentially eligible patients will be created and ordered by household, with each household given a unique household identifier. We are aiming to select either individuals or couples in a household, therefore we want to select a maximum of two people per household. If a household with one individual is selected at random, then that individual is selected. If a household has two or more individuals then one individual is selected at random. If there is a second individual in that household with an age difference of 15 years or less, they will also be selected. The approach was based on previous validated work showing that this age difference is an effective way of identifying (married or cohabiting) couples within a household [62]. Initially, the first random sample containing 400 eligible patients will be selected at each practice and the list examined by practice GPs or nurses to ensure trial suitability. Patients in these households will then be mailed an individual trial invitation letter from the practice and the screening question to assess activity levels and a participant information sheet. This will make it clear that if potential participants have any difficulties understanding, speaking or reading English they should bring a family member or friend with them to the research assistant appointment. The participant information sheet will be translated into different languages if practices indicate this to be appropriate. The 400 individuals will be contacted by post in a staggered manner over 2–3 months to avoid overwhelming the research assistants. Reminders will be sent out to non-responders after 6–8 weeks. Further random samples of households will be selected from the list until required numbers have been randomised. On the reply slip, those not wishing to participate will be asked about reasons for declining and their willingness to fill in a health and PA questionnaire, one of the questions on this questionnaire will ask if they would be willing to be interviewed about their reasons for not wanting to participate in the trial. Patients who agree to participate in the trial will be telephoned to arrange a baseline assessment at the practice with the research assistant. Two eligible people within a household will be invited together (or apart if they prefer). Eligibility will be confirmed and informed consent sought at this appointment.

Participant selection for the qualitative evaluation

Participant selection for the qualitative evaluation will run parallel to the trial and will focus upon three distinct groups. i) Trial 'non-participants' who agree to be interviewed, to explore factors influencing their decision not to participate. ii) Purposive samples of four groups of trial participants, after 12-month follow-up (including samples of those who did and did not increase their PA in each of the two intervention arms). The samples will reflect the range of socio-demographic characteristics of participants including ethnicity. iii) All practice nurses (maximum 12 if two per practice) will be invited to participate in a focus group to find out their thoughts about the interventions' acceptability and use in PA consultations. Interviewing with study participants will continue until no new themes are identified (approximately 55–80 are anticipated, 15–20 for the 'non-participants' and 10–15 for each of the four groups of trial participants).

Baseline assessment

The following assessments will be carried out by the research assistant at the patient's general practice.

i) *Questionnaire measures* – Socio-economic-demographic measures: marital status, ethnic group, occupation, employment, household composition, home ownership. Self-reported PA: modified Zutphen [63]. Health problems and lifestyle factors: self-reported chronic diseases (e.g., heart disease, lung disease, arthritis, depression), disability [64], medication, smoking and alcohol. Patient Reported Outcomes (PROs): exercise self-efficacy [65], anxiety and depression (Hospital Anxiety & Depression Scale [66]), perceived health status (EQ-5D) [67], loneliness [68]. A further self-report questionnaire of 7-day PA recall using the General Practice PA

For peer review only

Harris et al. Trials 2013, 14:418
http://www.trialsjournal.com/content/14/1/418

Questionnaire (GPPAQ) [69] and International Physical Activity Questionnaire (IPAQ) [70] will be completed after wearing the PA monitors for 7 days and posted back with them.

ii) *Falls Risk Assessment Tool* [71] – This will be assessed using self-report items and by direct observation of the ability to rise from a chair of knee height without using their arms.

iii) *Anthropometric measures* – Height (measured in bare feet to neared 0.5 cm using a stadiometer); weight (measured to nearest 0.1 kg), body fat, bioimpedance (using Tanita body composition analyser BC-418 MA); and waist and hip circumference (using standard technique and tape measure with clear plastic slider).

iv) *Objective PA assessment* – Measurement of usual PA levels, wearing an accelerometer and a blinded pedometer (Yamax Digiwalker CW200) on a belt over one hip, all day for 7 days, only removing for bathing. A diary is also provided to record what activities are done and how long for. The monitors, belt and diary will be posted back on completion. The Actigraph (GT3X + Manufacturing Technology Inc., Fl. USA) measures vertical accelerations in magnitudes from 0.05–2.0 g sampled at 30 Hz then summed over a selected (5 s) time period, it can record PA continuously for up to 21 days. The output, activity counts per unit of time, distinguishes between different walking speeds and PA intensities, using standard cut-offs [42,43]. The pedometer function on the accelerometer will be used for baseline and outcome measurement of step-counts for the trial. Participants will be offered the option of text messaging to remind them to wear the accelerometer each day and to return it after the 7 days. Once it is returned, the participants receive a £10 gift voucher.

Randomisation procedure

After all participants in a household have completed the baseline assessment and returned the accelerometer with at least 5 complete days of ≥9 hours / 540 minutes recording, the RA will allocate to the trial groups using an internet randomisation service to ensure independence of the allocation. Participants who do not provide the required data, will be asked to wear the accelerometers for another 7 days or excluded, if this is not possible. To avoid couple contamination, randomisation will be at household level. Block randomisation will be used within practice with random sized blocks to ensure balance in the groups and an even workload for nurses. The research assistant will inform participants by telephone of their group allocation.

Nature of the complex intervention

Twelve-week pedometer-based walking intervention delivered either by post with written instructions (pedometer group) or delivered in the context of three practice nurse PA consultations (pedometer plus nurse support group). Table 3 provides details of the complex intervention components. (Figure 2)

Procedure for control group (usual PA)

The research assistant informs participants that they are in the usual PA group and that they should continue with their usual PA throughout the trial. She/he will thank them for participating and inform them that they will be contacted later to arrange the 3-month postal assessment and the 12-month outcome assessment appointment at the practice, including wearing an accelerometer for 7 days as part of these. He/she will also make contact at 6 and 9 months (by telephone, text, or email according to patient preference) to check on safety outcomes and contact details. On study completion, the control group will be offered a pedometer, diary and written instructions for a 12-week pedometer-based walking programme either by post or as part of a single practice nurse consultation (according to patient preference).

Procedure for the pedometer-alone group

The research assistant informs participants that they are in the pedometer-alone group and arranges to post out a pedometer, PACE-UP patient handbook and diary with easy to follow written instructions for a 12-week pedometer-based walking programme. This is based on the participant's own baseline pedometer average daily step-count. The research assistant will telephone 1 week after sending out the pedometer to check that it has arrived safely and is working properly and to offer a replacement pedometer in the event of loss or malfunction during the 12-week intervention. He/she will also check that participants understand the 12-week pedometer-based walking plan and answer any questions. Arrangements for follow-up at 3, 6, 9 and 12 months are as for the control group. In addition, at each follow-up, the research assistant will offer a replacement pedometer or batteries, if required. On study completion, participants in this group will be offered a single practice nurse PA consultation.

Procedure for the pedometer-plus-nurse-support group

The research assistant informs participants that they are in the pedometer-plus-nurse-support group and arranges a practice nurse appointment for their first PA consultation. Participants can be seen individually or as a couple, for couples both individual goals and opportunities to increase their PA together will be discussed. Arrangements for follow-up at 3, 6, 9 and 12 months are as for the pedometer-alone group.

For peer review only - http://bmjopen.bmj.com/site/about/guidelines.xhtml

Harris et al. Trials 2013, 14:418
http://www.trialsjournal.com/content/14/1/418

Table 3 Components of the complex intervention for the PACE-UP trial

Components	What was provided	Group receipt of components	Additional detail on components
Pedometer	Yamax Digi-Walker (Tokyo, Japan) SW-200 model	Pedometer by post group (sent by post with instructions).<br>Pedometer plus support group (given by nurse to patients with instructions).	Yamax Digi-Walker is the criterion pedometer with best accuracy [72-74]. The CW200 model is used for baseline target setting, because of 7-day memory of consecutive daily steps, but is bulky to wear and complicated to use. For the intervention groups we are using the SW-200 model, which is compact, cheaper and simpler. It provides direct step-count to participants and requires daily manual recording and re-setting.
Patient handbook, walking plan and diary	Patient handbook to support 12-week walking programme. Suggested individualised walking plan (Figure 2). Diary to record weekly PA for 12 weeks (step-count and walks) and whether walking targets have been met each week.	Pedometer by post group (sent by post).<br>Pedometer plus support group (given by nurse to patients).	Participants' baseline average daily step-count (from blinded pedometer assessment) is recorded in the individual's handbook and diary. Participants have been informed that adding in 3,000 steps/day (approximately equivalent to a 30-minute brisk walk) on 5 or more days weekly to their baseline would help them achieve the recommended PA guidelines, but that this can be built up gradually. The handbook provides advice on the health benefits of at least moderate intensity PA and states that moderate intensity PA makes you warm and a bit breathless and increases your heart rate, but that you should still be able to talk. The handbook and diary provide written advice on maintaining activity, and anticipating and managing setbacks. Table 1 lists the BCTs [58] included in the PACE-UP patient handbook and diary, respectively.
Practice nurse PA consultations	Three individually tailored PA consultations with the practice nurse. Participants can be seen individually or as a couple.	Pedometer plus support group only.	Session timings, content and planned BCTs [58] (Table 2). Most BCTs overlap with those in the patient handbook and diary to reinforce consultations. The face-to-face nurse consultation allows some additional BCTs to be used; e.g., communication strategies to overcome resistance and promote patient-led change using motivational interviewing techniques and a scale to check confidence levels and build confidence to make change. In the first consultation, the nurse provides the pedometer, patient handbook and diary. The patient's baseline blinded pedometer average daily step-count is reviewed alongside health and anthropometric data, so that an individual PA plan, tailored to baseline step-count, abilities, health and goals and based on increasing walking and walking speed and other existing PA, can be produced. The nurse shows participants how to use the pedometer and how to record step-counts. Individual tailoring of step-count increase and how fast to increase this is possible. Participants are asked to wear a pedometer and keep daily step-count diary for 4 weeks, until their next appointment. If goals have been achieved new goals can be set, if not, then problems and barriers can be discussed. For couples, both individual goals and opportunities to increase their PA together will be discussed.

Procedure for qualitative study

The qualitative researcher will approach the non-participants and the participants, from both intervention groups, as discussed in qualitative participant recruitment and seek their informed consent for a semi-structured telephone interview. All interviews will be audio-recorded (unless participants do not consent, when contemporaneous field notes will be taken) and transcribed verbatim professionally. Thematic analysis will proceed in parallel with the interviews to enable refinement of the interview guide and purposive sampling according to emerging themes. The qualitative research assistant will also run a focus group with the practice nurses, when all the interventions are completed,

For peer review only

PACE-UP

The plan is to start from where you are currently and to gradually increase the amount you walk over 12 weeks.

Use the pedometer to record the number of steps you do each day and write them in your PACE-UP diary.

Weeks of PACE-UP walking programme	Target number of steps
1-2	Add in 1500 steps on 3 or more days per week
3-4	Add in 1500 steps on 5 or more days per week
5-6	Add in 3000 steps on 3 or more days per week
7-12	Add in 3000 steps on 5 or more days per week

Remember
1500 steps equals about 15 minutes of walking &
3000 steps equals about 30 minutes of walking

What does this mean for you?

From the pedometer worn at baseline your average number of daily steps was ....

Your 12 week programme will be as follows:

Add in extra steps to your baseline average of ....steps per day. Record your daily step-count on the PACE-UP diary sheets.

First month add in 1500 steps per day (which is about equal to a 15 minute walk), gradually increasing from 3 to 5 days per week

Second month add in 3000 steps per day (which is about equal to a 30 minute walk) gradually increasing from 3 to 5 days per week

Third month is maintenance, keep on adding in 3000 steps per day (about equal to a 30 minute walk) on at least 5 days per week.

By the end of 12 weeks the aim is for you to be walking an extra 3000 steps most days of the week. If you can do this, your average number of daily steps should have increased to about .... steps.

Figure 2 Provides a summary of the PACE-UP walking programme.

For peer review only

this will be audio-recorded and transcribed and subjected to thematic analysis.

Procedure for the health economics evaluation

The economic evaluation will take the perspective of the National Health Service personal social services and participants and first undertake a trial based analysis. Participant-level resource use data will be collected for equipment (pedometers), face to face or telephone consultations (length of time and frequency), out of pocket expenses (e.g., transport costs), use of support services (number of calls and contacts by post) and for other health service use (e.g., GP attendances, in-patient days, out-patient visits, home visits and services from social services, stays in nursing and residential care). Data will be collected through primary care records, participant questionnaire at 3 and 12 months and monitoring by nurses. Where possible, data collection procedures for the health economics evaluation will be carried out at the same time as those for study effectiveness. Costs that do not vary by use (e.g., development, production and translation of leaflets) will be estimated separately

and apportioned to patients within the relevant arm of the trial. Unit costs will be valued using national averages to increase their generalizability. Long-term costs and effects expected to occur beyond the trial will be estimated using Anokye et al.'s model, which accounts for the lifetime risk of developing three conditions associated with PA (coronary heart disease, stroke and type II diabetes) [75].

Practice nurse training and assessment of fidelity of practice nurse consultations

Practice nurse training in BCTs and in the use of the PACE-UP nurse handbook and PACE-UP patient handbook and diary will be planned with and conducted by experienced trainers in BCTs with primary care and practice nurse training experience (LD and DB) [56]. They will also provide supervision and monitoring to the nurses over the course of the trial, including listening to audio-recordings of a sample of each nurse's consultations and providing individual feedback. In addition, the Chief Investigator will provide training to the nurses on PA and safety aspects of the trial and the use of pedometers. Nurses will all go on a walk wearing an accelerometer to try out different walking speeds and be shown accelerometer feedback to appreciate the difference between light, moderate and vigorous PA intensities.

The fidelity and quality of the implementation of the intervention will be monitored over time and between different nurses by the following methods: i) analysing the content of a sample of audiorecorded sessions for each nurse by the trainers according to an agreed proforma (to include at least one example of each session and one example of a couple consultation); ii) discussion about consultations during group supervision/training with all the nurses; iii) completion of a checklist of areas covered in each consultation by the nurse; and iv) completion of a nurse patient alliance questionnaire at the end of each patient's intervention by both the nurse and the patient. The nurse patient alliance questionnaire was drawn up using a modified version of the Working Alliance Inventory [76,77] a validated measure of alliance frequently used in cognitive behavioural therapy based studies, and questions on patient self-efficacy adapted from the SCI Exercise Self-Efficacy Scale [78].

Assessment of outcomes after 3 and 12 months in the intervention and control groups

3-month postal assessment (interim assessment)

As for baseline assessment (including accelerometer assessment) but there is no anthropometric assessment, and the questionnaire has additional questions about adverse events, injuries and health problems over the last 3 months for all participants and questions on time and financial costs associated with PA and attending nurse appointments for the intervention groups as part of the health economics assessment.

12-month assessment at the patient's general practice (primary outcome assessment)

As for baseline assessment (including accelerometer assessment) but questionnaire has additional questions about adverse events, including injuries and health problems and use of pedometer over the last 12 months (for pedometer use, slightly different questions depending on group).

Accelerometer data will be downloaded as soon as each accelerometer is returned. Data entry of questionnaire data will occur as soon as possible after data collection at each period. Analysis of outcome data will occur when data on all participants is complete.

Outcome measures

The primary outcome is change in average daily step-count, measured over 7 days, between baseline and 12 months assessed objectively by accelerometry (Actigraph GT3X + Manufacturing Technology Inc., FL, USA).

Secondary outcomes are:

i) Change in time spent in at least moderate intensity PA and in time spent sedentary between baseline and 12 months, measured over 7 days by accelerometry.
ii) Change in average daily step-count, time spent in at least moderate PA and time spent sedentary measured over 7 days, between baseline and 3 months by accelerometry.
iii) Cost-effectiveness. Incremental cost of the intervention to the National Health Service and incremental cost per change in step-count and per quality adjusted life year.

Other ancillary outcome measures:

i) Change in self-reported PA assessed by GPPAQ and IPAQ.
ii) Change in other patient reported outcomes from the questionnaire (exercise self-efficacy, anxiety, depression, EQ-5D).
iii) Change in anthropometric measurements; weight, BMI, waist circumference, body fat, bioimpedance.
iv) Adverse outcomes; data on falls, injuries, major cardiovascular disease events and deaths will be collected as part of safety monitoring for the trial, through participant and nurse reporting, questionnaires at 3 and 12 months and primary care records after 12 month follow-up.
v) Health service use – number of and diagnoses for all primary care consultations during the 12 months of the trial, as well as any out of hours, A & E, or

For peer review only - http://bmjopen.bmj.com/site/about/guidelines.xhtml

in-patient attendances that lead to new diagnoses recorded in computerised primary care records, downloaded at the end of the study, given participants' consent.

Qualitative outcomes

There will be a range of outcomes from qualitative interviews and focus groups for non-participants, participants and practice nurses involved in implementing the intervention. We will gain an in-depth understanding of the acceptability and challenges with the interventions for participants and practice nurses, as well as valuable insights into the factors influencing why people opt not to participate in the intervention.

Sample size

A meta-analysis of a heterogeneous group of short-term intervention studies involving pedometers showed interventions increased steps count per day by 2,500 with a SD of 2,700 [23]. However, a smaller increase in steps of 1,000 per day would lead to worthwhile health gains if this was sustained for 12 months. We also want to be able to demonstrate whether there are differences in the effects achieved by a pedometer intervention alone compared with a pedometer intervention with nurse support. A sample of 217 patients in each of three arms would allow a difference of 1,000 steps per day to be detected between any two arms of the trial with a 90% power at the 1% significance level. This means that we will have sufficient power to adjust for multiple hypothesis testing. However, we plan to randomise households. For men and women the effect of clustering is likely to be small but needs to be taken into account when stratifying by age. Assuming an intra-cluster correlation of 0.5 and an average household size of 1.6 eligible patients we would need to analyse 282 patients per arm. Allowing for approximately 15% attrition, we would need to randomise a total of 993 patients (331 usual PA, 331 pedometer only and 331 pedometer plus nurse support). Six general practices (centres) each recruiting approximately 166 patients will suffice. We will select patients at random to take part until required numbers have been randomised.

Anticipated recruitment

We anticipate a recruitment rate of 20% amongst those eligible to participate. This estimate is based on pilot work using pedometers and accelerometers in an observational study of older primary care patients, recruitment rate 43% [42] and other studies of PA interventions (including with pedometers) amongst middle-aged and older adults in primary care, where recruitment has been between 6% and 35% [25,79-83]. Even if our recruitment rate were as low as 10%, we would have enough eligible participants (Figure 1).

Statistical analysis

Analysis and reporting will be in line with CONSORT guidelines, with primary analyses being on an intention-to-treat basis. That is, all participants will be included who have outcome data, regardless of their adherence to the interventions. Sensitivity analyses including all randomised patients will be carried out using multiple imputation to impute PA levels at 12 months for subjects randomised but with no adequate accelerometry data at 12 months; baseline data are available for all subjects by definition. All participants will be included in the primary analysis if they have at least one satisfactory day of accelerometer recording out of 7 days at 12-month follow-up. A satisfactory recording comprises at least 540 minutes (9 hours) of registered time during a day. Adequacy of the randomisation process to achieve balanced groups will be checked by comparing participant characteristics in the three arms (e.g., age, sex, socio-economic group, baseline PA level, health status, body mass index, household size). The same variables will be compared between those who complete follow-up and those who drop out completely, and those who fail to provide a complete set of 5 days data for the primary outcome. Significance tests, either *t*-test or χ^2 tests, will be used to compare those with complete data and those who have missing outcomes.

Primary analysis

The primary outcome measure is change in step-count from baseline to 12-month follow-up. Secondary outcome measures which we will also examine are counts per minute, counts per minute of registered time and number of minutes spent in moderate or vigorous PA. These measures are likely to be highly correlated with step count and will be analysed using identical approaches to that for step count. The primary analysis will use all patients with at least 1 day of adequate accelerometry data at 12 months (i.e., complete case analysis). The main outcome will be the change in average daily step-count measured over 7 days between baseline and 12 months. In practice, we will regress average daily step-count at 12 months on average baseline steps per day; this will effectively be measuring change in number of steps over the 12 months.

Subsidiary analyses

Subsidiary analysis will investigate whether there is any evidence of interaction, that is whether the treatment effect varies by the following factors: age (<60 versus ≥60), gender, socio-economic group, ethnic group, participating as a couple, disability, health status, BMI and exercise self-efficacy. Numbers in each group who have suffered a fracture, falls and injuries, and dropouts will be compared between the groups using logistic regression in STATA, adjusted for clustering.

Harris et al. Trials 2013, 14:418
http://www.trialsjournal.com/content/14/1/418

Stopping rules

It would be impossible to carry out interim analyses on sufficient patients to decide to stop, so there are no formal statistical stopping rules. If a patient becomes ineligible, the nurse may discontinue the intervention, but all patients will be asked to complete follow-up assessments. Patients can withdraw at any time.

Procedure for accounting for missing data

Only days with at least 540 minutes of registered time on accelerometer on a given day will be used. Participants in all groups with less than three days satisfactory wear time at follow-up will be asked to wear the accelerometer for an addidional week and the second set of readings used if greater wear time. Participants will only be randomized if they provide at least five such days of accelerometer data at baseline. We will use a mixed effects multilevel linear regression model of daily step count, taking account of day of the week and days since start of measurement to estimate the baseline average daily steps for each subject. The main analysis of effect will include all subjects with at least one satisfactory day of recording at 12 months. We will estimate average daily steps at 12 months for each subject using an identical approach to that at baseline; we will then regress estimated PA level at 12 months on estimated PA level at baseline, age, sex and practice as well as treatment group, while including household as a random effect. In a further sensitivity analysis, we will use multiple imputation to impute values for those with no accelerometer data at 12 months.

Participant withdrawal

Participants will be free to withdraw from the trial at any time and without giving a reason. Practice nurses can advise discontinuation of the PA intervention if the intervention poses a hazard to the participant. In both cases, information that has already been collected on participants may still be used and they will be asked if they would be prepared to provide any further data on outcomes at 3 months and 12 months (e.g., questionnaire, anthropometric measurements and/or PA monitoring). Withdrawal from the study will not affect the standard of care received by the practice. If participants withdraw before they have been randomised they will be replaced, those withdrawing or being withdrawn after randomisation will not be replaced.

Adverse event monitoring

Notification and reporting of adverse events

A standard operating procedure for the management of adverse events will be in place, so that participants or their relatives, practice staff or researchers can inform the chief investigator of any event. All adverse events reported will be assessed for seriousness, expectedness and causality.

Retrospective data collection on adverse events

i) *Questionnaires:* Intervention and control groups will be sent questionnaires at 3 and 12 months that will ask specifically about falls, injuries and exacerbation of any pre-existing conditions in the previous 3- and 12-month periods, respectively.
ii) *Contact with research assistant:* Participants in all three groups will be contacted at 6 and 9 months (by telephone, text or email as preferred by participant) and asked about adverse events since the last contact.
iii) *Computerised primary care records:* In order to be sure that full data on adverse events is collected, informed consent will be sought to collect data from participant records at the end of the study. All consultation data for the 12-month period of the study for each individual will be downloaded from practice computerised records, including all new problems/diagnoses recorded during this period. This will be anonymised before removal from the practice and a researcher who is blind to the intervention or control status of the participants will analyse this data with a standardised proforma recording possible adverse events.

Ethical and organizational review

The trial has been reviewed and given a favourable opinion by the London Research Ethics Committee (Hampstead) (12/LO/0219). National Health Service Research and Development approval was given initially by Primary Care Trusts and then by Clinical Commissioning Groups in South West London to cover all the practice sites.

Discussion

The PACE-UP trial is a primary care based PA intervention for inactive 45–75 year olds which seeks to discover if provision of a pedometer by post as part of a 12-week walking programme can increase PA levels at 12 months compared with usual care and whether additional practice nurse PA consultations can increase any effects. It is a pragmatic trial being conducted across several general practices with patients' own practice nurses, rather than trained researchers or therapists delivering the intervention. The findings will therefore be of direct relevance to UK primary care and other developed countries with similar healthcare provision.

For peer review only

We have taken the following measures in the trial to minimise or avoid bias:

i) *Randomisation:* The Kings College Clinical Trials Unit internet randomisation service will be used to ensure allocation concealment. Randomisation will be at household level to avoid couple contamination (see below).
ii) *Contamination:* Contamination could occur between partners in the same household. This will be minimised by ensuring that if both are recruited they are allocated to the same group. Contamination could also occur in the control 'usual PA' group if they seek to increase their PA. Participants will be discouraged from buying a pedometer by ensuring that they know that they will receive one, along with instructions on its use and the offer of a PA consultation with their practice nurse and feedback on their individual activity levels at the end of the trial. The 3-month and 12-month assessments will capture information on PA in the usual PA group, including a question at 12 months about whether they have used a pedometer at all in the previous year.
iii) *Blinding and assessment of outcomes:* Participants cannot be blinded to their intervention or control status. The research assistants assessing outcomes will not be blinded to the participants' intervention status for pragmatic reasons; the study is funded to support only two research assistants to carry out recruitment and follow-up simultaneously at their allocated practices. Appointments for the 3-month and 12-month outcome assessments will be booked in advance according to a protocol, taking into account holidays. However, primary and secondary outcome measures are objectively measured by accelerometry and do not rely on assessor interpretation. Physical measurements will also be assessed objectively (e.g., body weight and body fat measurements using scales with print-out results). Patient reported outcomes will be assessed by validated self-report instruments, minimising researcher bias. The statistician analysing the data will be blind to the treatment allocation of the participants.

The particular challenges that we anticipate in this study are as follows:

i) Low levels of recruitment and possible selection bias, with those who are more physically active being more likely to want to take part. We have a screening question to filter out those who already report recommended PA levels, this should minimize the number who are too active taking part. We are addressing potential low levels of recruitment by recruiting from practices with enough people in the target age range for us to achieve our sample size even if recruitment were as low as 10% of those eligible. In order to estimate response bias we aim to assess self-reported PA and health on those who are not recruited to the trial, but who are willing to fill out a short questionnaire.
ii) Variation in the PA interventions delivered across practices and over time. We have several quality assurance mechanisms in place (including protocols for research assistants who are delivering the postal intervention, and protocols, audio-recording of consultations, group supervision, nurse checklists and patient nurse alliance scales for the nurses delivering the PA consultations) to help us to avoid and monitor these aspects of fidelity.
iii) Losses to follow-up, particularly the control group. We hope to reduce this in the following ways: personal contact with the same research assistant; the offer of a £10 gift voucher when accelerometers are returned; offering controls individual feedback on their activity levels after they complete the trial from their baseline, 3-month and 12-month assessments; and offering a pedometer and 12-week individualized walking programme, either by post or in a single nurse PA consultation, after trial completion.

The findings of this trial will contribute importantly to the development of strategies to address a key global public health challenge, low PA among adults and older adults. Specifically in the UK, an understanding of the role of pedometer-based programmes and nurse support will help guide national policy on promoting PA in primary care. If effective and cost-effective, our interventions could be incorporated into the National Health Service Health Check Programme, which targets patients aged 40–74 years. More widely, our findings will be able to guide international policy and recommendations for increasing PA.

Trial status

In recruitment phase (recruitment started October 2013 and anticipated to finish November 2013).

Abbreviations

BCI: Behaviour change intervention; BCTs: Behaviour change techniques; BMI: Body mass index; GP: General practitioner; NICE: National Institute for Health and Clinical Excellence; PA: Physical activity; RCT: Randomised controlled trial; UK: United Kingdom.

Competing interests

The authors declare that they have no competing interests.

For peer review only - http://bmjopen.bmj.com/site/about/guidelines.xhtml

Authors' contributions

TH, DC, CV and SK conceived the idea for the study. TH, DC, CV, SK, SS, SI, MU, UE, PW, JF-W and NA participated in the design of the study and developed the research protocol for funding. SK, DC and EL were responsible for the statistical analysis plan, and SK and DC for the sample size calculations. CV and TH designed the qualitative aspects of the study. TH, LD, DB and MU designed the behaviour change intervention, adapted the National Health Service health trainer handbook for the purposes of this trial and designed the patient handbook and patient diary. TH, LD, DB, MU and CF designed and carried out the nurse training. JF-W and NA designed the health economics procedures and data collection tools. CF, EH and RD were involved in compiling patient information and data collection packs and in discussions of any practical changes required to the protocol. EL organised the random samples for each of the practices and data collection and data management plans. TH, JI, SDW, MU and SS were involved in questionnaire development and practice recruitment, selection and training. All the authors have read and approved the final manuscript.

Acknowledgements

This project was funded by the National Institute for Health Research Health Technology Assessment (NIHR HTA) Programme (project number HTA 10/32/02) and will be published in full in Health Technology Assessment. The views and opinions expressed therein are those of the authors and do not necessarily reflect those of the HTA programme, NIHR, National Health Service, or the Department of Health. The funding body was not involved in the study design, collection of data, analysis and interpretation of data, writing of the manuscript or the decision to submit the manuscript for publication. We would like to thank the general practices and their practice nurses who supported this study: Upper Tooting Road Practice, Tooting; Chatfield Practice, Battersea; Wrythe Green Practice, Carshalton; Francis Grove Practice, Wimbledon; Putneymead and Heathfield Practices Putney; and Cricket Green Practice, Mitcham, and all the patients who participated.

Author details

[1]Population Health Research Centre, St George's University of London, London SW17 ORE, UK. [2]Pragmatic Clinical Trials Unit, Queen Mary's University of London, London E12AT, UK. [3]Gerontology and Health Services Research Unit, Brunel University, London UB8 3PH, UK. [4]Department of Population Health Sciences, University College, London NW3 2PF, UK. [5]MRC Epidemiology Unit, University of Cambridge, Cambridge CB2 OQQ, UK. [6]Department of Sport Medicine, Norwegian School of Sport Sciences, PO Box 4014, 0806 Oslo, Norway. [7]Health Economics Research Group, Brunel University, London UB83PH, UK. [8]10 Minute CBT, Devonshire Business Centre, Letchworth Garden City, Herts SG61GJ, UK.

Received: 9 September 2013 Accepted: 20 November 2013
Published: 5 December 2013

References

1. Department of Health PAHI&P: *Start Active, Stay Active: A Report on Physical Activity for Health from the Four Home Countries' Chief Medical Officers.* London: UK Department of Health; 2011.
2. World Health Organisation: *Global Recommendations on Physical Activity for Health.* Geneva: WHO; 2010.
3. Garber CE, Blissmer B, Deschenes MR, Franklin BA, Lamonte MJ, Lee IM, Nieman DC, Swain DP, American College of Sports Medicine: **American college of sports medicine position stand: quantity and quality of exercise for developing and maintaining cardiorespiratory, musculoskeletal, and neuromotor fitness in apparently healthy adults: guidance for prescribing exercise.** *Med Sci Sports Exerc* 2011, **43:**1334–1359.
4. O'Donovan G, Blazevich AJ, Boreham C, Cooper AR, Crank H, Ekelund U, Fox KR, Gately P, Giles-Corti B, Gill JM, Hamer M, McDermott I, Murphy M, Mutrie N, Reilly JJ, Saxton JM, Stamatakis E: **The ABC of physical activity for health: a consensus statement from the British association of sport and exercise sciences.** *J Sports Sci* 2010, **28:**573–591.
5. Morris JN, Hardman AE: **Walking to health.** *Sports Med* 1997, **23:**306–332.
6. Joint Health Surveys Unit: *Health Survey for England 2008: Physical Activity & Fitness.* London: The NHS Information Centre for Health & Social Care; 2009.
7. Simonsick EM, Guralnik JM, Volpato S, Balfour J, Fried LP: **Just get out the door! Importance of walking outside the home for maintaining mobility: findings from the women's health and aging study.** *J Am Geriatr Soc* 2005, **53:**198–203.
8. National Centre for Social Research: *Health Survey for England 2004.* London: Department of Health; 2005.
9. Sjostrom M, Oja P, Hagstromer M, Smith BJ, Bauman A: **Health-enhancing physical activity across the European union countries: the eurobarometer study.** *J Public Health* 2006, **14:**291–300.
10. Centers for Disease Control and Prevention: *State Indicator Report on physical Activity.* Atlanta GA: US Department of Health and Human Services; 2010.
11. Tudor-Locke CE, Myers AM: **Challenges and opportunities for measuring physical activity in sedentary adults.** *Sports Med* 2001, **31:**91–100.
12. Morey MC, Sullivan RJ Jr: **Medical assessment for health advocacy and practical strategies for exercise initiation.** *Am J Prev Med* 2003, **25:**204–208.
13. Cress ME, Buchner DM, Prohaska T, Rimmer J, Brown M, Macera C, Dipietro L, Chodzko-Zajko W: **Best practices for physical activity programs and behavior counseling in older adult populations.** *J Aging Phys Act* 2005, **13:**61–74.
14. Thompson PD, Buchner D, Pina IL, Balady GJ, Williams MA, Marcus BH, Berra K, Blair SN, Costa F, Franklin B, Fletcher GF, Gordon NF, Pate RR, Rodriguez BL, Yancey AK, Wenger NK, American Heart Association Council on Clinical Cardiology Subcommittee on Exercise, Rehabilitation, and Prevention; American Heart Association Council on Nutrition, Physical Activity, and Metabolism Subcommittee on Physical Activity: **Exercise and physical activity in the prevention and treatment of atherosclerotic cardiovascular disease: a statement from the council on clinical cardiology (subcommittee on exercise, rehabilitation, and prevention) and the council on nutrition, physical activity, and metabolism (subcommittee on physical activity).** *Circulation* 2003, **107:**3109–3116.
15. Hootman JM, Macera CA, Ainsworth BE, Martin M, Addy CL, Blair SN: **Association among physical activity level, cardiorespiratory fitness, and risk of musculoskeletal injury.** *Am J Epidemiol* 2001, **154:**251–258.
16. Ory M, Resnick B, Jordan PJ, Coday M, Riebe D, Ewing Garber C, Pruitt L, Bazzarre T: **Screening, safety, and adverse events in physical activity interventions: collaborative experiences from the behavior change consortium.** *Ann Behav Med* 2005, **29:**20–28.
17. Hillsdon M, Foster C, Thorogood M: **Interventions for promoting physical activity.** *Cochrane Database Syst Rev* 2005, **1:**CD003180.
18. Hillsdon M, Foster C, Cavill N, Crombie H, Naidoo B: *The Effectiveness of Public Health Interventions for Increasing Physical Activity Among Adults: A Review of Reviews.* London: HAD; 2005.
19. Hobbs N, Godfrey A, Lara J, Errington L, Meyer TD, Rochester L, White M, Mathers JC, Sniehotta FF: **Are behavioral interventions effective in increasing physical activity at 12 to 36 months in adults aged 55 to 70 years? A systematic review and meta-analysis.** *BMC Med* 2013, **11:**75.
20. King AC, Rejeski WJ, Buchner DM: **Physical activity interventions targeting older adults: a critical review and recommendations.** *Am J Prev Med* 1998, **15:**316–333.
21. National Institute for Health and Clinical Excellence: *Behaviour Change at Population, Community and Individual Levels.* London: National Institute of Health and Clinical Excellence; 2007.
22. Ogilvie D, Foster CE, Rothnie H, Cavill N, Hamilton V, Fitzsimons CF, Mutrie N: **Scottish Physical Activity Research Collaboration: Interventions to promote walking: systematic review.** *BMJ* 2007, **334:**1204.
23. Bravata DM, Smith-Spangler C, Sundaram V, Gienger AL, Lin N, Lewis R, Stave CD, Olkin I, Sirard JR: **Using pedometers to increase physical activity and improve health: a systematic review.** *JAMA* 2007, **298:**2296–2304.
24. Kang M, Marshall SJ, Barreira TV, Lee JO: **Effect of pedometer-based physical activity interventions: a meta-analysis.** *Res Q Exerc Sport* 2009, **80:**648–655.
25. McMurdo ME, Sugden J, Argo I, Boyle P, Johnston DW, Sniehotta FF, Donnan PT: **Do pedometers increase physical activity in sedentary older women? A randomized controlled trial.** *J Am Geriatr Soc* 2010, **58:**2099–2106.
26. Houle J, Doyon O, Vadeboncoeur N, Turbide G, Diaz A, Poirier P: **Innovative program to increase physical activity following an acute coronary syndrome: randomized controlled trial.** *Patient Educ Couns* 2011, **85:**e237–e244.
27. Yates T, Davies M, Gorely T, Bull F, Khunti K: **Effectiveness of a pragmatic education program designed to promote walking activity in individuals with impaired glucose tolerance: a randomized controlled trial.** *Diabetes Care* 2009, **32:**1404–1410.

For peer review only

28. National Institute for Health and Clinical Excellence: *Four Commonly Used Methods to Increase Physical Activity: Brief Interventions in Primary Care, Exercise Referral Schemes, Pedometers and Community Based Exercise Programmes for Walking and Cycling. Public Health Intervention Guidance no.2.* London: National Institute for Health and Clinical Excellence; 2006.
29. National Institute for Health and Clinical Excellence: *Walking and Cycling: Local Measures to Promote Walking and Cycling as Forms of Travel or Recreation. NICE Public Health Guidance 41.* London: National Institute for Health and Clinical Excellence; 2012.
30. Schneider PL, Bassett DR Jr, Thompson DL, Pronk NP, Bielak KM: **Effects of a 10,000 steps per day goal in overweight adults.** *Am J Health Promot* 2006, **21:**85–89.
31. Lindberg R: **Active living: on the road with the 10,000 Steps program.** *J Am Diet Assoc* 2000, **100:**878–879.
32. Croteau KA, Richeson NE, Farmer BC, Jones DB: **Effect of a pedometer-based intervention on daily step counts of community-dwelling older adults.** *Res Q Exerc Sport* 2007, **78:**401–406.
33. Prochaska JJ, Hall SM, Humfleet G, Munoz RF, Reus V, Gorecki J, Hu D: **Physical activity as a strategy for maintaining tobacco abstinence: a randomized trial.** *Prev Med* 2008, **47:**215–220.
34. Tudor-Locke C, Craig CL, Brown WJ, Clemes SA, De CK, Giles-Corti B, Hatano Y, Inoue S, Matsudo SM, Mutrie N, Oppert JM, Rowe DA, Schmidt MD, Schofield GM, Spence JC, Teixeira PJ, Tully MA, Blair SN: **How many steps/day are enough? For adults.** *Int J Behav Nutr Phys Act* 2011, **8:**79.
35. Marshall SJ, Levy SS, Tudor-Locke CE, Kolkhorst FW, Wooten KM, Ji M, Macera CA, Ainsworth BE: **Translating physical activity recommendations into a pedometer-based step goal: 3000 steps in 30 minutes.** *Am J Prev Med* 2009, **36:**410–415.
36. McKay J, Wright A, Lowry R, Steele K, Ryde G, Mutrie N: **Walking on prescription: the utility of a pedometer pack for increasing physical activity in primary care.** *Patient Educ Couns* 2009, **76:**71–76.
37. Baker G, Gray SR, Wright A, Fitzsimons C, Nimmo M, Lowry R, Mutrie N, Scottish Physical Activity Research Collaboration (SPARColl): **The effect of a pedometer-based community walking intervention "Walking for Wellbeing in the West" on physical activity levels and health outcomes: a 12-week randomized controlled trial.** *Int J Behav Nutr Phys Act* 2008, **5:**44.
38. Tudor-Locke C, Craig CL, Aoyagi Y, Bell RC, Croteau KA, De Bourdeaudhuij I, Ewald B, Gardner AW, Hatano Y, Lutes LD, Matsudo SM, Ramirez-Marrero FA, Rogers LQ, Rowe DA, Schmidt MD, Tully MA, Blair SN: **How many steps/day are enough? For older adults and special populations.** *Int J Behav Nutr Phys Act* 2011, **8:**80.
39. Macmillan F, Fitzsimons C, Black K, Granat MH, Grant MP, Grealy M, Macdonald H, McConnachie A, Rowe DA, Shaw R, Skelton DA, Mutrie N: **West End Walkers 65+: a randomised controlled trial of a primary care-based walking intervention for older adults: study rationale and design.** *BMC Public Health* 2011, **11:**120.
40. Mutrie N, Doolin O, Fitzsimons CF, Grant PM, Granat M, Grealy M, Macdonald H, MacMillan F, McConnachie A, Rowe DA, Shaw R, Skelton DA: **Increasing older adults' walking through primary care: results of a pilot randomized controlled trial.** *Fam Pract* 2012, **29**(6):633–642.
41. Ekelund U, Tingstrom P, Kamwendo K, Krantz M, Nylander E, Sjostrom M, Bergdahl B: **The validity of the Computer Science and Applications activity monitor for use in coronary artery disease patients during level walking.** *Clin Physiol Funct Imaging* 2002, **22:**248–253.
42. Harris TJ, Owen CG, Victor CR, Adams R, Cook DG: **What factors are associated with physical activity in older people, assessed objectively by accelerometry?** *Br J Sports Med* 2009, **43:**442–450.
43. Davis MG, Fox KR: **Physical activity patterns assessed by accelerometry in older people.** *Eur J Appl Physiol* 2007, **100:**581–589.
44. Harris TJ, Owen CG, Victor CR, Adams R, Ekelund U, Cook DG: **A comparison of questionnaire, accelerometer, and pedometer: measures in older people.** *Med Sci Sports Exerc* 2009, **41:**1392–1402.
45. Tudor-Locke C, Ainsworth BE, Thompson RW, Matthews CE: **Comparison of pedometer and accelerometer measures of free-living physical activity.** *Med Sci Sports Exerc* 2002, **34:**2045–2051.
46. NICE Public Health Collaborating Centre: *A Rapid Review of the Effectiveness of Pedometer Interventions to Promote Physical Activity in Adults.* London: National Institute for Health and Clinical Excellence; 2006.
47. Talib H, Sabirin J: *Pedometer and Accelerometer in Assessing Physical Activity Among Children and Adolescents.* Malaysia: Health Technology Assessment Unit; 2007.
48. NICE: *Modelling the Cost-Effectiveness of Physical Activity Interventions.* London: National Institute for Clinical Excellence; 2006.
49. Shaw R, Fenwick E, Baker G, McAdam C, Fitzsimons C, Mutrie N: **'Pedometers cost buttons': the feasibility of implementing a pedometer based walking programme within the community.** *BMC Public Health* 2011, **11:**200.
50. Health Checks Programme NHS: *Putting Prevention First: NHS Health Checks: Vascular Risk Assessment and Management Best Practice Guidelines.* London: NHS; 2009.
51. Lawton BA, Rose SB, Elley CR, Dowell AC, Fenton A, Moyes SA: **Exercise on prescription for women aged 40–74 recruited through primary care: two year randomised controlled trial.** *BMJ* 2008, **337:**a2509.
52. Brawley LR, Rejeski WJ, King AC: **Promoting physical activity for older adults: the challenges for changing behavior.** *Am J Prev Med* 2003, **25:**172–183.
53. Tai SS, Gould M, Iliffe S: **Promoting healthy exercise among older people in general practice: issues in designing and evaluating therapeutic interventions.** *Br J Gen Pract* 1997, **47:**119–122.
54. Goodman C, Davies S, Tai SS, Dinan S, Iliffe S: **Promoting older peoples' participation in activity, whose responsibility? A case study of the response of health, local government and voluntary organizations.** *J Interprof Care* 2007, **21:**515–528.
55. Khan KM, Weiler R, Blair SN: **Prescribing exercise in primary care.** *BMJ* 2011, **343:**d4141.
56. David L: *Using CBT in General Practice: The 10 Minute Consultation.* Oxfordshire: Scion; 2006.
57. British Psychological Society: *Improving Health: Changing Behaviour: NHS Health Trainer Handbook.* London: Department of Health; 2008.
58. Michie S, Ashford S, Sniehotta FF, Dombrowski SU, Bishop A, French DP: **A refined taxonomy of behaviour change techniques to help people change their physical activity and healthy eating behaviours: the CALO-RE taxonomy.** *Psychol Health* 2011, **26:**1479–1498.
59. Harris T, Kerry S, Victor CR, Ekelund U, Woodcock A, Iliffe S, Whincup P, Beighton C, Ussher M, David L, Brewin D, Adams F, Rogers A, Cook D: **Randomised controlled trial of a complex intervention by primary care nurses to increase walking in patients aged 60–74 years: protocol of the PACE-Lift (Pedometer accelerometer consultation evaluation - Lift) trial.** *BMC Public Health* 2013, **13:**5.
60. Boutron I, Moher D, Altman DG, Schulz KF, Ravaud P: **Methods and processes of the CONSORT Group: example of an extension for trials assessing nonpharmacologic treatments.** *Ann Intern Med* 2008, **148:**W60–W66.
61. Noble M, McLennan D, Wilkinson K, Whitworth A, Barnes H: *The English Indices of Deprivation 2007.* London: Department of Communities and Local Government; 2008.
62. Shah SM, Carey IM, Harris T, DeWilde S, Victor CR, Cook DG: **Do good health and material circumstances protect older people from the increased risk of death after bereavement?** *Am J Epidemiol* 2012, **176:**689–698.
63. Caspersen CJ, Bloemberg BP, Saris WH, Merritt RK, Kromhout D: **The prevalence of selected physical activities and their relation with coronary heart disease risk factors in elderly men: the Zutphen Study, 1985.** *Am J Epidemiol* 1991, **133:**1078–1092.
64. McGee MA, Johnson A, Kay D: **The medical research council cognitive functioning and ageing study (MRC CFAS): the description of activities of daily living in five centres in England and Wales.** *Age & Ageing* 1998, **27:**605–613.
65. Jette AM, Rooks D, Lachman M, Lin TH, Levenson C, Heislein D, Giorgetti MM, Harris BA: **Home-based resistance training: predictors of participation and adherence.** *Gerontologist* 1998, **38:**412–421.
66. Zigmond A, Snaith R: **The hospital anxiety and depression scale.** *Acta Psychiatr Scand* 1983, **67:**361–370.
67. Brooks R: **EuroQol: the current state of play.** *Health Policy* 1996, **37:**53–72.
68. Tunstall J: *Old and Alone.* London: Routledge and Kegan; 1957.
69. Department of Health: *General Practice Physical Activity Questionnaire.* London: UK Department of Health; 2006.
70. Booth ML: **Assessment of physical activity: an international perspective.** *Res Q Exerc Sport* 2000, **71:**s114–s120.
71. Nandy S, Parsons S, Cryer C, Underwood M, Rashbrook E, Carter Y, Eldridge S, Close J, Skelton D, Taylor S: **Feder G; Falls Prevention Pilot Steering Group: Development and preliminary examination of the predictive**

For peer review only

validity of the falls risk assessment tool (FRAT) for use in primary care. *J Public Health (Oxf)* 2004, **26**:138–143.

72. Bassett DR Jr, Ainsworth BE, Leggett SR, Mathien CA, Main JA, Hunter DC, Duncan GE: **Accuracy of five electronic pedometers for measuring distance walked.** *Med Sci Sports Exerc* 1996, **28**:1071–1077.
73. Schneider PL, Crouter SE, Bassett DR: **Pedometer measures of free-living physical activity: comparison of 13 models.** *Med Sci Sports Exerc* 2004, **36**:331–335.
74. Le Masurier GC, Lee SM, Tudor-Locke C: **Motion sensor accuracy under controlled and free-living conditions.** *Med Sci Sports Exerc* 2004, **36**:905–910.
75. Anokye N, Lord J, Fox-Rushby J, National Institute for Health and Clinical Excellence Public Health Intervention Guidance Physical Activity: *Brief Advice for Adults in Primary Care: Component 2 Economic Analysis: Economic Modelling of Brief Advice on Physical Activity for Adults in Primary Care.* London: National Institute for Health and Clinical Excellence; 2012.
76. Hovarth AO, Greenberg LS: **Development and validation of the working alliance inventory.** *J Counselling Psychol* 1989, **36**:223–233.
77. Andrusyna TP, Tang TZ, DeRubeis RJ, Luborsky L: **The factor structure of the working alliance inventory in cognitive-behavioral therapy.** *J Psychother Pract Res* 2001, **10**:173–178.
78. Kroll T, Kehn M, Ho P, Groah S: **The SCI Exercise Self-Efficacy Scale (ESES): development and psychometric properties.** *Int J Behav Nutr Phys Act* 2007, **4**:34.
79. Tully MA, Cupples ME, Chan WS, McGlade K, Young IS: **Brisk walking, fitness, and cardiovascular risk: a randomized controlled trial in primary care.** *Prev Med* 2005, **41**:622–628.
80. Little P, Dorward M, Gralton S, Hammerton L, Pillinger J, White P, Moore M, McKenna J, Payne S: **A randomised controlled trial of three pragmatic approaches to initiate increased physical activity in sedentary patients with risk factors for cardiovascular disease.** *Br J Gen Pract* 2004, **54**:189–195.
81. Stevens W, Hillsdon M, Thorogood M, McArdle D: **Cost-effectiveness of a primary care based physical activity intervention in 45–74 year old men and women: a randomised controlled trial.** *Br J Sports Med* 1998, **32**:236–241.
82. Munro JF, Nicholl JP, Brazier JE, Davey R, Cochrane T: **Cost effectiveness of a community based exercise programme in over 65 year olds: cluster randomised trial.** *J Epidemiol Community Health* 2004, **58**:1004–1010.
83. Sugden JA, Sniehotta FF, Donnan PT, Boyle P, Johnston DW, McMurdo ME: **The feasibility of using pedometers and brief advice to increase activity in sedentary older women–a pilot study.** *BMC Health Serv Res* 2008, **8**:169.

doi:10.1186/1745-6215-14-418
Cite this article as: Harris *et al.*: PACE-UP (Pedometer and consultation evaluation - UP) – a pedometer-based walking intervention with and without practice nurse support in primary care patients aged 45–75 years: study protocol for a randomised controlled trial. *Trials* 2013 **14**:418.

Submit your next manuscript to BioMed Central and take full advantage of:

- Convenient online submission
- Thorough peer review
- No space constraints or color figure charges
- Immediate publication on acceptance
- Inclusion in PubMed, CAS, Scopus and Google Scholar
- Research which is freely available for redistribution

Submit your manuscript at
www.biomedcentral.com/submit

BioMed Central

For peer review only

CHEERS Checklist

Items to include when reporting economic evaluations of health interventions

The **ISPOR CHEERS Task Force Report**, *Consolidated Health Economic Evaluation Reporting Standards (CHEERS)—Explanation and Elaboration: A Report of the ISPOR Health Economic Evaluations Publication Guidelines Good Reporting Practices Task Force,* provides examples and further discussion of the 24-item CHEERS Checklist and the CHEERS Statement. It may be accessed via the *Value in Health* or via the ISPOR Health Economic Evaluation Publication Guidelines – CHEERS: Good Reporting Practices webpage: http://www.ispor.org/TaskForces/EconomicPubGuidelines.asp

Section/item	Item No	Recommendation	Reported on page No/ line No
**Title and abstract**			
Title	1	Identify the study as an economic evaluation or use more specific terms such as "cost-effectiveness analysis", and describe the interventions compared.	P1 L1-2
Abstract	2	Provide a structured summary of objectives, perspective, setting, methods (including study design and inputs), results (including base case and uncertainty analyses), and conclusions.	P2 L1-39
**Introduction**			
Background and objectives	3	Provide an explicit statement of the broader context for the study. Present the study question and its relevance for health policy or practice decisions.	P4 all & P5L4-5
**Methods**			
Target population and subgroups	4	Describe characteristics of the base case population and subgroups analysed, including why they were chosen.	P4L1-21 & P2L11-12
Setting and location	5	State relevant aspects of the system(s) in which the decision(s) need(s) to be made.	P2L23 - P3L5
Study perspective	6	Describe the perspective of the study and relate this to the costs being evaluated.	P6L14, P6L29-30
Comparators	7	Describe the interventions or strategies being compared and state why they were chosen.	P5 L16-21
Time horizon	8	State the time horizon(s) over which costs and consequences are being evaluated and say why appropriate.	P6L4-12&21, P6L16
Discount rate	9	Report the choice of discount rate(s) used for costs and outcomes and say why appropriate.	P6L7, P7L17
Choice of health outcomes	10	Describe what outcomes were used as the measure(s) of benefit in the evaluation and their relevance for the type of analysis performed.	P6L10-14, P7L23-29
Measurement of effectiveness	11a	*Single study-based estimates:* Describe fully the design features of the single effectiveness study and why the single study was a sufficient source of clinical effectiveness data.	P4L14-16

For peer review only - http://bmjopen.bmj.com/site/about/guidelines.xhtml

	11b	*Synthesis-based estimates:* Describe fully the methods used for identification of included studies and synthesis of clinical effectiveness data.	P7L15-P8L14, ref 13
Measurement and valuation of preference based outcomes	12	If applicable, describe the population and methods used to elicit preferences for outcomes.	Na
Estimating resources and costs	13a	*Single study-based economic evaluation:* Describe approaches used to estimate resource use associated with the alternative interventions. Describe primary or secondary research methods for valuing each resource item in terms of its unit cost. Describe any adjustments made to approximate to opportunity costs.	P5L24-P6L8 TablsS1-5
	13b	*Model-based economic evaluation:* Describe approaches and data sources used to estimate resource use associated with model health states. Describe primary or secondary research methods for valuing each resource item in terms of its unit cost. Describe any adjustments made to approximate to opportunity costs.	P7L25, P8L7-8 Ref 13
Currency, price date, and conversion	14	Report the dates of the estimated resource quantities and unit costs. Describe methods for adjusting estimated unit costs to the year of reported costs if necessary. Describe methods for converting costs into a common currency base and the exchange rate.	P6L6-7, P7L16
Choice of model	15	Describe and give reasons for the specific type of decision-analytical model used. Providing a figure to show model structure is strongly recommended.	P7L15-6,FigS1, P8Sup1
Assumptions	16	Describe all structural or other assumptions underpinning the decision-analytical model.	SuplTS6(p10-11)
Analytical methods	17	Describe all analytical methods supporting the evaluation. This could include methods for dealing with skewed, missing, or censored data; extrapolation methods; methods for pooling data; approaches to validate or make adjustments (such as half cycle corrections) to a model; and methods for handling population heterogeneity and uncertainty.	P6L16-P7L12, P8L18-25
**Results**			
Study parameters	18	Report the values, ranges, references, and, if used, probability distributions for all parameters. Report reasons or sources for distributions used to represent uncertainty where appropriate. Providing a table to show the input values is strongly recommended.	P8L2-15, TblS1-S5
Incremental costs and outcomes	19	For each intervention, report mean values for the main categories of estimated costs and outcomes of interest, as well as mean differences between the comparator groups. If applicable, report incremental cost-effectiveness ratios.	Tabl1-3, P8L29-P10L2, P10L20-27
Characterising uncertainty	20a	*Single study-based economic evaluation:* Describe the effects of sampling uncertainty for the estimated incremental cost and incremental effectiveness parameters, together with the impact	P10L4-17, Fig1&2, TblS7

For peer review only

		of methodological assumptions (such as discount rate, study perspective).	Fig S2-4, P11L1-16
	20b	*Model-based economic evaluation:* Describe the effects on the results of uncertainty for all input parameters, and uncertainty related to the structure of the model and assumptions.	Na
Characterising heterogeneity	21	If applicable, report differences in costs, outcomes, or cost-effectiveness that can be explained by variations between subgroups of patients with different baseline characteristics or other observed variability in effects that are not reducible by more information.	P11L19-P12L15
**Discussion** Study findings, limitations, generalisability, and current knowledge	22	Summarise key study findings and describe how they support the conclusions reached. Discuss limitations and the generalisability of the findings and how the findings fit with current knowledge.	P15L1-7
**Other**			
Source of funding	23	Describe how the study was funded and the role of the funder in the identification, design, conduct, and reporting of the analysis. Describe other non-monetary sources of support.	P15L10-12
Conflicts of interest	24	Describe any potential for conflict of interest of study contributors in accordance with journal policy. In the absence of a journal policy, we recommend authors comply with International Committee of Medical Journal Editors recommendations.	

For consistency, the CHEERS Statement checklist format is based on the format of the CONSORT statement checklist

The **ISPOR CHEERS Task Force Report** provides examples and further discussion of the 24-item CHEERS Checklist and the CHEERS Statement. It may be accessed via the *Value in Health* link or via the ISPOR Health Economic Evaluation Publication Guidelines – CHEERS: Good Reporting Practices webpage: http://www.ispor.org/TaskForces/EconomicPubGuidelines.asp

The citation for the CHEERS Task Force Report is:
Husereau D, Drummond M, Petrou S, et al. Consolidated health economic evaluation reporting standards (CHEERS)—Explanation and elaboration: A report of the ISPOR health economic evaluations publication guidelines good reporting practices task force. Value Health 2013;16:231-50.

For peer review only

BMJ Open

The short-term and long-term cost-effectiveness of a pedometer-based exercise intervention in primary care: A within-trial analysis and beyond-trial modelling

Journal:	*BMJ Open*
Manuscript ID	bmjopen-2018-021978.R2
Article Type:	Research
Date Submitted by the Author:	17-Jul-2018
Complete List of Authors:	Anokye, Nana; Brunel University, Health Economics Research Group<br>Fox-Rushby, Julia; Kings College London, School Population Health and Environmental Sciences<br>Sanghera, Sabina ; University of Bristol, School of Social and Community Medicine<br>Cook, Derek; St George's University, Division of Population Health Sciences<br>Limb, Elizabeth; St George's University, Division of Population Health Sciences<br>Furness, Cheryl; St George's University, Division of Population Health Sciences<br>Kerry, Sally; Queen Mary University of London<br>Victor, Christina; Brunel University, Social Work & Community/Public Health<br>Iliffe, Steve; University College London, Primary Care and Population Sciences<br>Ussher, Michael; St George's University of London, Division of Population Health Sciences and Education<br>Whincup, Peter; St Georges, University of London<br>Ekelund, Ulf; Norwegian School of Sport Sciences<br>DeWilde, Stephen; St George's University of London<br>Harris, Tess; St George's University of London
**Primary Subject Heading**:	Health economics
Secondary Subject Heading:	Public health, General practice / Family practice, Sports and exercise medicine
Keywords:	physical activity, cost-effectiveness, RCT, long-term modelling

SCHOLARONE™
Manuscripts

Full Title: The short-term and long-term cost-effectiveness of a pedometer-based exercise intervention in primary care: A within-trial analysis and beyond-trial modelling

Short title: Economic evaluation of pedometers delivered through primary care

Nana Anokye[1] PhD, JuliaFox-Rushby[2] PhD, SabinaSanghera[3] PhD, Derek G. Cook[4] PhD, Elizabeth Limb MSc[4], Cheryl Furness MSc[4], Sally Kerry PhD[5], Christina Victor PhD[6], Steve Iliffe FRCGP[7], Michael Ussher PhD[4], Peter H.Whincup PhD[4], Ulf Ekelund PhD[8,9], Steve DeWilde MD[4], Tess Harris MD[4]

[1]Health Economics Research Group, Brunel University London, Kingston Lane, Uxbridge, UB8 3PH, UK;

[2]Department of Population Health Sciences, Guys Campus, Kings College London, SE1 1UL

[3]School of Social & Community Medicine, University of Bristol, BS8 1TH

[4]Population Health Research Institute, St George's University of London, Cranmer Terrace, London, SW17 0RE, UK;

[5]Pragmatic Clinical Trials Unit, Queen Mary's University of London, 4 Newark Street, London, E1 2AT, UK;

[6]Department of Clinical Sciences, Brunel University London, Kingston Lane, Uxbridge, UB8 3PH, UK;

[7]Research Department of Primary Care & Population Health, University College London, St Dunstans Road, London, W6 6RP, UK;

[8]Department of Sport Medicine, Norwegian School of Sport Sciences, Postbox 5 Torshov, 0412 Oslo, Norway;

[9]MRC Epidemiology Unit, University of Cambridge, Cambridge Biomedical Campus, Cambridge, CB2 0QQ, UK;

Corresponding author

Professor Julia Fox-Rushby, Department of Population Health Sciences, Faculty of Medicine and Life Sciences, 5th Floor Addison House, Guys Campus, Kings College London, SE1 1UL

Email: Julia.Fox-Rushby@kcl.ac.uk Telephone 020 7848 8672

Orcid id: 0000-0003-0748-0871

For peer review only - http://bmjopen.bmj.com/site/about/guidelines.xhtml

Abstract

Objectives: A short- and long-term cost-effectiveness analysis (CEA) of two pedometer-based walking interventions compared with usual care

Design: a) Short-term CEA: parallel three-arm cluster randomised trial randomised by household b) Long-term CEA: Markov decision-model

Setting: Seven primary care practices in South London, United Kingdom

Participants: a) Short-term CEA: 1023 people (922 households) aged 45-75yrs without physical activity (PA) contraindications b) Long-term CEA: 100,000 cohort aged 59-88yrs

Interventions: Pedometers, 12-wk walking programmes, and PA diaries delivered by post or through three PA consultations with practice nurses

Primary and Secondary Outcome Measures: Accelerometer-measured change (baseline-12months) in average daily step-count and time in 10-min bouts of moderate-vigorous PA, and EQ5D5L quality-adjusted life-years (QALYs)

Methods: Resource use costs (£2013/4) from an NHS perspective, presented as incremental cost-effectiveness ratios for each outcome over a 1-year and life-time horizon, with cost-effectiveness acceptability curves and willingness to pay per QALY. Deterministic and probabilistic sensitivity analyses evaluate uncertainty.

Results: a) Short-term CEA: At 12months, incremental cost was £3.61(£109) per minute in ≥10 minute MVPA bouts for nurse-support compared with control (postal group). At £20,000/QALY, the postal group had a 50% chance of being cost-saving compared with control. b) Long-term CEA: The postal group had more QALYs (+759QALYs, 95% CI 400, 1247) and lower costs (-£11m, 95% CI -12,-10), than control and nurse groups, resulting in an incremental net monetary benefit of £26m per 100,000 population. Results were sensitive to reporting serious adverse events, excluding health service use, and including all participant costs.

Conclusions: Postal delivery of a pedometer intervention in primary care is cost-effective long-term and has a 50% chance of being cost-effective, through resource savings, within one year. Further research should ascertain maintenance of the higher levels of PA, and its impact on quality of life and health service use.

Trial Registration: ISRCTN98538934

For peer review only - http://bmjopen.bmj.com/site/about/guidelines.xhtml

Strengths and Limitations of this study

- This study provides the first primary data on the short-term costs associated with delivering pedometers to a large (n=1023), population-based, sample from primary care alongside a high quality randomised controlled trial that achieved a 93% follow-up rate at 12 months.
- Results from the trial are fed into a peer-reviewed, policy-relevant, Markov model to estimate long-term cost-effectiveness as trials of public health interventions are unable to reflect the balance of costs and effects when benefits occur in the long term.
- Results are tested in a number of sensitivity analyses to assess the impact of changing perspective, missing data, changes assumptions about maintenance of PA and of taking more conservative views of outcomes and cost impact.
- The main limitation of the economic analysis is the lack of information about the likelihood of maintaining PA beyond three years into the long term and the exclusion of long term impacts on other conditions e.g. cancers

For peer review only

For peer review only - http://bmjopen.bmj.com/site/about/guidelines.xhtml

Introduction

Increasing physical activity (PA) is a widely-stated policy aim from local to international level.[1,2] Walking is a safe and, potentially cheap, activity that has the potential to reduce cardiovascular disease, diabetes, cancer and poor mental health.[3] It is therefore important to establish which approaches are effective at: encouraging inactive people to do at least some walking; increasing the number of people walking briskly for at least 150 mins a week (i.e. achieving moderate-to-vigorous PA (MVPA) guidelines[2]); and/or maintaining increases in walking over time. This would also provide the basis for estimating cost-effectiveness and supporting recommendations for policy and practice.

Until recently, the best evidence of pedometer-based walking programmes was from systematic reviews that relied on small, short-term, studies where the independence of pedometer effects, from other support provided was unclear.[4] These had shown that walking interventions can achieve increases of ~2000-2500 steps/day at 3 months, but often relied on volunteer samples or high risk groups and did not assess time in MVPA, as defined in PA guidelines, as an outcome. New evidence from a large, randomised, trial clustered by household (PACE-UP) compared delivery of pedometers by post or through primary care nurse-supported PA consultations. The trial was undertaken with 1,023 inactive primary care patients aged 45-75 years from seven practices in south London. Results showed that step-counts increased by around 10% and time in MVPA in 10-minute bouts by around a third, with both the nurse and postal delivery arms achieving similar 12-month outcomes.[4] This is important because primary care can be a key to reaching directly into the community and offering continuity of care for increasing PA. It is shown that this type of intervention is suitable for older adults, where exercise referral schemes have been disappointing[4]. Compared with national averages (from Health Survey for England 2012 dataset) for the same age range of the PACE-UP trial, the trial sample were more overweight/obese (66% vs 61%), more likely to have/have had a higher managerial, administrative, professional occupation (59% vs 36%), and less likely to be white (80% vs 93%)..

Other than a small, highly selected, study which limited outcomes to steps achieved among 79 people from one family physician practice in Glasgow,[5] there is no primary evidence of the cost-effectiveness of pedometer programmes in the UK. Elsewhere, in Australia, New Zealand, and the Netherlands, economic models from community-based adults with low PA levels compare pedometer prescriptions and pedometer-based telephone

For peer review only - http://bmjopen.bmj.com/site/about/guidelines.xhtml

coaching with usual practice.[6–8] These indicate, pedometer-based interventions may be cost-effective in the long term, but estimates vary widely and generalisability is not considered.[9]

The analytic horizon of cost-effectiveness analyses should extend far enough into the future to capture all benefits and harms, although in practice this can be limited by the amount and quality of data.[10] NICE's public health guidance[11] also recommends providing results that reflect the short term (one to three years). This is reinforced in NICE's return on investment models,[12] which argue that shorter-term decision-making is of key interest to some decision-makers and which have been used by commissioners.

This paper estimates the short-term (one year) and long-term (life-time) cost-effectiveness of pedometers delivered by post or through practice nurse consultation for 1,023 inactive adults aged 45-75years. The short-term evaluation arises from a within-trial analysis of individual resource use and costs of interventions provided in the PACE-UP trial.[4] The cost and effectiveness results from the trial are used to populate a long-term model[13] for life-time cost-effectiveness.

Methods

Short-term cost-effectiveness

The short-term within-trial cost-effectiveness analysis was conducted alongside the PACE-UP trial[4,14] that evaluated two intervention groups against control (no intervention group). The two intervention groups received pedometers (SW-200 Yamax Digi-Walker) (one by post), patient handbook; PA diary (including individual 12-wk walking plan), with the nurse group also offered three individually tailored practice nurse PA (10- to 20-min) consultations (nurse-support group only) at approximately weeks 1, 5, and 9.[4] The control group followed usual practice and were not provided with any feedback on their PA levels or materials promoting PA during the trial.[4] These interventions could therefore evaluate the incremental effect of adding nurse support to pedometers.

The costs for the two intervention arms include set-up costs, staff training and intervention delivery (including; pedometers & clips, batteries, handbooks, diaries, postage, nurse time, time making appointments). Measures of each resource use were taken from administrative/trial management records, computer-based diaries, and interviews with the trial manager and principal investigator. To account for potential changes in falls, change in

For peer review only - http://bmjopen.bmj.com/site/about/guidelines.xhtml

use of health services following differential contact of health services by participants or unintended resources consequences, general health service use (eg general (family) physician visits, hospital admissions, accident and emergency attendances, referrals) was collected at participant level, through a one-time download of physician records at the end of the trial, and linked to procedure codes using PI judgement (blind to treatment group) to facilitate costing across elective and non-elective admissions. Information on costs borne by patients (eg time use, out of pocket expenses associated with walking groups, plus any related travel costs) was collected by questionnaire at 3 and 12 months. Resources were valued using national tariffs where possible[15,16] to increase generalisability; where not available tariffs from St Georges Hospital, London, were used. All costs are expressed in £2013-2014 sterling, inflated to this base year where appropriate using the Hospital & Community Health Service inflation index. As the trial lasted for one year, a discount rate was not applied. (See Supplementary File Tables S1-S5). Physical activity was measured objectively by accelerometry (GT3X+. Actigraph LLC) and data were reduced using Actilife software (v 6.6.0). The summary variables used were as follows: step-counts; and time spent in MVPA in ≥10 minute bouts (≥1,952 Counts Per Minute, equivalent to ≥3 Metabolic Equivalents. [17]

Outcomes were; (a) changes in daily steps and weekly minutes of MVPA in bouts of ≥10 mins, and (b) changes in Quality Adjusted Life Years (QALYs), based on participant completion of the EQ-5D-5L questionnaires at baseline, 3 and 12 months. Utility weights were assigned using the 'crosswalk' function[18] linked to the standard UK-based weights[19], with QALYs based on the area under the curve.

Standard practice for accounting for missing data was followed. [20, 21] Patterns of missing data were investigated, with multiple imputation by chained equations fitted to replace item non-response. Missing EQ-5D data were replaced using an index rather than domain imputation as recommended[22]. Mean imputation was used where missing data was ≤5%[23]. Imputation models were fitted to match the model used for main analysis whilst including the predictors of missingness as appropriate. Second, the dependent variables were included in imputation models to ensure that the imputed values have similar relationships to the dependent variable as the observed values [24].

Results are reported, from an NHS perspective, as incremental cost-effectiveness ratios for cost per change in daily steps and cost per QALY for a one-year time-period, adjusted for baseline differences. A generalised

For peer review only - http://bmjopen.bmj.com/site/about/guidelines.xhtml

linear model was fitted separately for costs and QALYs with clustered standard errors. To provide more precise estimates of uncertainty, the 'margins method' was used to generate sample means by trial arm for costs and QALYs[24]. Cost models were fitted using the Poisson distribution and QALY models using the binomial 1 family, equivalent to beta regression[25]. The choice of distributional family for the models was based on the modified Park test and comparison of observed and predicted values. Covariates included baseline level (for the QALY-based models)[26], practice and variables found to be correlates of PA-related outcomes[27]- ie demography (age, gender, ethnicity, marital status, education, employment, socio economic status, cohabitation), health (number of disease conditions), and other lifestyle behaviours (smoking and alcohol intake). Reduced models were generated using Wald tests to examine the joint significance of variables found not to be significant (at 5%) in the base model.

Deterministic sensitivity analyses assessed: (a) inclusion of all randomised patients (rather than only those who provided accelerometry data); (b) exclusion of costs of general health service use beyond immediate intervention; (c) exclusion of missing data; (d) methods of accounting for adverse events; (e) perspective of analysis (ie including all and parts of participant costs); (f) varying the length of life of a pedometer; (g) the combination of excluding all health service use costs, and (h) including participant costs related to participation in physical activity and the interventions (minus health service use cost borne by participants, to ensure consistency in perspective). To reflect stochastic uncertainty surrounding mean incremental cost-effectiveness, cost-effectiveness planes (CEPs) and acceptability curves (CEACs) were constructed using 2000 non-parametric bootstrap samples from the base case estimates.

Long-term cost-effectiveness

A Markov model used to support NICE public health guidance[28] and return on investment modelling[12] was adapted to examine the long-term (life-time) cost effectiveness. From an NHS perspective, costs (2013/4 prices) and health outcomes from reduced disease, expressed as QALYs were discounted at the rate of 3.5% per annum. Results are reported as incremental cost-effectiveness ratios, cost-effectiveness acceptability curves and incremental net benefit statistics.

In the original model,[13] a cohort of 100,000 33 year-old people were followed in annual cycles over their life-time. At the end of the first year of the model, the cohort is either 'active' (doing 150 minutes of MVPA in 10

For peer review only - http://bmjopen.bmj.com/site/about/guidelines.xhtml

mins bouts per week) or 'inactive' and they could have one of 3 events (non-fatal CHD, non-fatal stroke, type 2 diabetes), remain event free (ie without CHD, stroke, or diabetes) or die either from CVD or non-CVD causes, each of which had assigned annual treatment costs (split by initial event and follow-up). After the first year, people would revert to PA patterns observed in long-term cohort studies (up to 10 year cycle in the model) on the relationship between PA and disease conditions[13]. The key driver of the long-term model is the protective effects of PA, which is a function of PA patterns after the first year of the intervention. In the base case analysis, PA behaviour was based on PA patterns observed in long-term cohort studies[29–31] on the relationship between PA and disease conditions. The cohort studies used followed up the same people (who were either active or inactive at baseline) for 10 years, during which some of the inactive people might have become active or vice versa. Thus the impact of changing habits is incorporated in the cohort relative risk (RR) estimates from these epidemiological studies. However, assuming that these estimates would persist after the follow-up periods might be impractical. It was therefore assumed, conservatively, that these RR estimates held for an initial 10-year period (i.e. the period PA patterns were observed in the epidemiological studies), after which no protective benefit would persist. Hence, the RRs for developing CHD, stroke and T2D in the first 10 years of the model were based on the estimates from the epidemiological studies but from year 11 onwards they were assumed to be equal to 1 (no effect). This assumption was tested sensitivity analyses.

Active individuals had lower risks of developing CHD, stroke and type-2 diabetes. People who become active in the first year (irrespective of trial arm) also accrue short-term psychological benefits, a one-off utility gain associated with achieving the recommended level of physical activity[13] (see supplementary file Figure S1).

The model was adapted, using data from the PACE-UP trial, in the following ways:

a) a cohort of 100,000 people aged 59 years followed, in annual cycles, to 88 years, reflecting the average age of all trial participants at baseline and the average life expectancy for people aged 59 years in UK[32] and exposed, at this age, to interventions (either nurse or postal) in an unexposed population ie control group/usual care;

(b) age-specific estimates were revised to reflect the change in the cohort age,

(c) within-trial cost of interventions was used, with a second year of annuitized values included appropriately - postal (£5·03/person) and nurse group (£4·14/ person);

(d) effectiveness was reflected as the relative risk of achieving ≥150 MVPA mins per week in ≥10 minute bouts; and

For peer review only - http://bmjopen.bmj.com/site/about/guidelines.xhtml

(e) short-term psychological benefits of PA (one-off utility gain) estimated using beta regression fitted for EQ-5D scores at 12 months for active people controlling for EQ-5D scores at baseline, demographics, practice, disability and trial arm using.

All other parameters remained the same as the original model, based on literature reviews or evidence from national/international science-based guidance on PA and health. Parameter estimates are provided in supplementary file Table S6.

Deterministic sensitivity analysis explored four, conservative, scenarios: (1) assuming the protective effects of PA exist only for 1 year, as the trial MVPA data was assessed at 12 months; (2) assuming the protective effects of PA exist for 3 years. Recent evidence[33] relating to 3 year follow-up of participants of the interventions showed persistent effect at 3 years; (3) Exclusion of all health service use cost consequences during trial period (model year one) and assumed no psychological benefits in the first year of being physically active. This was considered due to the uncertainty around short term changes to health service use and because previous studies found the exclusion of short-term QALY gain associated with being physically active to affect conclusions[13]; (4) Scenario 3 plus all patient costs related to participation in physical activity and the interventions (details of the participants costs are provided in supplementary file Table S4). Probabilistic sensitivity analysis was based on 10,000 Monte Carlo simulations and included all parameters except baseline mortality, as the mortality census data has little uncertainty.

Patient and Public Involvement

Patient and public involvement across the study is described in our publication of the main results,[4] and is reproduced below under the terms of the Creative Commons Attribution Licence (CC BY 4.0)

Pilot work with older primary care patients from three general practices was carried out ahead of seeking trial funding, with focus groups at each practice discussing ideas for a pedometer-based PA intervention. Patients were enthusiastic about the study and felt that the postal approach to recruitment and the interventions offered would be acceptable. They had input into aspects of the study design; for example, they encouraged us to offer the usual care arm a pedometer at the end of the follow-up period and they encouraged us to recruit couples as well as individuals, and to allow couples to attend nurse appointments together.

For peer review only - http://bmjopen.bmj.com/site/about/guidelines.xhtml

A patient advisor was a Trial Steering Committee member and was involved in discussions about recruitment and study conduct, as well as advising about patient materials, dissemination of results to participants, and safety reporting mechanisms.

All participants were provided with timely feedback of their individual trial results after completion of 12-mo follow-up, including their PA and body size measures over the trial duration. Summaries of results for the whole trial were disseminated to all trial participants as A4 feedback sheets after completion of baseline assessments and after analysis of the main results. A trial website (http://www.paceup.sgul.ac.uk/) has been created, and details have been circulated to participants. This also provides a summary of the trial results and details about further trial follow-up. All publications relating to the trial are provided on the website.

The burden of the intervention was assessed by all participants in the nurse group with a questionnaire as part of the process evaluation[34] and by samples of both intervention groups as part of the qualitative evaluation[35].

Results

Short-term cost-effectiveness

Table 1 summarises data on costs, EQ-5D-5L utility scores and QALYs by trial arm. At 3 months, average cost per participant was highest in the nurse group (£249) followed by the postal (£122) and control group (£107). In terms of the components of total costs, the cost of nurse-supported pedometer delivery was seven times greater (£50) than the postal group (£7), and set-up costs was double. Comparing the trial arms based on cost of health service use shows that the control group cost £35 more per participant than the postal group and £12 more than the nurse group. Results are similar at 12 months, except for the control arm, which has a higher overall average cost than the postal arm.

Table 2 shows that, at three months, mean incremental costs were significantly higher for the nurse group compared with the postal (+£120, 95% CI £95, £146) and control groups (+£135, 95% CI £99, £171) but not statistically significantly higher for the postal compared with control group. While increases in both daily steps and weekly minutes of MVPA in ≥10 minute bouts for both interventions compared with control, and for the nurse group compared with postal (nurse: +481steps (95% CI: 153, 809), +18mins MVPA (95% CI: 1, 35)) were statistically significant, the small mean decrease in QALYs is not statistically significant for any

For peer review only - http://bmjopen.bmj.com/site/about/guidelines.xhtml

comparison. The cost per additional minute of MVPA was 35p for postal group and £2·21 for the nurse group and therefore the (slightly) fewer QALYs for both interventions compared with control contributed to the dominance of each intervention by the control group (ie the control group cost less and had more QALYs). To move from a postal to nurse delivered pedometer would cost 25p per additional step and £6·67 per additional MVPA minute. However, in terms of cost-effectiveness, the nurse group costs more and produces less QALYs on average than the postal group at 3 months.

Results differ at 12 months. Compared with the control group, the postal arm cost less on average (-£91) and the nurse group more (+£126) but neither are statistically significant. The increase in cost of moving from a postal to nurse delivery is also statistically significantly higher (+£217, CI £81, £354). While both interventions are associated with a statistically significant increase in steps and weekly mins of MVPA, the difference between intervention groups is not statistically significant at 12 months. The small decrements in QALYs at each incremental comparison are not statistically different. The postal group took more steps (+642) and cost less on average (-£91) compared with control and dominates control in terms of PA outcomes. The nurse group cost 19p per additional step and £3.61 per additional minute of MVPA compared with control, with this rising to £6 and £109 respectively when compared with the postal group. In terms of QALYs, the nurse group is still dominated (ie cost more and had worse outcomes) by the control and postal groups. However, on average, each QALY lost in the postal group compared with control is associated with a saving of £21,162, which could therefore be considered cost-effective.

The probabilistic sensitivity analyses broadly confirm the findings of the base case; the postal group is most often associated with lower QALYs along with cost savings and the nurse group tends to have both lower QALYs and higher costs compared with control and postal group (Supplementary file, Figs S2-S4). Figure 1 shows that at £20,000 per QALY gained/lost, the postal group has a 50% chance of being cost-effective compared with control (usual care). This falls to 42% at £30,000/QALY, which reflects the postal group having most observations in the lower left-hand quadrant (as seen in Supplementary file, Fig S2). Figure 1 also shows that, at a willingness to pay/lose a QALY of £20,000, the nurse group has a 5.5% chance of being cost-effective compared with control.

For peer review only - http://bmjopen.bmj.com/site/about/guidelines.xhtml

The deterministic sensitivity analyses (Supplementary File, Table S7) mostly produced results consistent with the base case findings. However, in four circumstances, usual care would dominate both the postal and nurse groups at 12 months; i) using health service use based on self-reported serious adverse effects; ii) excluding all health service costs; iii) changing perspective (including all participant costs); and iv) the worst-case 'combined scenario' sensitivity analyses.

Long-term cost-effectiveness

Table 3 shows that, over the remaining life-time from age 59, the nurse group would be costlier (£11m, 95% CI: £10m, £12m) but have more QALYs (671 95% CI: 346, 1071) per 100,000 population than the control group and therefore provide each additional QALY at a cost of £16,368. However, the postal group would have lower life-time costs than the control arm (-£11m per 100,000 population, 95% CI: £-12m, £-10m) and more QALYs (759, CI: 400, 1247) it is therefore the dominant option, with an incremental net benefit of £26million per 100,000 population (95% CI: £18m, £36m). These results are confirmed by the incremental net benefit, which shows the £2m per 100,000 for nurse group compared with control is not significantly different and compared with the post group is significantly negative (-£24m 95% CI: -£27, -£21).

The stochastic uncertainty associated with the mean incremental cost-effectiveness ratio (ICER) (Figure 2) indicates the above findings are robust. There is a 100% likelihood, at a willingness to pay of £20,000/QALY, that the postal group is cost-effective compared with the control and nurse groups. This is consistent with the estimates of net monetary benefit in Table 3. At £20,000/QALY, there is a 70% likelihood that the nurse group would be cost-effective compared with control (Figure 2).

The results for the sensitivity analyses were:

(a) Scenario 1 - (i) postal vs control: postal remained dominant, less expensive (-£9m) with more QALY gains (+211QALYs); (ii) Nurse vs control: The ICER further increased from £16,000 to £69,000 (+£12.8m, +186QALYs); (iii) Nurse vs postal: The Nurse group remained dominated by postal group (+£21.6m, -32QALYs).

For peer review only - http://bmjopen.bmj.com/site/about/guidelines.xhtml

(b) Scenario 2 - (i) postal vs control: postal was still dominant, less expensive (-£9.2m) with more QALY gains (+327QALYs); (ii) Nurse vs control: The ICER increased from £16,000 to £43,000 (+£12.4m, +289QALYs); (iii) Nurse vs postal: The Nurse group remained dominated by postal group (+£21.7m, -48QALYs).

(c) Scenario 3 - (i) postal vs control: postal moved from a dominant position to a more expensive option (+£4m) with more QALY gains (+609QALYs), and an ICER of £6,100; (ii) Nurse vs control: The ICER increased from £16,000 to £26,000 (+£14m, +538QALYs); (iii) Nurse vs postal: The Nurse group remained dominated by postal group (+£10m, -87QALYs).

(d) Scenario 4 - (i) postal vs control: postal moved from a dominant position to more expensive (+£16m) and more QALY gains (+609 QALYs) with an ICER of £26,600; (ii) Nurse vs control: The ICER increased from £16,000 to £25,400 (+£13.7m, +538QALYs); (iii) Nurse vs postal: Nurse moved from dominated position (where costs are higher and QALYs lower to a cost-effective position (where both costs and QALYs are lower) (-£2m, -87QALYs).

Discussion

The life-time cost-effectiveness of posting a pedometer with written instructions to a cohort of 100,000 insufficiently active people aged 59 years (who have indicated an interest in research or participation in walking) would cost less (-£11m, 95%CI -12,-10) and provide more QALYs (759 QALYs, 95%CI 400, 1247) than usual care. Most cost-savings and quality of life benefits derive from reductions in stroke, CHD and type-2 diabetes. This finding was robust (incremental net benefit of £26m, 95%CI £18m, £36m) and sensitivity analyses showed that even excluding short-term cost savings would not change the conclusion that the postal group would be extremely cost-effective in the long-term (ICER: £6,100/QALY). Sending a pedometer by post with instructions from a primary care provider to inactive people aged 45-75 also has a 50% chance of being cost-effective *within a year*, as a 1 QALY loss was associated with saving over £21,000. The nurse group had higher costs and lower QALYs than both control and postal groups at 1 year. While sensitivity analyses did not change conclusions in most cases, in three cases (using self-reported serious adverse events, excluding health service use, including all participant costs) it did, indicating that the control group would dominate (ie have lower costs and more QALYs) than both the postal and nurse groups.

For peer review only - http://bmjopen.bmj.com/site/about/guidelines.xhtml

A key strength of this study is the base of individualised cost and effectiveness data on a large, population-based, cluster-randomised, controlled trial with excellent follow-up data to one year (93.4%, Harris et al 2017)[4], designed to produce generalisable results, for cost per QALY estimates at one year and as inputs to a long-term model of cost-effectiveness. It is also the only study to have included provider and user perspectives, extended commonly used techniques to account for clustering and used conservative assumptions for both short- and long-term sensitivity analyses.

One weakness of the within-trial cost-effectiveness study concerns the use of PI judgement to determine costs of admissions, and therefore alternative assumptions were explored in sensitivity analyses. Patient reported cost data were collected for months 1-3 and 9-12, with the last 3 months multiplied to represent costs across all months from 4-12. If significantly underestimated, this could be decisional. To date, there are no primary economic data beyond 12 months of an intervention and very few trials include measures of quality of life measures alongside PA. Therefore, with respect to the long-term modelling, a key gap in knowledge is the likelihood of maintaining PA beyond 12 months. This model assumes differences in PA at 1 year in the trial relate to the same long-term benefit associated with the same difference in cohort studies, but this could be updated once longer-term follow-up data become available. Other challenges set out in Anokye et al 2014[13] are relevant here eg cancer and adverse events are not accounted for, which could lead to over or under-estimation of cost-effectiveness. Other challenges relate to the generalisability of effectiveness data, given the focus on South London and 10% recruitment rate, even though recruitment was comparable with other PA trials [36,37]. The trial was shown to recruit fewer: men, people aged 55-64yrs compared with those over 65yrs, people from the most deprived quintile compared with least deprived, and Asian compared with white people[37,]. However, there was good representation of women, older adults and people who were overweight, all of whom are groups likely to benefit from the intervention[4]. Investigation into the reasons for non-participation showed an important minority cited existing medical conditions, too many other commitments or considered themselves sufficiently active[35,38].

.

This study feeds into an area with very limited primary data[39,40] populated only by small studies[5,6]. In New Zealand, pedometers were shown to have a 95% probability of being a cost-effective addition to green prescriptions at 12 months[5], much higher than the 50% likelihood we found. Other models of long-term cost-

For peer review only - http://bmjopen.bmj.com/site/about/guidelines.xhtml

effectiveness studies identified cost savings and improved quality of life at a population level from pedometers in the long term[8,41] or indicated high probabilities of long-term cost[7,42]. Guidance has also suggested that long-term monitoring/support at £25/year would be very cost-effective. Our study provides further support that pedometer-based programmes are a cost-effective method of improving health-related quality of life in both the short and long-term. Assumptions about intervention effectiveness beyond one year has mixed impacts, and further research is required to better judge whether existing models over- or under-predict cost-effectiveness.

Current public health guidance from NICE on pedometers[43] advises using pedometers as "part of a package which includes support to set realistic goals in one to one meetings (whereby the number of steps taken is gradually increased), monitoring and feedback. Our results not only provide substantially better economic data for use by NICE but also suggest guidance should be updated to reflect the value of providing pedometers, to people who have made some form of commitment (ie to a trial), through the post. For those practices that have implemented consultation-based distribution of pedometers, moving to postal delivery could save costs within a year, with similar outcomes.

Postal delivery of pedometer interventions to inactive people aged 45-75 through primary care is cost-effective in the long-term and has a 50% chance of being cost-effective, through resource savings, within one year. Further research is needed to ascertain the extent to which higher PA levels are maintained beyond three years and the impact of PA on quality of life and general health service use in both the short and long-term.

For peer review only - http://bmjopen.bmj.com/site/about/guidelines.xhtml

Supporting Information

Supplementary file 1: Copy of open access protocol for trial

Supplementary file 2: Figures 1 & 2

Supplementary file 3: Document for online supplement giving fuller study details

Supplementary file 4: CHEERS reporting statement

Author Contributions

Contributors: JFR conceived of the economic analysis, was co-applicant for funding, jointly designed the economic data collections tools, wrote the economic analysis plan, collected part of the data, supervised the economics, and jointly drafted and amended the script. She is guarantor for this script. NA jointly designed the data collection tools, cleaned and analysed the economic data, and jointly drafted and amended the script. SS collated and analysed the hospital cost data, commented on drafts and reviewed the final script. DC, EL and SK designed data collection for and analysed the intermediate outcome data underpinning the economic analyses, discussed plans and results as presented through the trial, commented on drafts of this manuscript and reviewed the final script. CF collated and provided access to the administrative data used for the economic analysis, was the research project administrator, commented on drafts and reviewed the final script. TH was the principal investigator, involved at all points of the planning, progress and review of the economic evaluation including commenting on drafts and review of the final script. She is guarantor for the whole trial. CRV, PHW, MU, SI, UE,SdW all conceived the trial plan and applied for funding, they contributed to conceptualisation of the economics within the broader context of the trial, discussed plans and results as presented through the trial, commented on drafts of this manuscript and revised the final script.

Acknowledgements

We thank Iain Carey for help with processing the primary care data as well as Abbie Hill and Julie Whittaker who ably supported administration of the economics project at Brunel University London. We remember and

For peer review only

thank too, our colleague and co-investigator, Dr S M Shah who died in September, 2015. We would like to thank the South-West London (UK) general practices, their practice nurses who supported this study, and all the patients from these practices who participated: Upper Tooting Road Practice, Tooting; Chatfield Practice, Battersea; Wrythe Green Practice, Carshalton; Francis Grove Practice, Wimbledon; Putneymead Practice, Putney; Heathfield Practice, Putney; and Cricket Green Practice, Mitcham. We would also like to thank our supportive Trial Steering Committee: Professor Sarah Lewis (chair); Professor Paul Little (GP representative); Mr Bob Laventure (Patient and Public Involvement representative).

For peer review only

Funding Statement

This research was supported by the Health Technology Assessment (HTA) Programme, National Institute for Health Research (project number HTA 10/32/02 ISRCTN42122561), The funding organisation played no role in the study design, collection, analysis and interpretation of the data, writing or decision to submit the paper for publication The views and opinions expressed therein are those of the authors and do not necessarily reflect those of the Health Technology Assessment (HTA) Programme, National Institute for Health Research, National Health Service or the Department of Health.

Competing Interests

JFR reports Board Membership of the Public Health Research Funding Board of NIHR. TH sits on Primary Care Community Interventions Panel for the Health Technology Assessment Programme of NIHR. All authors have been contracted to evaluate other public health interventions, including pedometers, for the NIHR.

Patient Consent

No individual can be identified from the work presented in this paper.

Data Sharing

Data is available upon request from Dr Tess Harris

For peer review only

References

1. Lichfield District Council. (Physical Activity and Sport Strategy 2016-2020. 2016.: Available from https://www.lichfielddc.gov.uk/Residents/Leisure-and-culture/Leisure-centres/Downloads/Lichfield-District-Physical-Activity-and-Sport-Strategy.pdf. (downloaded 21.02.201)

2. World Health Organisation. Global recommendations on physical activity for health 2010.http://www.who.int/dietphysicalactivity/publications/9789241599979/en/

3. Kyu H, Bachman V, Alexander L, Mumford J, Afshin A, Estep K, et al. Physical activity and risk of breast cancer, colon cancer, diabetes, ischemic heart disease, and ischemic stroke events: systematic review and dose-response meta-analysis for the Global Burden of Disease Study 2013. Br Med J. 2016;354:i3857

4. Harris T, Kerry SM, Limb ES, Victor CR, Iliffe S, Ussher M et al. Effect of a Primary Care Walking Intervention with and without Nurse Support on Physical Activity Levels in 45- to 75-Year-Olds:The Pedometer And Consultation Evaluation (PACE-UP) Cluster Randomised Clinical Trial. Plos Med. 2017;14;1 e1002210

5. Shaw R, Fenwick E, Baker G, McAdam C, Fitzsimons C, Mutrie N. Pedometers cost buttons: the feasibility of implementing a pedometer based walking programme within the community. BMC Public Health. 2011; 11:200 http://www.biomedcentral.com/1471-2458/11/200

6. Leung W, Ashton T, Kolt G, Schofield G, Garrett N, Kerse N, et al.Cost-effectiveness of pedometer-based versus time-based Green Prescriptions: the Healthy Steps Study. Aust J Prim Hlth. 2012;18:204–211

7. Over E, Wendel-Vos G, van den Berg M, Hamberg-van Reenen H, Tariq L, Hoogenveen R, et al. Cost-effectiveness of counseling and pedometer use to increase physical activity in the Netherlands: a modeling study. Cost Eff Res Alloc. 2012; 10:13

8. Cobiac LJ, Vos T, Barendregt JJ. Cost-effectiveness of interventions to promote physical activity: a modelling study. PLOS medicine. 2009;6;7 https://doi.org/10.1371/journal.pmed.1000110

9. Vijay G, Wilson E, Suhrcke M, Hardeman W, Sutton S, on behalf of the VBI Programme Team . Are brief interventions to increase physical activity cost-effective? A systematic review. Br J Sports Med. 2016;50:408-417

10. Honeycutt A, Clayton L, Khavjou O, Finkelstein E, Prabhu M, Blitstein J et al. Guide to Analyzing the Cost-Effectiveness of Community Public Health Prevention Approaches Report to U.S. Department of Health and Human Services RTI Project Number 0208827.001. 2006. Available from: https://aspe.hhs.gov/system/files/pdf/74686/report.pdf

11. National Institute for Health and Care Excellence. Developing NICE guidelines: the manual. 2014 Available from: https://www.nice.org.uk/process/pmg20/chapter/incorporating-economic-evaluation

12. Matrix. Estimating Return on Investment for interventions and strategies to increase physical activity. London: Matrix Knowledge, 2014. Available from https://www.nice.org.uk/Media/Default/About/what-we-do/Into-practice/Return-on-Investment/NICE-return-on-investment-physical-activity-technical-report.pdf

13. Anokye, NK., Lord, J., Fox-Rushby, J., Is brief advice in primary care a cost-effective way to promote physical activity? Br J Sp Med. 2014;48:(3:202–206

14. Harris T, Kerry SM, Victor CR, Shah SM, Iliffe S, Ussher M, Ekelund U et al. PACE-UP (Pedometer and consultation evaluation - UP) - a pedometer-based walking intervention with and without practice nurse support in primary care patients aged 45-75 years: study protocol for a randomised controlled trial. Trials. 2013;14:418.

For peer review only - http://bmjopen.bmj.com/site/about/guidelines.xhtml

15. Department of Health . NHS Reference Costs 2013 to 2014. 2015 Available from:https://www.gov.uk/government/publications/nhs-reference-costs-2013-to-2014

16. Curtis LA. Unit costs for health and social care. Personal Social Services Research Unit, University of Kent: Canterbury; 2014 Available from https://www.pssru.ac.uk/project-pages/unit-costs/unit-costs-2014/

17. Freedson PS, Melanson E, Sirard J. Calibration of the computer science and applications, Inc. accelerometer. Med Sci Sp Exe. 1998;30:777-781

18. EuroQol Group. EQ-5D-5L Use Guide: Basic Information on how to use the EQ-5D-5L Instrument Version 2.1. 2015. Available from https://euroqol.org/wp-content/uploads/2016/09/EQ-5D-5L_UserGuide_2015.pdf.

19. Dolan P: Modeling valuations for EuroQol health states. Med Care. 1997:35;11:1095-108

20. Allison PD. Multiple Imputation for Missing Data: A Cautionary Tale. Soc Meth Res. 2000;28:301-9.

21. Carpenter JR, Kenward, M.G. MAR methods for quantitative data. In: Missing data in randomised controlled trials— a practical guide. National Institute for Health Research. 2008.

22. Simons CL, Rivero-Arias O, Yu LM, Simon J. Multiple imputation to deal with missing EQ-5D-3L data: Should we impute individual domains or the actual index? Qual Life Res. 2014:24:805=815

23. Schafer, J. L. Multiple imputation: A primer. Stat Meth Med Res. 2009; 8:3–15.

24. Sterne JA, White IR, Carlin JB, Spratt M, Royston P, Kenward MG, et al. Multiple imputation for missing data in epidemiological and clinical research: potential and pitfalls. Br Med J. 2009;338:b2393.

25. Glick HA, Doshi JA, Sonnad SS, Polsky D. Economic evaluation in clinical trials. (2 ed.) Oxford University Press 2014

26. Basu A, Manca A. Regression estimators for generic health-related quality of life and quality adjusted life years. Med Decision Making. 2012; 32: 56–69

27. Smith L, Gardner B, Fisher A, Hamer M. (2015) Patterns and correlates of physical activity behaviour over 10 years in older adults: prospective analyses from the English Longitudinal Study of Ageing. BMJ Open. 2015;5:e007423. doi:10.1136/bmjopen-2014- 007423

28. National Institute for Health and Care Excellence. Public Health Guidance [PH44] Physical activity: brief advice for adults in primary care. 2013 https://www.nice.org.uk/guidance/PH44 (Accessed June 2018)
.

29. Hu G, Qiao Q, Silventoinen K, Eriksson JG, Jousilahti P, Lindstrom J et al, Occupational, commuting, and leisure-time physical activity in relation to risk for Type 2 diabetes in middle-aged Finnish men and women. Diabetologia. 2003;46:322–329

30. Hu G, Sarti C, Jousilahti P, Silventoinen K, Barengo NC, Jaakko Tuomilehto Leisure Time, Occupational, and Commuting Physical Activity and the Risk of Stroke. Stroke. 2005;36:1994-1999.

31. Hu G, Jousilahti P, Borodulin K, Barengo NC, Lakka TA, Nissinen A et al. Occupational, commuting and leisure-time physical activity in relation to coronary heart disease among middle-aged Finnish men and women..Atherosclerosis. 2007;194:490-7

32. Office for National Statistics . Life Expectancy. ONS Digital 2015 (Accessed August 2015) Available from: http://visual.ons.gov.uk/how-long-will-my-pension-need-to-last/)

For peer review only - http://bmjopen.bmj.com/site/about/guidelines.xhtml

33. Harris T, Kerry SM, Limb ES, Furness C, Wahlich C, Victor C et al. Physical activity levels in adults and older adults 3-4 years after pedometer-based walking interventions: Long-term follow-up of participants from two randomised controlled trials in UK primary care. Plos Med. 2018;15:3;e1002526 https://doi.org/10.1371/journal.pmed.1002526

34. Furness C, Howard E, Limb E, Cook D, Kerry S, Wahlich C, et al. Relating process evaluation measures to complex intervention outcomes: findings from the PACE-UP primary care pedometer-based walking trial. *Trials* 2018;19:58

35. Normansell R, Holmes R, Victor C, Cook DG, Kerry S, Iliffe S, et al. (2016) Exploring non-participation in primary care physical activity interventions: PACE-UP trial interview findings. Trials. 17:178 doi: 10.1186/s13063-016-1299-z

36. Tully MA, Cupples ME, Chan WS, McGlade K, Young IS. Brisk walking, fitness, and cardiovascular risk: a randomized controlled trial in primary care. Prev Med. 2005;41;2:622-8.

37. McMurdo ME, Sugden J, Argo I, Boyle P, Johnston DW, Sniehotta FF, et al. Do pedometers increase physical activity in sedentary older women? A randomized controlled trial. J Am Geriatr Soc. 2010;58;11:2099-106

38. Kerry S, Morgan K, Limb E, Cook D, Furness C, Carey I, et al.. (2018) Interpreting population reach of a large, successful physical activity trial delivered through primary care. BMC Pub Hlth. 18:170 doi:10.1186/s12889-018-5034-4

39. Laine J, Kuvaja-Kollner V, Pietila E, Koivuneva M, Valtonen H, Kankaanpdd E. Cost-Effectiveness of Population-Level Physical Activity Interventions: A Systematic Review. Am J Hlth Prom. 2014: 29;2;71–80

40. Garrett S, Raina Elley C, Rose SB. Are physical activity interventions in primary care and the community cost-effective? A systematic review of the evidence. Br J Gen Prac. 2011;61;584:e125-e133

41. de Smedt D, de Cocker K, Annemans L, de Bourdeaudhuij I, Cardon G. A cost-effectiveness study of the community-based intervention '10000 Steps Ghent'. Pub Hlth Nut.. 2012;15:3:442–51

42. Brennan A, Blake L, Hill-McManus D, Payne N, Buckley Woods H, Blank L. Walking and cycling: local measures to promote walking and cycling as forms of travel or recreation: Health economic and modelling report. National Institute for Health and Clinical Excellence Public Health Programme. 2012. Available from: https://www.nice.org.uk/guidance/ph41/evidence/economic-modelling-report-430262893

43. National Institute for Health and Care Excellence, Public Health Guidance [PH41] Physical activity: walking and cycling. 2012. National Institute for Health and Clinical Excellence Public Health Programme. Available from: https://www.nice.org.uk/guidance/ph41/resources/physical-activity-walking-and-cycling-pdf-1996352901061

For peer review only - http://bmjopen.bmj.com/site/about/guidelines.xhtml

Table 1: Average costs and QALYs per participant, by trial arm, (£'sterling2013/14, all randomised participants who provided required accelerometry data*, missing data imputed)

**Cost and quality of life (EQ5D5L)**	**Control**	**Postal**	**Nurse**
	**Mean (SD)**		
**0-3 months**	**n=318**	**n=317**	**n=319**
Total cost	*£107 (254)*	*£122(107)*	*£249 (215)*
Set up	£0 (0)	£45(0)	£105(0)
Delivery of intervention	£0 (0)	£7 (0)	£50 (18)
Health service use	£107(254)	£71(107)	£95 (214)
EQ 5D scores at baseline	0.839 (0.14)	0.853 (0.12)	0.851 (0.12)
EQ 5D scores at 3 months	0.844 (0.14)	0.848 (0.14)	0.841 (0.14)
QALYs 0-3 months	0.194 (0.03)	0.196 (0.03)	0.195 (0.03)
**0-12 months**	**n=323**	**n=312**	**n=321**
Total cost	*£461 (916)*	*£375(611)*	*£603 (987)*
Set up	£0 (0)	£45 (0)	£105 (0)
Delivery of intervention	£0 (0)	£10 (0)	£52 (18)
Health service use	£461 (916)	£320 (611)	£447 (987)
EQ 5D scores at baseline	0.837 (0.14)	0.850 (0.12)	0.849 (0.13)
EQ 5D scores at 3 months	0.840 (0.14)	0.847 (0.13)	0.837 (0.14)
EQ 5D scores at 12 months	0.833 (0.15)	0.836 (0.13)	0.831 (0.14)
QALYs 0-12 months	0.837 (0.13)	0.843 (0.11)	0.836 (0.13)

*The number of people who provided accelerometry data differed across time points within arms * For incremental analyses, the comparisons are postal vs control and nurse vs control

For peer review only - http://bmjopen.bmj.com/site/about/guidelines.xhtml

Table 2: Regression estimates for costs, effects and cost-effectiveness at 3 and 12 months(£'sterling 2013/14) (base case, adjusted for baseline differences)

Cost, effects or cost-effectiveness		Control		Postal*		Nurse*		Nurse vs Postal	
		Mean	(95% CI)	Mean	(95% CI)	Mean	(95% CI)	Mean	(95% CI)
Costs and effects over 3 months	Total cost per participant (£)	108	(80 to 136)	123	(111 to135)	244	(221 to 266)	-	
	Incremental cost (£)	-		15	(-15 to 45)	135	(99 to 171)	120	(95 to 146)
	Total QALYs per participant	0.1957	(0.1936 to 0.1978)	0.1952	(0.1930 to 0.1974)	0.1948	(0.1926 to 0.1970)	-	
	Incremental* QALYs	-		-0.0005	(-0.0027 to 0.0016)	-0.0009	(-0.0031 to 0.0012)	-0.0004	(-0.0026 to 0.0018)
	Incremental daily steps			692	(363 to 1020)	1172	(844 to 1501)	481	(153 to 809)
	Incremental weekly mins of MVPA in bouts of ≥10 mins			43	(26 to 60)	61	(44 to 78)	18	(1 to 35)
Costs and effects over 12 months	Total cost per participant (£)	467	(365 to 569)	376	(307 to 445)	593	(473 to 714)	-	
	Incremental cost (£)	-		-91	(-215 to 33)	126	(-37 to 290)	217	(81 to 354)
	Total QALYs per participant	0.842	(0.832 to 0.853)	0.838	(0.827 to 0.849)	0.836	(0.824 to 0.847)	-	
	Incremental QALYs	-		-0.004	(-0.017 to 0.009)	-0.007	(-0.020 to 0.007)	-0.002	(-0.016 to 0.011)
	Incremental daily steps	-		642	(329 to 955)	677	(365 to 989)	36	(-227 to 349)
	Incremental weekly mins of MVPA in bouts of ≥10 mins	-		33	(17 to 49)	35	(19 to 51)	2	(-14 to 17)
IC[illegible]	Cost per additional QALY (£)	-		*Postal dominated by control*		*Nurse dominated by control*		*Nurse dominated by Postal*	

For peer review only - http://bmjopen.bmj.com/site/about/guidelines.xhtml

Cost, effects or cost-effectiveness		Control		Postal*		Nurse*		Nurse vs Postal	
		**Mean**	**(95% CI)**	**Mean**	**(95% CI)**	**Mean**	**(95% CI)**	**Mean**	**(95% CI)**
	Cost per additional step count (£)	-		£0.02		£0.12		£0.25	
	Cost per additional minute of MVPA in a bout of ≥10 mins (£)	£0.35		£0.35		£2.21		£6.67	
ICER* at 12 months	Cost per additional QALY (£)	-		*Postal is less costly but has fewer QALYs. £21,162 saved per QALY lost*		*Nurse dominated by control*		*Nurse dominated by Postal*	
	Cost per additional step count (£)	-		*Postal dominates control*		0.19		6.03	
	Cost per additional minute of MVPA in a bout of ≥10 mins (£)	-		*Postal dominates control*		3.61		109.00	

For peer review only

Table 3: Costs, effects and cost-effectiveness over a lifetime from age 59 (100,000 cohort)

	**Control**	**Postal***	**Nurse ***	**Nurse vs Postal**
	**Mean (95% CI)**	**Mean (95% CI)**	**Mean (95% CI)**	**Mean (95% CI)**
Lifetime total cost (£million) **	340 (307, 371)	329 (296, 361)	351 (318, 384)	-
Lifetime incremental cost (£million)	-	-11 (-12, -10)	11 (10, 12)	22 (21 to 23)
Lifetime total QALYs (million)	1.0709 (0.879, 1.273)	1.0717 (0.889, 1.274)	1.0716 (0.880, 1.273)	-
Lifetime incremental QALYs	-	759 (400, 1247)	671 (346, 1071)	-108 (-223 to -10)
Lifetime ICER for QALYs (£)		Postal dominates control	16,368	Postal dominates nurse
Lifetime Incremental Net Monetary Benefit (£million, @ £20,000 per QALY)	-	26 (18, 36)	2 (-5, 11)	-24 (-27 to -21)

* For incremental analyses, the comparisons are postal vs control and nurse vs control.

**£46.7m, £37.6m and £59.3m of the total costs for control, postal and nurse groups respectively, were estimated using PACE-UP trial results

For peer review only - http://bmjopen.bmj.com/site/about/guidelines.xhtml

Figure Legends

Figure 1: Cost-effectiveness acceptability curve showing the probability of short-term (at 1 year) cost-effectiveness for postal and nurse groups (vs control) at different willingness to pay per QALY thresholds.

Figure 2: Cost-effectiveness acceptability curve showing the probability of life-time cost-effectiveness for postal and nurse groups (vs control) at different willingness to pay per QALY threshold levels

For peer review only

For peer review only - http://bmjopen.bmj.com/site/about/guidelines.xhtml

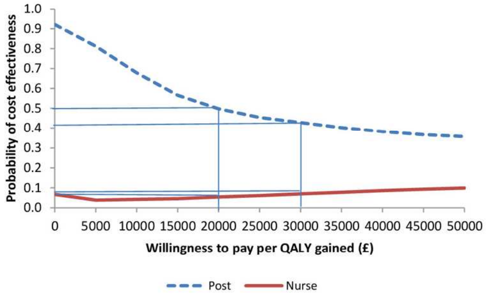

Figure 1: Cost-effectiveness acceptability curve showing the probability of short-term (at 1 year) cost-effectiveness for postal and nurse groups (vs control) at different willingness to pay per QALY thresholds.

140x198mm (300 x 300 DPI)

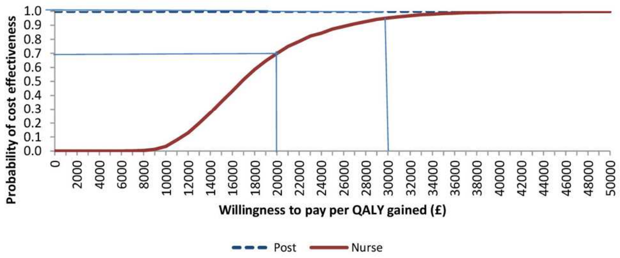

Figure 2: Cost-effectiveness acceptability curve showing the probability of life-time cost-effectiveness for postal and nurse groups (vs control) at different willingness to pay per QALY threshold levels

140x198mm (300 x 300 DPI)

Supplementary file for

"The short-term and long-term cost-effectiveness of a pedometer-based intervention in primary care: A within-trial analysis and beyond-trial modelling"

Nana Anokye PhD, Julia Fox-Rushby PhD, Sabina Sanghera PhD, Derek G. Cook PhD, Elizabeth Limb MSc, Cheryl Furness MSc, Sally Kerry PhD, Christina Victor PhD, Steve Iliffe FRCGP, Michael Ussher PhD, Peter H.Whincup PhD, Ulf Ekelund PhD, Steve DeWilde PhD, Tess Harris MD

For peer review only

Table S1: Resource use and cost components of 'Set-up Cost'*

**Activity (trial arm applicable to)**	**Resource**	**Total quantity**	**Cost per participant £ (nurse group)**	**Cost per participant £ (post group)**
**Design^**				
Designing of intervention (Both intervention arms)	Professor x1	0·5 days	4·43	4·43
	Readers x3	1 day		
	Senior lecturers x3	3·5 days		
	Consultants x2	1 days		
Designing of participants' handbooks and diaries (both intervention groups)	Professor x3	1·5 days	3·56	3·56
	Readers x2	1 day		
	Senior lecturers x3	2 days		
	Consultants x2	0·5 days		
Designing of nurse trainers handbooks (Nurse group)	Senior lecturers x1	1 day	2·74	0
	Consultants x1	0·5 days		
	Handbooks	9 handbooks	0·19	0
**Setting up GP practices**				
Planning for recruitment of practices (All trial arms)	Professor x1	1 hour	0·99	0·99
	Senior lecturer x1	5 hours		
	Consultants x2	5 hours		
Visits to recruit 6 practices (All trial arms)	Senior lecturers x2	13 hours	1·47	1·47
	Trial Manager x1	7 hours		
	Consultant x1	5 hours		
	Round trips to practices (by all)	25 hours	0·10	0·10
Searching practice computers to identify participants (All trial arms)	Senior lecturer x1	6 hours	0·71	0·71
	Trial Manager x1	6 hours		
	Practice Manager x6	6 hours		
Identify households from anonymised address list (All trial arms)	Senior lecturer x1	32 hours	2·28	2·28
	Trial Manager x1	32 hours		
Practice staff reviews lists for exclusion (All trial arms)	GP x5 (for sorting out 2 practices)	20 hours	4·50	4·0

Activity (trial arm applicable to)	Resource	Total quantity	Cost per participant £ (nurse group)	Cost per participant £ (post group)
	Nurse x10 (for sorting out other 5 practices)	50 hours	1·96	1·96
Printing letters at practice (All trial arms)	Trial Manager x1	64 hours	1·57	1·57
	Practice administrative staff x2	4 hours		
	Number of printed letters	24000	0·94	0·94
Packing envelopes with leaflets and letters (All trial arms)	Trial Manager x1	240 hours	7·04	7·04
	Research Assistants x2	56 hours		
	Practice admin. Staff x11	27·5 hours		
	Cost of Envelopes	£497·30	0·49	0·49
	Cost of Postal stamps	£5,530·50	5·41	5·41
	Cost of Information leaflets	£5,973·00	5·84	5·84
Preparing rooms at practices for trial (All trial arms)	Round trip to practices by RA	14 trips	0·04	0·04
	Research Assistants x2	-*	0·11	0·11
**Training**				
Training of Trial manager (All trial arms)	Trial Manager x1	4 days	1·51	1·51
	Senior lecturer x1	2 days		
Preparation of nurse training course (Nurse support group)	Trial Manager x1	1 day	9·63	0
	Senior lecturer x1	2 days		
	Reader x1	0·5 days		
	Consultants x2	2 days		
Mini-training day of nurses (Nurse group)	Nurses x11	33 hours	7·46	0
	Trial Manager x1	17·33 hours		
	Senior lecturer x1	17·33 hours		
	Round trips to training centre (by tutors)	16 hours	0·19	0
	Pedometers given to nurses	12 hours	0·04	0
Full training day of nurses (Nurse group)	Nurses x10	107·5 hours	22·99	0
	Reader x1	1 hour		
	Senior lecturer x1	10 hours		
	Consultants x2	22·5 hours		
	Round trips for training by nurses x10	10 trips	0·12	0
	Round trips for training by consultants x2	2 trips	0·13	0
	Refreshments	1 set	0·26	0
Training for an absentee nurse (Nurse group)	Nurse x 1	10 hours	2·47	0
	Trial Manager x1	11·33 hours		
	Research assistant x1	11·33 hours		
	Round trips to training centre	2 trips	0·02	0
Discussion of nurses recorded sessions(Nurse group)	Senior lecturer x1	0·5 days	3·78	0
	Consultants x2	1 day		
	Nurses x9	4·5	0·99	0
	Senior lecturer x1	0·5		

For peer review only

Activity (trial arm applicable to)	Resource	Total quantity	Cost per participant £ (nurse group)	Cost per participant £ (post group)
	Consultants x2	1		
	Duration of phone calls	270 mins	0·09	0
Follow-up half day training(Nurse group)	Nurses x 9	4.5 days	7·70	0
	Trial Manager x1	0·5 days		
	Senior lecturer x1	0·5 days		
	Consultants x2	1 day		
	Nurse time travelling x 9	6·75 hours	0·78	0
	Round trips to training centre (nurses)	9 trips	0·10	0
	Refreshment	1 set	0·15	0
Training of Research assistants (All trial arms)	Research assistant x3	6·6 days	1·91	1·91
	Senior lecturer x1	0·5 days		
	Reader x1	0·5 days		
	Trial Manager x1	4 days		
Total cost per participant			104·64	44·83

^ Design was included as materials couldn't be used wholesale from a previous study and we judged that this may occur in the future following further learning from this trial*Value removed at present to maintain confidentiality
*Data source: Interviews with trial PI and trial manager, review of trial records, diaries, and routine administrative records

For peer review only

Table S2: Components of delivery cost of intervention (Post group)

Components	Resource (from administrative records)	Quantity of resource	Unit cost (data source)	3 months analysis		12 months analysis	
				Total cost	Cost per participant	Total cost	Cost per participant
Envelopes for posting pedometers (including replacement)	Number of envelopes	426	£0·03 (invoice)	£12·78	£0·04	£12·78	£0·04
Stamps for posting pedometer	Number of stamps	426	£2·50 (invoice)	£1,065	£3·14	£1,065	£3·14
Pedometers (including replacements) given to participants	Number of pedometers	426	£1 / £4*(invoice)	£426	£1·26	£1,704	£5·03
Replacement batteries for pedometer	Number of replacement batteries	11	£0·67 (invoice)	£7·37	£0·02	£7·37	£0·02
Patient handbooks	Number of handbooks	339	£0·80 (administrative records)	£271	£0·80	£271	£0·80
Step count diary	Number of diaries	339	£1·30 (administrative records)	£440·70	£1·30	£440·70	£1v30
Total cost per participant					£6·56		£10·33

*£1 was pro rata unit cost for 3 months and £4 is for 12 months. As pedometers were required only for the period of analysis but could be used beyond, their costs were spread over their expected lifetime, following Sharples et al (2014)[1]. As pedometers had an expected lifetime of 2 years, the average cost of pedometer was multiplied by 13[1]/104[2](weeks), in the case of 3 month analysis and 52/104 for the 12 month analysis.

For peer review only

[1] Intervention period in weeks
[2] Life expectancy of pedometer (in weeks)- based experience from PACE lift trial

For peer review only - http://bmjopen.bmj.com/site/about/guidelines.xhtml

Table S3: Components of delivery cost of intervention (Nurse group)

Components	Resource (data source)	Quantity of resource	Unit cost (data source)	3 months analysis		12 months analysis	
				Total cost	Cost per participant	Total cost	Cost per participant
Pedometers given to participants	Number of pedometers (administrative records)	346	£1 / £4* (Invoice)	£346	£1	£1384	£4
Patient handbooks	Number of handbooks (administrative records)	346	£0·80 (administrative records)	£277	£1	£277	£1
Step count diary	Number of diaries (administrative records)	346	£1·30 (administrative records)	£449·80	£1·30	£449·80	£1·30
RAs time to arrange consultation	Time spent by RAs (diary)	50·46 hours	£16·51 (administrative records)	£833·07	£2·41	£833·07	£2·41
Phone calls by RA to arrange consultation	Duration of phone calls (administrative records)	3,027·5 mins	£0·11 (BT tariff)	£333·03	£0·96	£333·03	£0·96
Cost of nurse visit per participant (project database for nurse group)					£43		£42
Total cost per participant					£49·67		£51·67

*£1 was pro rata unit cost for 3 months and £4 is for 12 months.

For peer review only

For peer review only - http://bmjopen.bmj.com/site/about/guidelines.xhtml

Table S4: Costs to participants of participating in interventions and physical activity

Participant costs	Control (n=323)	Post (n=312)	Nurse (n=321)
	£ Mean (SD)		
Intervention related			
Time working out how to use pedometer	0(0)	2 (6)	1 (3)
Time planning how to increase walking/step count	0(0)	5 (15)	3 4)
Time filling in PACE-UP diary	0(0)	51 (80)	58 (122)
Parking fees to visit nurse	0(0)	0(0)	0·11 (0.73)
Time spent in consultation with nurse	0(0)	0(0)	10 (5)
Time travelling (irrespective of mode of transport) to visit nurse	0(0)	0(0)	11 (10)
Transportation cost (for those who took public transport) of attending the nurse visit	0(0)	0(0)	0·13 (1.33)
Time waiting time prior to consultation with nurse	0(0)	0(0)	3 (4)
Child care during nurse visits	0(0)	0(0)	0·3 (3.21)
Personal costs of participation in physical activity	411 (817)	492 (1,293)	333 (684)
Personal costs from falls/ fractures/ sprains/ injuries	17 (103)	22 (184)	6 (40)

For peer review only

For peer review only - http://bmjopen.bmj.com/site/about/guidelines.xhtml

Table S5: Health service use by trial arm with unit costs

Health service use	Trial arm (Qty)			Unit cost (£) Weighted average (Q1 – Q3)	Source of unit cost
	Control n=323	Post n=312	Nurse n=321		
**Outpatient referrals (total)[2]**	**164**	**158**	**186**		
Opthalmology	10	18	15	86 (70-99)	DH (2015) National Reference Costs
Urology	4	3	6	99 (76-116)	
General medicine	4	0	2	157 (120-187)	
ENT	9	6	12	92 (70-109)	
Podiatry	9	7	7	44 (27-45)	
Trauma & orthopaedics	14	13	10	113 (88-133)	
Physiotherapy	26	33	37	46 (35-50)	
Nephrology	0	1	0	145 (94-178)	
Oral surgery	0	2	0	115 (85-142)	
Gynaecology	6	7	14	134 (104-164)	
Audiology	4	6	7	104 (55-174)	
Colorectal surgery	1	5	1	117 (83-135)	
Neurology	8	8	5	174 (136-204)	
Cardiology	12	5	4	131 (92-154)	
Gastroenterology	6	2	6	130 (99-153)	
Rheumatology	4	6	7	135 (99-150)	
Dermatology	1	8	7	98 (74-109)	
General surgery	4	1	3	125 (98-165)	
Endocrinology	2	1	2	144 (100-167)	
Neurosurgery	2	0	0	181 (138-228)	
Oncology	8	5	11	133 (97-165)	
Psychotherapy	1	0	0	100 (47-217)	
Respiratory medicine	4	6	3	150 (107-181)	
Clinical neurophysiology	2	0	1	165 (107-197)	
Programmed pulmonary rehab	0	0	1	20 (12-31)	
Pain management	2	0	4	135 (82-164)	
Allergy service	0	1	0	149 (126-175)	
Dietetics	2	2	3	62 (38-76)	
Vascular surgery	2	1	4	149 (100-176)	
Mental illness	1	1	1	234 (181-256)	
Clinical Genetics	1	0	1	429 (248-601)	
Clinical Haematology	2	1	0	160 (93-189)	
Spinal surgery services	0	1	0	142 (112-164)	
Maxillo-facial surgery	0	0	1	111 (70-133)	
Plastic surgery	1	1	1	93 (68-109)	
Clinical immunology	0	1	0	215 (140-243)	
Interventional radiology	1	0	0	192 (88-260)	
Breast surgery	9	4	5	139 (103-166)	
Tropical medicine	0	1	0	202 (203-203)	
Clinical psychology	1	0	3	177 (116-245)	
Old age psychiatry	0	1	2	108 (108-108)	
Referral to Accident & Emergency	1	0	0	135 (54-166)	
**Community based referrals (total)[3]**	**27**	**19**	**21**		
District nurse	1	3	2	39 (31-43)	PSSRU
Community Podiatrist	4	3	8	42 (35-58)	PSSRU
Community Dietitian	0	2	0	80 (53-96)	DH (2015) National Reference Costs
Smoking cessation (Nurse)	5	3	4	14	15.5 mins nurse time (Curtis 2014)
Healthy lifestyle (Nurse)	0	2	0	14	15.5 mins nurse time (Curtis 2014)
Community Gynaecologist	5	1	0	134 (104-164)	DH (2014) National Reference Costs
Community Physiotherapist	7	4	1	52 (44-58)	(Curtis 2014)
Community Diabetic	1	0	0	69 (38-93)	DH (2015) National Reference Costs
DESMOND diabetes programme	4	0	6	230	Gillett et al (2010) (inflated to 2014)
Expert Patient Programme	0	1	0	302	Richardson et al (2008) (inflated to 2014)
**Primary Care – excludes practice**	**2074**	**1748**	**2094**		

For peer review only

For peer review only - http://bmjopen.bmj.com/site/about/guidelines.xhtml

Health service use	Trial arm (Qty)			Unit cost (£) Weighted average (Q1 – Q3)	Source of unit cost
	Control n=323	Post n=312	Nurse n=321		
**visits related to the delivery and participation in intervention (total)**[1]					
GP (11.7mins)	1743	1436	1729	42	(Curtis 2014)
GP nurse (15.5mins)	331	312	365	14	(Curtis 2014)
**A&E visit**[4]	49	36	46	124	DH (2015) National Reference Costs
**Non- Elective hospital admissions (total)**[5,6]	**12**	**4**	**20**		
Biliary acute pancreatitis	0	0	3	2037 (1247-2492)	DH (2015) National Reference Costs
Cardiac catheterisation for coronary artery disease	1	0	1	2643 (1980-3028)	
Chest pain	0	1	0	490 (370-563)	
Abdominal pain	0	0	1	718 (922 -1298)	
Acute ST segment elevation myocardial infarction	2	0	0	1497 (1102-1740)	
Transient ischaemic attack	0	0	1	878 (643-994)	
Guillain-Barre syndrome	0	0	1	1571 (1069-1792)	
Pneumonia	1	0	0	1894 (1406-2238)	
Epilepsy	1	0	0	1125 (788-1266)	
Stroke and cerebrovascular accident	1	0	0	2817 (2018-3396)	
UTI	0	0	1	1530 (1187-1755)	
Detached Retina	0	0	1	908 (303-1935)	
Anxiety states	0	0	1	1393 (984-1628)	
Infective endocarditis in diseases EC, NOS	1	0	0	4480 (2351-5906)	
Acute appendicitis	0	0	1	3017 (2459-3365)	
IUD removed	0	0	1	1780 (1142-2135)	
Ankle fracture	1	0	0	3762 (3109-4271)	
no procedure (NES)	4	3	8	611 (408-726)	
**Elective hospital admissions (total)**[5,7]	**10**	**2**	**3**		
Cardiac catheterisation	2	0	0	2086 (1185-2709)	DH (2015) National Reference Costs
Percut tranlum balloon angioplasty mult coronary	1	0	0	1813 (880-2233)	
Inguinal hernia	0	1	0	2121 (1682-2392)	
Coronary artery bypass graft operations	0	1	0	9310 (7369-9929)	
Laparoscopic cholecystectomy	3	0	0	2567 (2082-2924)	
Endarterectomy of femoral artery NEC	0	0	2	6028 (4593-7209)	
Malignant neoplasm of female breast for chemotherapy	1	0	0	1780 (856-2139)	
Endarterectomy of carotid artery NEC	1	0	0	3911 (2986-4497)	
Neurophysiological operation NOS	2	0	0	1497 (1111-2118)	
Ovarian Cancer	0	0	1	1469 (741-1966)	
Total resource use (All HSU)	2336	1967	2370		

Unit costs are rounded to the nearest whole number and presented in the 2013/14 price year. The health service use presented in this table refers to the base case sample. All the data are based on participant-specific GP records for the trial period with different assumptions and approaches for costing by type of service use:

[1]*Primary care*: GP visits 11.7 minutes; Nurse visits 15·5 minutes;

[2]*Outpatient referrals*: where appropriate, linked to outpatient service descriptions in the reference costs (and reviewed by principal investigator) and a weighted (by throughout) average for consultant/non-consultant led attendances taken; referrals to private sector excluded (n=1);

[3]community referral services costed as referenced; if service use was unclear, an NHS hospital out-patient department was assigned by the principal investigator;

[4]*A&E visit*: as reason for A&E visits was not recorded, an average A&E visit cost for 2013-14 was assigned.

For peer review only - http://bmjopen.bmj.com/site/about/guidelines.xhtml

[5] *Hospital admissions*: The principal investigator (blind to study group) reviewed all hospital admissions, and provided either a 'best guess diagnosis/procedure' or listed 'unknown' (n=2). As details on the type of procedure or severity of the symptoms were not available, a weighted (by activity) average of all of the possible scores/procedures was used to derive average cost for elective.

[6] The unit cost for the emergency admissions are a weighted average of the non-elective short stay and non-elective long stay admissions, as the length of stay was unclear.

[7] Hospital admissions without a procedure were treated as non-elective short stay admissions (one day or less). Where hospital admission code was unclear the diagnosis was reviewed by the PI for advice on the nearest appropriate code.

For peer review only

Figure S1: Illustration of pathways within the long-term cost-effectiveness model (Anokye et al 2014a)

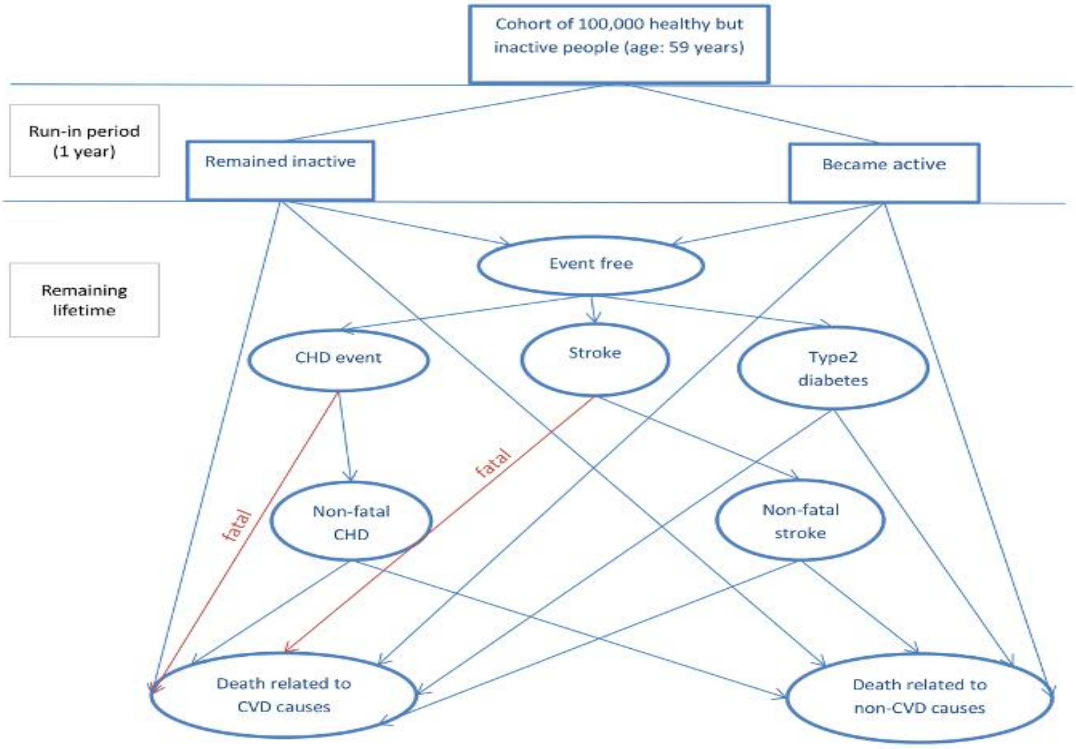

iew only

For peer review only - http://bmjopen.bmj.com/site/about/guidelines.xhtml

Table S6: Parameter values for long-term cost-effectiveness model

Parameter		Value	Source of data
Relative risks of:			
Becoming active (at year 1)*:	Postal vs control	1·8 (95% CI: 1·4, 2.3)	PACE-UP trial data
	Nurse vs control	1·7 (95% CI: 1·3, 2.2)	
	Nurse vs postal	0·9 (95% CI: 0·7, 1.3)	
Disease (active vs inactive)	CHD	0·90	Hu et al (2007)
	Stroke	0·86	Hu et al (2005)
	Diabetes	0·67	Hu et al (2003)
Non-CVD mortality after:	Non-fatal CHD	1·71	Bronum-Hansen et al (2001)
	Non-fatal Stroke	1·71	
	Diabetes	1·49	Preis et al (2009)
CVD mortality after:	Non-fatal CHD	3·89	Bronum-Hansen et al (2001)
	Non-fatal Stroke	3·89	
	Diabetes	2·61	Preis et al (2009)
CHD fatalities	59-64	11·55%	Ward et al (2005)
	65-74	21·07%	
	75+	14·76%	
Stroke fatalities	55-64	23·28%	Ward et al (2005)
	65-74	23·47%	
	75+	23·42%	
CHD incidence	59-64	0·63%	Ward et al (2005); NCGC (2011)
	65-74	0·97%	
	75+	0·97%	
Stroke incidence	59-64	0·29%	
	65-74	0·69%	
	75+	1·43%	
Diabetes incidence	59	0·06%	Gonzalez et al (2009)
	60-69	0·10%	
	70-79	0·11%	
	80+	0·11%	
Age-specific quality of life	59-64	0·82	Health Survey for England (2011)
	65-74	0·78	
	75+	0·72	
Health state utility weight	Healthy	1·00	Ward et al (2005); NCGC (2011)
	CHD 1st event	0·80	
	post CHD 1st event	0·92	
	Stroke 1st event	0·63	
	post stroke 1st event	0·65	
	Diabetes	0·90	
	Short term psychological benefit of achieving 150 mins of MVPA per week	0·01	PACE UP trial data
Annual costs	Control	£467 (95% CI 365 to569)	PACE UP trial data
	Postal	£376 (95% CI 307 to445)	

For peer review only - http://bmjopen.bmj.com/site/about/guidelines.xhtml

Parameter		Value	Source of data
	Nurse	£593 (95% CI 473 to714)	
	CHD 1st event	£4,248	NCGC (2011)
	post CHD 1st event	£485	
	Stroke 1st event	£10,968	
	post stroke 1st event	£2,409	
	Diabetes	£979	

*Relative risks (RR) for achieving at least 150 minutes of MVPA in ≥10 minute bouts at 12 months were estimated from odds ratios (OR) using the formula OR / {(1-P_{ref}) + (P_{ref} *OR)} where P_{ref} is the proportion of all subjects achieving 150 minutes of MVPA in ≥10 minute bouts at baseline i.e. 218/1023 = 0.21. The odds ratios had been derived from a logistic regression model in which the dependent variable, achieving 150 minutes of MVPA in bouts of ≥10 minutes at 12 months, was regressed on baseline minutes of MVPA in bouts of ≥10 minutes, month of baseline accelerometry, day order of wear, day of week, age, gender, general practice and treatment group, with household as a cluster.

For peer review only

For peer review only - http://bmjopen.bmj.com/site/about/guidelines.xhtml

Figure S2: Cost-effectiveness plane for postal vs control at 12 months

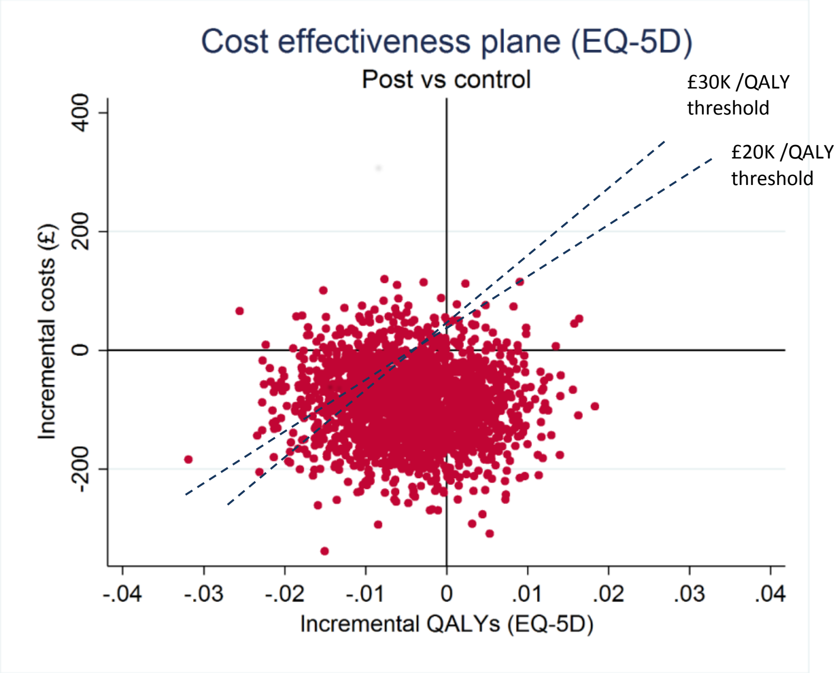

Figure S3: Cost-effectiveness plane for nurse vs control at 12 months

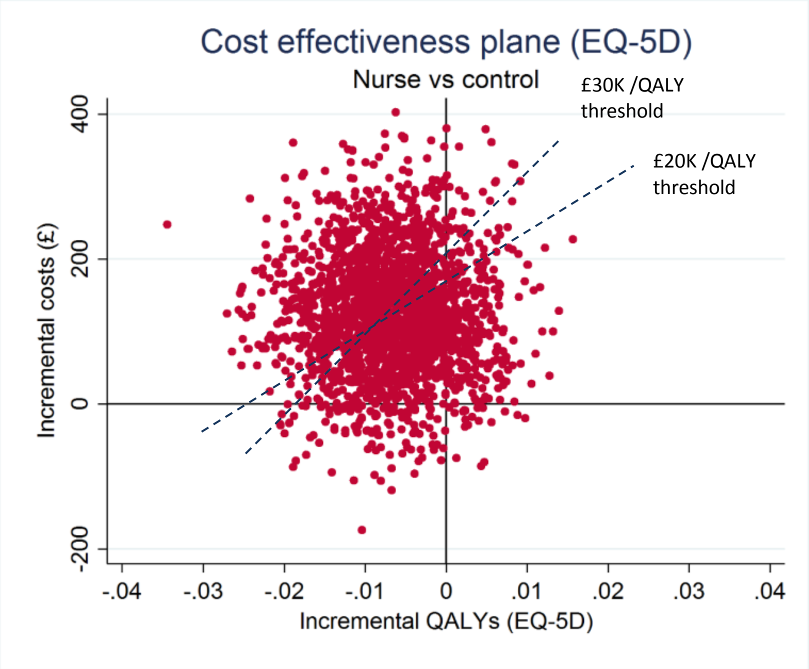

For peer review only - http://bmjopen.bmj.com/site/about/guidelines.xhtml

Figure S4: Cost-effectiveness plane for nurse vs post at 12 months

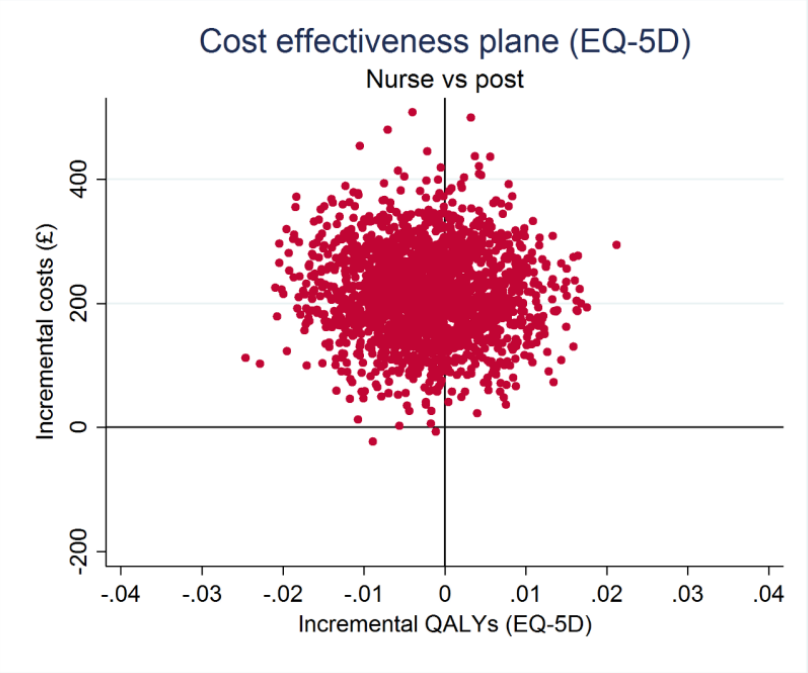

For peer review only - http://bmjopen.bmj.com/site/about/guidelines.xhtml

Table S7: Within trial sensitivity analyses (at 12 months)

Parameter	Post vs Control			Nurse vs Control			Nurse vs Post		
	Incremental cost (£)	Incremental QALY	ICER	Incremental cost(£)	Incremental QALY	ICER	Incremental cost (£)	Incremental QALY	ICER
	Mean (95% CI)			Mean (95% CI)			Mean (95% CI)		
Base case	-91* (-215, 3)	-0.0043 (-0.0172, 0.0087)	Less costly but less effective than control	126 (-37, 290)	-0.0066 (-0.0201, 0.0068)	Intervention dominated by control	217 (8, 354)	-0.0024 (-0.0156, 0.0109)	Nurse dominated by Post
Whole sample (all randomised)	-40 (-169, -89)	-0.0070 (-0.0195, 0.0054)	Less costly but less effective than control	150 (-6, 306)	-0.0093 (-0.0222, 0.0036)	Intervention dominated by control	190 (48, 332)	-0.0023 (-0.0148, 0.0102)	Nurse dominated by Post
Health service use including only GP data on referrals and admissions	-55 (-166, -56)	-0.0043 (-0.0172, 0.0087)	Less costly but less effective than control	129 (-17, 275)	-0.0066 (-0.020, 0.0068)	Intervention dominated by control	184 (61, 307)	-0.0024 (-0.0156, 0.0109)	Nurse dominated by Post
Health service use including only self-reported serious adverse effects	21 (-65, 107)	-0.0043 (-0.0172, 0.0087)	Intervention dominated by control	144 (65, 224)	-0.0066 (-0.020, 0.0068)	Intervention dominated by control	123 (47, 200)	-0.0024 (-0.0156, 0.0109)	Nurse dominated by Post
Health service use including only GP data on adverse effects	-11 (-107, 85)	-0.0043 (-0.0172, 0.0087)	Less costly but less effective than control	64 (-15, 142)	-0.0066 (-0.020, 0.0068)	Intervention dominated by control	74 (13, 135)	-0.0024 (-0.0156, 0.0109)	Nurse dominated by Post
Excluding all health service use cost	55.2 (55, 55.4)	-0.0043 (-0.0172, 0.0087)	Intervention dominated by control	156.2 (-154, 158)	-0.0066 (-0.0201, 0.0068)	Intervention dominated by control	101 (99, 103)	-0.0024 (-0.0156, 0.0109)	Nurse dominated by Post
Exclusion of missing data**	-91 (-215, 33)	-0.0088 (-0.0231, 0.0055)	Less costly but less effective than control	126 (-37, 290)	-0.0078 (-0.0233, 0.0076)	Intervention dominated by control	217 (8, 354)	0.0009 (-0.0141, 0.0160)	More costly but less effective than control (ICER:£241k)
Changing cost perspective (both participants (all participant costs) and NHS costs)	36 (-177, 250)	-0.0043 (-0.0172, 0.0087)	Intervention dominated by control	107 (-97, 311)	-0.0066 (-0.020, 0.0068)	Intervention dominated by control	71 (-150, 291)	-0.0024 (-0.0156, 0.0109)	Nurse dominated by Post
Changing cost perspective (both participants (part)[3] and NHS costs)	-22 (-235, 191)	-0.0043 (-0.0172, 0.0087)	Less costly but less effective than control	47 (-157, 250)	-0.0066 (-0.020, 0.0068)	Intervention dominated by control	69 (-152, 289)	-0.0024 (-0.0156, 0.0109)	Nurse dominated by Post
Combination of excluding all health service use cost and including all participants costs (minus health service use cost borne by participants)	179 (-1, 361)	-0.0043 (-0.0172, 0.0087)	Intervention dominated by control	153 (24, 281)	-0.0066 (-0.020, 0.0068)	Intervention dominated by control	-27 (-203, 149)	-0.0024 (-0.0156, 0.0109)	Less costly but less effective than control
Pedometer lasts for 1 year (equivalent to pedometers not being re-usable and full	-86 (-210, 38)	-0.0043 (-0.0172,	Less costly but less effective than control	130 (-33, 294)	-0.0066 (-0.0201,	Intervention dominated by	216 (80, 353)	-0.0024 (-0.0156,	Nurse dominated by

* This excludes time costs of working out how to use pedometer, diary, and planning to increase work

** The pattern of missing data for the base case analysis was multivariate (i.e. some but not all variables had data missing for some participants). The amount of missing data, where observed, was less than 5% except for EQ5D scores (baseline data: 5% (n=51); 3 months data: 7% (n=67); 12 months data: 8%(n=74)).

For peer review only - http://bmjopen.bmj.com/site/about/guidelines.xhtml

Parameter	Post vs Control			Nurse vs Control			Nurse vs Post		
	**Incremental cost (£)**	**Incremental QALY**	**ICER**	**Incremental cost(£)**	**Incremental QALY**	**ICER**	**Incremental cost (£)**	**Incremental QALY**	**ICER**
	**Mean (95% CI)**			**Mean (95% CI)**			**Mean (95% CI)**		
Base case	-91* (-215, 3)	-0.0043 (-0.0172, 0.0087)	Less costly but less effective than control	126 (-37, 290)	-0.0066 (-0.0201, 0.0068)	Intervention dominated by control	217 (8, 354)	-0.0024 (-0.0156, 0.0109)	Nurse dominated by Post
cost of pedometer borne in year 1)		0.0087)			0.0068)	control		0.0109)	Post
Pedometer lasts for 4 years (double length of life considered in base case)	-93 (-218, 31)	-0.0043 (-0.0172, 0.0087)	Less costly but less effective than control	124 (-39, 287)	-0.0066 (-0.0201, 0.0068)	Intervention dominated by control	218 (81, 354)	-0.0024 (-0.0156, 0.0109)	Nurse dominated by Post

For peer review only

References

Anokye NK., Lord J., Fox-Rushby J. Is brief advice in primary care a cost-effective way to promote physical activity?, *British Journal of Sports Medicine* 2014; **48 (3)** : 202–206

Brønnum-Hansen H, Davidsen M, Thorvaldsen P. Long-term survival and causes of death after stroke. *Stroke* 2001; **32**: 2131–6.

Curtis LA: Unit costs for health and social care Personal Social Services Research Unit, University of Kent: Canterbury; 2014.

De Cocker K, De Bourdeaudhuij I, Brown W, Cardon G. Moderators and mediators of pedometer use and step count increase in the "10,000 Steps Ghent" intervention. *International Journal of Behavioral Nutrition and Physical Activity* 2009; **6**:3 http://dx.doi.org/10.1186/1479-5868-6-3

Department of Health (2015). NHS Reference Costs 2013 to 2014. Available from:https://www.gov.uk/government/publications/nhs-reference-costs-2013-to-2014

Gillett M, Dallosso HM, Dixon S, et al. Delivering the diabetes education and self- management for ongoing and newly diagnosed (DESMOND) programme for people with newly diagnosed type 2 diabetes: cost effectiveness analysis. *BMJ 2010*: 341:c4093

Gonzalez ELM, Johansson S, Wallander MA. Trends in the prevalence and incidence of diabetes in the UK, 1996–2005. *J Epidemiol Community Health* 2009; **63**:332–6.

Hu G, Jousilahti P, Borodulin K, et al. Occupational, commuting and leisure-time physical activity in relation to coronary heart disease among middle-aged Finnish men and women. *Atherosclerosis* 2007; **194**:490–7

Hu G, Sarti C, Jousilahti P, et al. Leisure time, occupational, and commuting physical activity and the risk of stroke. *Stroke* 2005; **36**:1994–9.

Hu G, Qiao Q, Silventoinen K, et al. Occupational, commuting, and leisure-time physical activity in relation to risk for type 2 diabetes in middle-aged Finnish men and women. *Diabetologia* 2003; **46**:322–29

Health Survey for England, 2011 National Centre for Social Research, University College London- Department of Epidemiology and Public Health. [computer file]. *3rd Edition.* Colchester, Essex: UK Data Archive [distributor], July 2011. SN: 6397

National Clinical Guideline Centre (NCGC). Hypertension: the clinical management of primary hypertension in adults. Clinical guideline: methods, evidence and recommendations. London: National Institute for Health and Clinical Excellence. 2011. Ref Type: Report.

Preis SR, Hwang SJ, Coady S, et al. Trends in all-cause and cardiovascular disease mortality among women and men with and without diabetes mellitus in the Framingham Heart Study, 1950 to 2005. *Circulation* 2009; **119**:1728–35.

Richardson G, Kennedy A, Reeves D, et al. A Cost effectiveness of the Expert Participants Programme (EPP) for participants with chronic conditions. *Journal of Epidemiology and Community Health* 2008; **62**: 361–367

For peer review only - http://bmjopen.bmj.com/site/about/guidelines.xhtml

Sharples L, Glover M, Bennett M, Jordan J, Clutterbuck-James A, Davies M, Smith I, Cameron M, Chadwick R, Oscroft N, Morrell M, Fox-Rushby J, Pittman M, Quinnell T (2014) TOMADO: A Crossover Randomised Controlled Trial of Oral Mandibular Advancement Devices for Obstructive Sleep Apnoea-Hypopnoea *Health Technology Assessment 18, 67, http://www.journalslibrary.nihr.ac.uk/hta/volume-18/issue-67#*

Ward S, Jones ML, Pandor A, et al. Statins for the prevention of coronary events. London. National Institute for Clinical Excellence 2005; Ref Type: Report World Health Organisation (2010) Global recommendations on physical activity for health http://www.who.int/dietphysicalactivity/publications/9789241599979/en/

For peer review only

CHEERS Checklist

Items to include when reporting economic evaluations of health interventions

The **ISPOR CHEERS Task Force Report**, *Consolidated Health Economic Evaluation Reporting Standards (CHEERS)—Explanation and Elaboration: A Report of the ISPOR Health Economic Evaluations Publication Guidelines Good Reporting Practices Task Force,* provides examples and further discussion of the 24-item CHEERS Checklist and the CHEERS Statement. It may be accessed via the *Value in Health* or via the ISPOR Health Economic Evaluation Publication Guidelines – CHEERS: Good Reporting Practices webpage: http://www.ispor.org/TaskForces/EconomicPubGuidelines.asp

Section/item	Item No	Recommendation	Reported on page No/ line No
**Title and abstract**			
Title	1	Identify the study as an economic evaluation or use more specific terms such as "cost-effectiveness analysis", and describe the interventions compared.	P1 L1-2
Abstract	2	Provide a structured summary of objectives, perspective, setting, methods (including study design and inputs), results (including base case and uncertainty analyses), and conclusions.	P2 L1-39
**Introduction**			
Background and objectives	3	Provide an explicit statement of the broader context for the study. Present the study question and its relevance for health policy or practice decisions.	P4 all & P5L4-5
**Methods**			
Target population and subgroups	4	Describe characteristics of the base case population and subgroups analysed, including why they were chosen.	P4L1-21 & P2L11-12
Setting and location	5	State relevant aspects of the system(s) in which the decision(s) need(s) to be made.	P2L23 - P3L5
Study perspective	6	Describe the perspective of the study and relate this to the costs being evaluated.	P6L14, P6L29-30
Comparators	7	Describe the interventions or strategies being compared and state why they were chosen.	P5 L16-21
Time horizon	8	State the time horizon(s) over which costs and consequences are being evaluated and say why appropriate.	P6L4-12&21, P6L16
Discount rate	9	Report the choice of discount rate(s) used for costs and outcomes and say why appropriate.	P6L7, P7L17
Choice of health outcomes	10	Describe what outcomes were used as the measure(s) of benefit in the evaluation and their relevance for the type of analysis performed.	P6L10-14, P7L23-29
Measurement of effectiveness	11a	*Single study-based estimates:* Describe fully the design features of the single effectiveness study and why the single study was a sufficient source of clinical effectiveness data.	P4L14-16

For peer review only

	11b	*Synthesis-based estimates:* Describe fully the methods used for identification of included studies and synthesis of clinical effectiveness data.	P7L15-P8L14, ref 13
Measurement and valuation of preference based outcomes	12	If applicable, describe the population and methods used to elicit preferences for outcomes.	Na
Estimating resources and costs	13a	*Single study-based economic evaluation:* Describe approaches used to estimate resource use associated with the alternative interventions. Describe primary or secondary research methods for valuing each resource item in terms of its unit cost. Describe any adjustments made to approximate to opportunity costs.	P5L24-P6L8 TablsS1-5
	13b	*Model-based economic evaluation:* Describe approaches and data sources used to estimate resource use associated with model health states. Describe primary or secondary research methods for valuing each resource item in terms of its unit cost. Describe any adjustments made to approximate to opportunity costs.	P7L25, P8L7-8 Ref 13
Currency, price date, and conversion	14	Report the dates of the estimated resource quantities and unit costs. Describe methods for adjusting estimated unit costs to the year of reported costs if necessary. Describe methods for converting costs into a common currency base and the exchange rate.	P6L6-7, P7L16
Choice of model	15	Describe and give reasons for the specific type of decision-analytical model used. Providing a figure to show model structure is strongly recommended.	P7L15-6,FigS1, P8Sup1
Assumptions	16	Describe all structural or other assumptions underpinning the decision-analytical model.	SuplTS6(p10-11)
Analytical methods	17	Describe all analytical methods supporting the evaluation. This could include methods for dealing with skewed, missing, or censored data; extrapolation methods; methods for pooling data; approaches to validate or make adjustments (such as half cycle corrections) to a model; and methods for handling population heterogeneity and uncertainty.	P6L16-P7L12, P8L18-25
**Results**			
Study parameters	18	Report the values, ranges, references, and, if used, probability distributions for all parameters. Report reasons or sources for distributions used to represent uncertainty where appropriate. Providing a table to show the input values is strongly recommended.	P8L2-15, TblS1-S5
Incremental costs and outcomes	19	For each intervention, report mean values for the main categories of estimated costs and outcomes of interest, as well as mean differences between the comparator groups. If applicable, report incremental cost-effectiveness ratios.	Tabl1-3, P8L29-P10L2, P10L20-27
Characterising uncertainty	20a	*Single study-based economic evaluation:* Describe the effects of sampling uncertainty for the estimated incremental cost and incremental effectiveness parameters, together with the impact	P10L4-17, Fig1&2, TblS7

For peer review only

		of methodological assumptions (such as discount rate, study perspective).	Fig S2-4, P11L1-16
	20b	*Model-based economic evaluation:* Describe the effects on the results of uncertainty for all input parameters, and uncertainty related to the structure of the model and assumptions.	Na
Characterising heterogeneity	21	If applicable, report differences in costs, outcomes, or cost-effectiveness that can be explained by variations between subgroups of patients with different baseline characteristics or other observed variability in effects that are not reducible by more information.	P11L19-P12L15
**Discussion** Study findings, limitations, generalisability, and current knowledge	22	Summarise key study findings and describe how they support the conclusions reached. Discuss limitations and the generalisability of the findings and how the findings fit with current knowledge.	P15L1-7
**Other**			
Source of funding	23	Describe how the study was funded and the role of the funder in the identification, design, conduct, and reporting of the analysis. Describe other non-monetary sources of support.	P15L10-12
Conflicts of interest	24	Describe any potential for conflict of interest of study contributors in accordance with journal policy. In the absence of a journal policy, we recommend authors comply with International Committee of Medical Journal Editors recommendations.	

For consistency, the CHEERS Statement checklist format is based on the format of the CONSORT statement checklist

The **ISPOR CHEERS Task Force Report** provides examples and further discussion of the 24-item CHEERS Checklist and the CHEERS Statement. It may be accessed via the *Value in Health* link or via the ISPOR Health Economic Evaluation Publication Guidelines – CHEERS: Good Reporting Practices webpage: http://www.ispor.org/TaskForces/EconomicPubGuidelines.asp

The citation for the CHEERS Task Force Report is:

Husereau D, Drummond M, Petrou S, et al. Consolidated health economic evaluation reporting standards (CHEERS)—Explanation and elaboration: A report of the ISPOR health economic evaluations publication guidelines good reporting practices task force. Value Health 2013;16:231-50.

For peer review only - http://bmjopen.bmj.com/site/about/guidelines.xhtml
